# Phylogenetic Analysis of Pelecaniformes (Aves) Based on Osteological Data: Implications for Waterbird Phylogeny and Fossil Calibration Studies

**DOI:** 10.1371/journal.pone.0013354

**Published:** 2010-10-14

**Authors:** Nathan D. Smith

**Affiliations:** 1 Committee on Evolutionary Biology, University of Chicago, Chicago, Illinois, United States of America; 2 Department of Geology, The Field Museum of Natural History, Chicago, IIllinois, United States of America; American Museum of Natural History, United States of America

## Abstract

**Background:**

Debate regarding the monophyly and relationships of the avian order Pelecaniformes represents a classic example of discord between morphological and molecular estimates of phylogeny. This lack of consensus hampers interpretation of the group's fossil record, which has major implications for understanding patterns of character evolution (e.g., the evolution of wing-propelled diving) and temporal diversification (e.g., the origins of modern families). Relationships of the Pelecaniformes were inferred through parsimony analyses of an osteological dataset encompassing 59 taxa and 464 characters. The relationships of the Plotopteridae, an extinct family of wing-propelled divers, and several other fossil pelecaniforms (*Limnofregata*, *Prophaethon*, *Lithoptila*, ?*Borvocarbo stoeffelensis*) were also assessed. The antiquity of these taxa and their purported status as stem members of extant families makes them valuable for studies of higher-level avian diversification.

**Methodology/Principal Findings:**

Pelecaniform monophyly is not recovered, with Phaethontidae recovered as distantly related to all other pelecaniforms, which are supported as a monophyletic Steganopodes. Some anatomical partitions of the dataset possess different phylogenetic signals, and partitioned analyses reveal that these discrepancies are localized outside of Steganopodes, and primarily due to a few labile taxa. The Plotopteridae are recovered as the sister taxon to Phalacrocoracoidea, and the relationships of other fossil pelecaniforms representing key calibration points are well supported, including *Limnofregata* (sister taxon to Fregatidae), *Prophaethon* and *Lithoptila* (successive sister taxa to Phaethontidae), and ?*Borvocarbo stoeffelensis* (sister taxon to Phalacrocoracidae). These relationships are invariant when ‘backbone’ constraints based on recent avian phylogenies are imposed.

**Conclusions/Significance:**

Relationships of extant pelecaniforms inferred from morphology are more congruent with molecular phylogenies than previously assumed, though notable conflicts remain. The phylogenetic position of the Plotopteridae implies that wing-propelled diving evolved independently in plotopterids and penguins, representing a remarkable case of convergent evolution. Despite robust support for the placement of fossil taxa representing key calibration points, the successive outgroup relationships of several “stem fossil + crown family” clades are variable and poorly supported across recent studies of avian phylogeny. Thus, the impact these fossils have on inferred patterns of temporal diversification depends heavily on the resolution of deep nodes in avian phylogeny.

## Introduction

Several aspects of the avian order Pelecaniformes [1] make them desirable as a study system for phylogenetic research. They are a group that is relatively tractable in terms of diversity, with the traditional content of the order comprising approximately 57 species in six families. These include 3 species of tropicbirds (Phaethontidae), 7 species of pelicans (Pelecanidae), 5 species of frigatebirds (Fregatidae), 10 species of gannets and boobies (Sulidae), 4 species of darters (Anhingidae), and 28 species of cormorants (Phalacrocoracidae). The latter three families comprise a clade commonly referred to as ‘core’ Pelecaniformes [2], but also known as Suloidea (superfamily Suloidea *sensu* Cracraft [3]; also considered parvorder Sulida [4], or suborder Sulae [5]). Though pelecaniforms have many similarities in life history and ecology (e.g., all are primarily piscivorous, coastal waterbirds), there are also extreme differences between clades (e.g., Fregatidae are kleptoparasitic soarers; Sulidae and *Pelecanus occidentalis* are plunge-divers; Anhingidae and Phalacrocoracidae are foot-propelled divers), which make them appealing for studying morphological character evolution. The Pelecaniformes have also been utilized as a model system for a variety of evolutionary studies, including host-parasite co-evolution [2], biogeography of speciation [6]; adaptive evolution and phylogenetic constraint [7]; functional morphology [8], [9]; and studies of behavior and social displays [10]–[12]. Diverse comparative evolutionary studies such as these require detailed knowledge of phylogenetic relationships for rigorous hypothesis testing [13], [14], making the current analysis particularly relevant.

**Figure 1:**
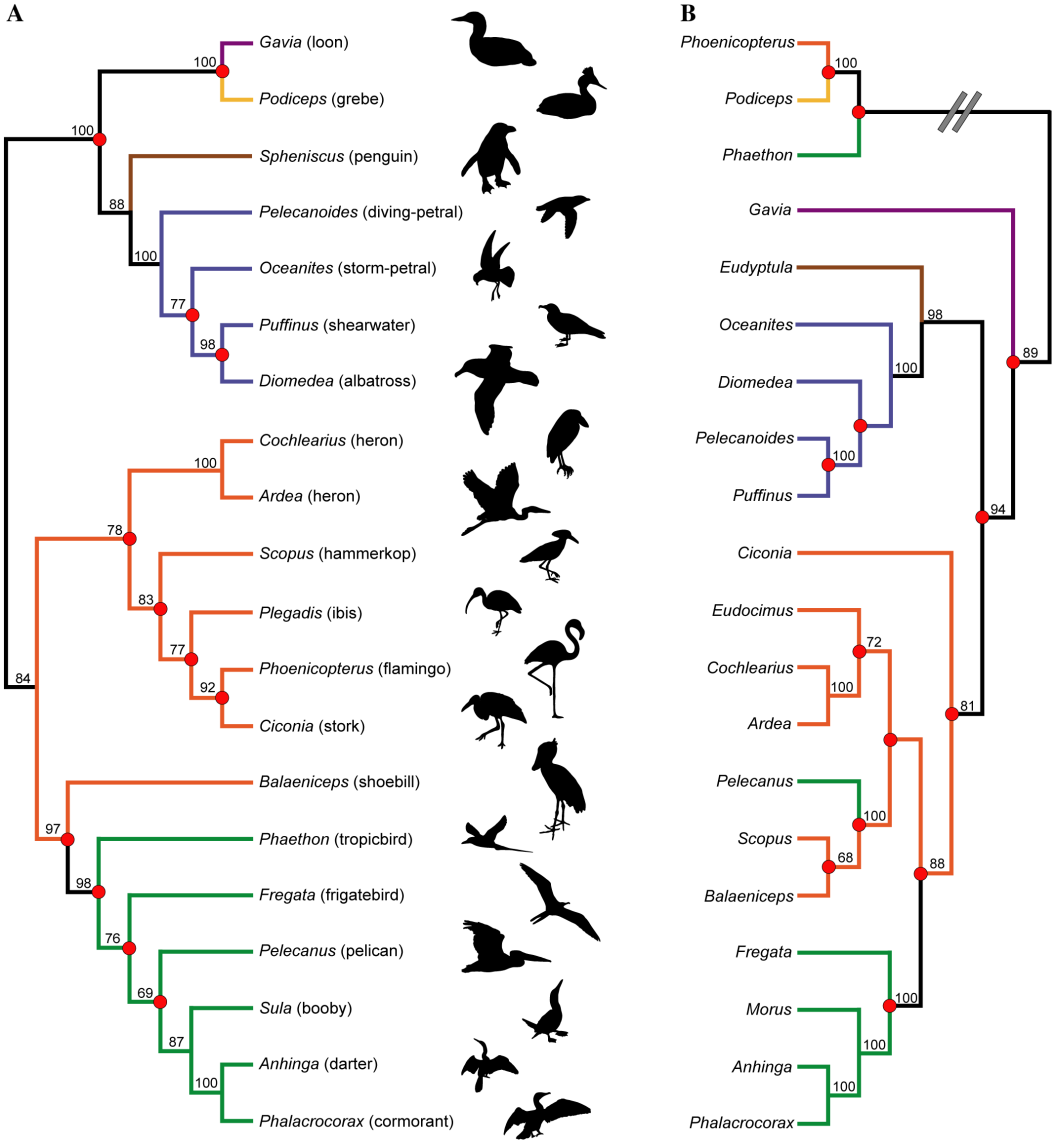
Recent estimates of higher-level waterbird phylogeny based on morphological and molecular datasets. (A) Topology recovered in the parsimony analysis of morphological data by Livezey and Zusi [4], with common names and silhouettes of taxa indicated to the right. (B) Topology recovered in the maximum likelihood analysis of molecular data by Hackett et al. [29]. Double hash marks in (B) indicate that the clade containing *Phaethon*, *Podiceps*, and *Phoenicopterus* is actually recovered as distantly related to the waterbird clade (i.e., is not its sister-taxon). Branch colors represent traditional avian orders: Gaviiformes (purple); Podicipediformes (yellow); Sphenisciformes (brown); Procellariiformes (blue); Ciconiiformes (orange); Pelecaniformes (green). Note that for the purposes here, *Phoenicopterus* is considered as being traditionally allied with Ciconiiformes, though it could also be treated as the monotypic order Phoenicopteriformes. Several taxa that were not shared by both datasets were pruned from the trees. Bootstrap values ≥70% are indicated above nodes. Nodes that conflict between topologies are indicated with red circles.

The Pelecaniformes are especially interesting from a phylogenetic perspective, as previous studies have demonstrated a blend of congruence (e.g., monophyly of Suloidea, monophyly of individual pelecaniform families) and incongruence (e.g., monophyly/polyphyly of Pelecaniformes, relationships of Phaethontidae, relationships of Pelecanidae) between molecular and morphological datasets [3], [4], [15]–[29] (Figure 1). Due to the low statistical support for many relationships within Pelecaniformes and the waterbird clade, as well as enduring conflicts between datasets, an emerging consensus of recent studies is that additional data and revision are needed for both molecular and morphological datasets.

The monophyly of Pelecaniformes as traditionally defined (i.e., a clade including tropicbirds, frigatebirds, pelicans, sulids, darters, and cormorants) has been extremely controversial, even prior to the advent of molecular systematics (see review in Livezey and Zusi [4]). Interestingly, some of the earliest (e.g., [3]), and most recent (e.g., [4]), morphological phylogenetic analyses of the Pelecaniformes have supported ordinal monophyly, while several other recent studies have suggested that the group is paraphyletic or polyphyletic [22], [23], [25], [26], [30]. Molecular evidence for the non-monophyly of the traditional order Pelecaniformes has become increasingly well supported [27]–[29], [31]. This is usually due to the aberrant tropicbirds, Phaethontidae, being recovered as distantly related to the group, and the alliance of the pelicans (Pelecanidae), with the enigmatic shoebill (*Balaeniceps*), and hammerkop (*Scopus*) [15], [19]–[24], [27], [32]. However, most molecular and morphological studies focused on higher-level avian relationships are consistent in placing members of the Pelecaniformes (exclusive of the tropicbirds) as part of a larger “waterbird” clade that includes the shoebill, hammerkop, storks, ibises, herons, tube-nosed seabirds, penguins, and loons [4], [24], [27], [29].

The monophyly of a ‘core’ assemblage of pelecaniforms (“Suloidea” *sensu* Cracraft [3]), including the sulids, darters, and cormorants, is consistently well supported in phylogenetic studies. The sister taxon to Suloidea is not entirely clear, however, as most recent molecular studies recover *Fregata* in this position [24], [27]–[29], while morphological data typically recovers *Pelecanus* as the sister taxon to Suloidea, and *Fregata* as sister taxon to this larger clade [3], [4], [25], [26]. Noteably, Mayr ([23]: Figure 1) did recover Suloidea as more closely related to *Fregata* than to *Pelecanus*, though this result also involved a sister-taxon relationship between Suloidea and a wing-propelled diving clade of Spheniscidae and the extinct pelecaniform family Plotopteridae. Relationships among the major families of the ‘core’ pelecaniforms have also proven difficult to resolve, with some mitochondrial datasets suggesting an unconventional sister taxon relationship between Anhingidae and Sulidae [2], [17], while analyses of nuclear genes [27], [29] and morphological data [4], [23], [25], [26] typically result in strong support for a more traditional Anhingidae + Phalacrocoracidae clade. Long-branch attraction of mitochondrial sequences has previously been demonstrated as a contributing factor to this problem [17]. Furthermore, lower-level relationships inferred within Suloidea exhibit varying degrees of congruence and conflict between molecular and morphological datasets. Species relationships within the Sulidae are largely congruent between both data types, with the exception of the position of Abbott's booby, *Papasula abbotti* [6], [7], while relationships within the Phalacrocoracidae are predominantly incongruent [16], [33], [34].

Contrary to the typical lament of the poor quality of the avian fossil record, the Pelecaniformes are represented by extensive fossil material, with the oldest reliable records dating to the early Eocene [35]–[38]. The pelecaniform fossil record has variably included bizarre extinct taxa such as the giant bony-toothed Pelagornithidae [39], the enigmatic *Protoplotus beauforti* [40]–[42], and the wing-propelled diving Plotopteridae [43]. Pelagornithidae have traditionally been considered as closely related to the Pelecaniformes or the Procellariiformes, though their exact relationships within either order have not been clear [36], [44]–[46]. However, new fossil taxa, re-evaluation of previously collected specimens, and recent phylogenetic analyses suggest that the Pelagornithidae may belong outside of Neoaves, possibly as the sister taxon to Anseriformes [25], [46]–[48]. The Plotopteridae have previously been considered as closely related to, or within, Suloidea [36], [49], [50] with affinities to Anhingidae often proposed [5], [36]. Mayr [23], raised the possibility that plotopterids may actually be related to penguins, and recovered a monophyletic clade of Spheniscidae + Plotopteridae that also nested within Steganopodes as the sister taxon to Suloidea. However, Mayr [23] did not test the monophyly of Plotopteridae, instead coding this taxon as a composite OTU based on the descriptive literature for several taxa (see [23]: p. 62). The phylogenetic placement of Plotopteridae clearly has implications for waterbird and pelecaniform phylogeny, as well as for patterns of morphological character and life history evolution, particularly in relation to the evolution of wing-propelled diving.

Extensive records of stem and crown members of most extant pelecaniform families also exist [37], [38]. The Sulidae in particular have a diverse fossil record, with over 20 named fossil species, and possibly more than 40 distinct species [36], [38]. However, the oldest definitive stem member of the Sulidae is unclear [36], [51], [52]. In contrast, the fossil record of frigatebirds is depauparate, with the notable exception of the early Eocene taxon *Limnofregata* [35]. *Limnofregata* is noteworthy, as it represents the only pre-Quaternary record of stem Fregatidae, and is considered the oldest reliable fossil record of Pelecaniformes [36]. Recently, several new fossils have been described that may represent the most ancient stem members of other pelecaniform families, including *Lithoptila abdounensis*, a stem member of the tropicbird lineage from the upper Paleocene of Morocco [26], [53], and ?*Borvocarbo stoeffelensis*, a small, cormorant-like bird from the late Oligocene of Germany that may be a stem member of Phalacrocoracidae or Phalacrocoracoidea ( =  Phalacrocoracidae + Anhingidae) [54], [55].

Herein, I assess the monophyly and phylogenetic relationships of the Pelecaniformes through the analysis of a morphological phylogenetic dataset of waterbirds encompassing 59 taxa and 464 characters. I also assess the monophyly and relationships of the extinct pelecaniform family Plotopteridae. Additionally, I provide tests of the relationships of several other fossil pelecaniforms, including *Limnofregata*, *Prophaethon*, *Lithoptila*, and ?*Borvocarbo stoeffelensis*. The antiquity of these taxa, coupled with their purported status as stem members of several extant pelecaniform families, makes them particularly interesting for studies of the divergence times of higher-level avian clades. Indeed, several of these taxa have been utilized as fossil calibration points in recent molecular clock analyses of higher-level avian diversification [21], [27], [28]. However, the phylogenetic relationships of many of these taxa (e.g., *Limnofregata*, ?*Borvocarbo stoeffelensis*, several plotopterids) have never been rigorously tested in the context of a modern cladistic analysis of morphological character data, and several have only been included in phylogenetic analyses of more limited taxonomic scope (e.g., *Prophaethon*, *Lithoptila*, several plotopterids). Thus, several of these taxa fail to meet the criteria for effective fossil calibration points outlined by previous authors [56], [57]. The veracity of the referral of these fossil taxa to pelecaniform families is of particular concern, as the monophyly and higher-level relationships of the Pelecaniformes have remained extremely controversial [3], [4], [15], [20], [22]–[25], [27]–[29]. The influence that discrepancies in waterbird topologies might have on the phylogenetic placement of these fossil pelecaniforms, and thus the relative impact of their use as fossil calibrations, has not been investigated.

## Methods

### Institutional Abbreviations

**BMS**, Buffalo Museum of Science, Buffalo, New York; **FMNH**, The Field Museum of Natural History, Chicago, Illinois; **GMNH**, Gunma Museum of Natural History, Tomioka, Gunma Prefecture, Japan; **KMNH**, Kitakyushu Museum and Institute of Natural History, Kitakyushu, Japan; **MACN**, Museo Argentino de Ciencias Naturales, Buenos Aires, Argentina; **NSMT**, National Science Museum, Tokyo, Japan; **UCMP**, University of California Museum of Paleontology; **USNM**, National Museum of Natural History, Smithsonian Institution, Washington, D.C.; **UWBM**, Burke Museum of Natural History and Culture, Seattle, Washington; **UWGM**, University of Wyoming Geological Museum, Laramie, Wyoming; **WSGS**, Wyoming State Geological Survey, Laramie, Wyoming.

### Taxon Sampling

Though recent molecular and morphological analyses [4], [26], [27], [29] of higher-level avian phylogeny generally agree on the taxonomic content of the waterbird clade (with several noteable exceptions such as *Phoenicopterus*, *Podiceps*, and *Phaethon*), the fact that: **1)** relationships within the waterbird tree are so contentious, **2)** Pelecaniformes may not be monophyletic, and **3)** fossil pelecaniforms may not actually be closely related to their purported extant pelecaniform families; necessitated a broad taxonomic sampling scheme that included diverse members from throughout the waterbird clade. Accordingly, 57 waterbird taxa were included in the analysis, with the following families represented: Gaviidae, Podicipedidae, Spheniscidae, Procellariidae, Diomedeidae, Hydrobatidae, Pelecanoididae, Phoenicopteridae, Ciconiidae, Ardeidae, Threskiornithidae, Balaenicipitidae, Scopidae, Phaethontidae, Pelecanidae, Fregatidae, Sulidae, Anhingidae, and Phalacrocoracidae (Appendix S1). *Eudromia elegans* (Tinamidae) and *Gallus gallus* (Phasianidae) were utilized as outgroups to root phylogenetic trees.

An important caveat is that the taxonomic scope of the present analysis is limited to the waterbird clade. Thus, the current dataset is not designed to test the *global* relationships of any included taxa within Aves. This is not a major problem for most included taxa, whose status as a member of the waterbird clade is uncontroversial. However, for taxa such as *Phaethon*, *Podiceps*, and *Phoenicopterus*, which have been recovered in previous phylogenetic studies outside of the waterbird clade, often as closely related members of a clade variably termed ‘Metaves’ [19], [27], [29], the issue of taxonomic scope is more of a concern. Accordingly, it is most appropriate to view the current analysis as a rigorous test of the relationships of these taxa *if they are indeed waterbirds*, and as uninformative of their relationships if they belong outside of the waterbird clade.

*Limnofregata azygosternon* was first described by Olson [35] as a member of the pelecaniform family Fregatidae, and a possible direct ancestor of modern *Fregata*. The holotype (USNM 22753), and all subsequently referred specimens with the exception of one (USNM 447002), were collected from the early Eocene Fossil Butte Member of the Green River Formation, which radiometric dating indicates is 51.97+/−0.16 Myr [58]. In 2005, a second species, *Limnofregata hasegawai*, was described, along with additional new material of *L. azygosternon* [59]. The new species is virtually identical to *L. azygosternon*, and differs from it only in its overall larger size, and proportionally longer rostrum [59]. As the two species of *Limnofregata* currently recognized [59] differ only in relative size and proportion of the rostrum, and not in any discrete anatomical characters, observations based on specimens from both *L. azygosternon* and *L. hasegawai* were lumped, and *Limnofregata* was coded as a single OTU in this analysis. Morphological characters were coded for *Limnofregata* based on first-hand examination of the majority of specimens, and were supplemented by published descriptions [35], [59] where necessary. In total, *Limnofregata* could be scored for 251 characters (54.1%) in the dataset.

To assess the monophyly and relationships of the Plotopteridae, four previously recognized members of this extinct family were included: *Plotopterum joaquinensis* (USNM 8927–cast of LACM 8927; [43]), *Phocavis maritimus* [60], *Tonsala hildegardae* (USNM 256518; [50]) and *Copepteryx hexeris* (Holotype: USNM 486682–cast of KMNH VP 200,006; Paratypes: USNM 243773–cast of KMNH VP 200,001; USNM 486684–cast of KMNH VP 200,002; USNM 243774–cast of NSMT VP 15035; [5]). Of the plotopterids included, only *Phocavis* was coded strictly from the literature [60]. Despite its fragmentary nature (known only from a tarsometatarsus) the inclusion of *Phocavis* in the current analysis is worthwhile, as this taxon has been described as the oldest, and possibly most basal member of Plotopteridae [5], [60], though Mayr [23] noted its overall similarity to *Limnofregata* and also raised the possibility that *Phocavis* represents the sister taxon to a Plotopteridae + Spheniscidae clade. The plotopterids included could be scored for the following proportions of characters in the dataset: *Plotopterum joachinenesis*, 3.1%; *Phocavis maritimus*, 7.5%; *Tonsala hildegardae*, 17.2%; *Copepteryx hexeris*, 44.2%. In addition to these four plotopterids, several other specimens of Plotopteridae were referred to for comparative purposes: *Copepteryx titan* (USNM 486685–cast of KMNH VP 200,004), *Tonsala*? sp. (USNM 243775–cast of KMNH VP 200,003).

In addition to *Limnofregata* and Plotopteridae, three other taxa purported to be stem members of extant pelecaniform families (or more inclusive clades) were included in the analysis. In constrast to *Limnofregata* and Plotopteridae, these taxa were coded exclusively from the primary and descriptive literature. Both *Prophaethon shrubsolei* from the lower Eocene London Clay of England [61], [62] and *Lithoptila abdounensis* from the upper Paleocene of Morocco [26], [53] are members of the extinct family Prophaethontidae, which has been recovered as the sister taxon to the extant Phaethontidae [26]. ?*Borvocarbo stoeffelensis*, a small, cormorant-like bird from the late Oligocene of Germany was recently described by Mayr [55], and includes a referred isolated foot that previously had been tentatively assigned to the extinct genus *Oligocorax*. Mayr [54], [63] noted the similarities between ?*Borvocarbo stoeffelensis* and extant ‘microcormorants’, but cautioned against referral of ?*Borvocarbo stoeffelensis* to crown or stem Phalacrocoracidae, noting that the species exhibits several plesiomorphies of Phalacrocoracoidea ( =  Anhingidae + Phalacrocoracidae). These three fossil taxa could be scored for the following proportion of characters in the dataset: *Prophaethon shrubsolei*, 20.5%; *Lithoptila abdounensis*, 24.4%; ?*Borvocarbo stoeffelensis*, 7.3%.

### Character Sampling and ILD Tests

A total of 464 osteological characters were scored for each taxon (Appendix S2, Appendix S3). Characters can be divided into coarsely defined anatomical regions as follows: cranial skeleton, 95; axial skeleton, 11; pectoral skeleton, 188; pelvic skeleton, 169; miscellaneous, 1. 88 (19%) characters are new or have been formulated for phylogenetic analysis for the first time. The remaining characters, or some variation thereof, have been utilized previously in phylogenetic analyses. Characters were assembled from a variety of studies, with the primary sources being [3], [7], [23], [25], [26], [33], [64]. With regard to previously utilized characters, in general these were sampled according to the following criteria: **1)** character states had to vary within the ingroup; **2)** characters unique to only a single terminal taxon in the ingroup were not included; **3)** characters and individual character states were independent of each other; **4)** homology of the character and character states across the ingroup was clear and relatively uncontroversial; and **5)** distinctions between character states were well-defined. Morphological traits were coded into binary or multistate characters. In cases where homology with a particular state in a taxon or set of taxa could no be confidently hypothesized for a character, these taxa were coded as inapplicable ( =  “−”) for that charcter. In the context of a maximum parsimony analysis this is effectively the same as treating these taxa as missing data ( =  “?”) for that charcter. Inapplicable characters were most problematic for the two outgroup taxa included, given their morphological dissimilarity to many members of the ingroup. However, only 16 of the 464 of the included characters, or 3.4%, required inapplicable codings in one or both outgroup taxa.

Four incongruence length difference (ILD) tests [65], were performed on the three primary anatomical partitions using the partition homogeneity test implemented in PAUP* 4.0b10 [66]. These analyses were performed with the fossil taxa excluded from the dataset, as several fossil taxa cannot be scored for characters in one or more anatomical partition (e.g., all included plotopterids lack cranial material), or can only be scored for a limited amount of characters (e.g., *Prophaethon* and *Lithoptila* are missing data for most pectoral characters). The first test compared all three major partitions (cranial, pectoral, pelvic) at the same time, utilizing heuristic searches of 500 pseudoreplicates with 25 random addition sequence replicates per pseudoreplicate. The remaining ILD tests were pairwise comparisons of anatomical partititions (i.e., cranial vs. pectoral, cranial vs. pelvic, pectoral vs. pelvic) and utilized heuristic searches of 200 pseudoreplicates with 20 random addition sequence replicates per pseudoreplicate.

### Phylogenetic Analyses

Phylogenetic analyses of the taxon-character matrix were performed in PAUP* 4.0b10 [66]. Characters were equally weighted and treated as unordered. A heuristic search was performed with 10,000 random addition sequence replicates to obtain the most parsimonious trees for the dataset. Tree bisection and reconnection (TBR) was utilized as the branch-swapping algorithm for the heuristic search. Zero length branches were collapsed if they lacked support under any of the most parsimonious reconstructions, following ‘rule 1’ of Coddington and Scharff [67]. Clade support was quantified through bootstrap analysis [68]. Heuristic searches were performed on 2,000 pseudoreplicate datasets, with 10 random addition sequence replicates for each bootstrap search. The maximum number of trees saved for each random addition sequence replicate was set to 100 to prevent searches from becoming stuck on a large island of MPTs during any particular random addition sequence replicate. Though this search strategy reduces the amount of tree space explored for any given random addition sequence replicate, it allows for a much larger number of bootstrap replicates to be performed. Bremer support values were also calculated for each node in the strict consensus of all MPTs using TreeRot.v2c [69].

In addition to this analysis of the full dataset, a phylogenetic analysis was also performed with the eight fossil taxa removed. Three additional parsiomony analyses were performed analyzing each of the three major anatomical partitions (cranial, pectoral, pelvic). These analyses were also performed with the eight fossil taxa removed, as many of the fossil taxa included cannot be scored for characters in some partitions (e.g., plotopterids and cranial characters), or can only be scored for a small amount of characters (e.g., *Prophaethon*/*Lithoptila* and pectoral characters). These parsimony analyses were performed following the same protocol and methods described for the full dataset analysis listed above. Clade support was assessed using bootstrap analyses and Bremer support analyses as described above.

Several constraint analyses were also performed on the full dataset. Two of these assessed the relative support for recent higher-level phylogenenetic relationships of waterbirds [4], [29]. These two analyses involved setting up a backbone constraint tree that matched the topology recovered by either: **1)** Livezey and Zusi's [4] morphological anlaysis, or **2)** Hackett et al.'s [29] molecular analysis (Figure 1). Four additional analyses were performed that focus on the relationships of one or more members of the Pelecaniformes that have been contentious (see Introduction above). These included analyses that constrained: **1)** the monophyly of a traditional Pelecaniformes; **2)** the monophyly of a Plotopteridae + Spheniscidae clade; **3)** the monophyly of a *Balaeniceps* + *Scopus* + *Pelecanus* clade; and **4)** the monophyly of an Anhingidae + Sulidae clade. These constraint analyses were performed following the same protocol described for the primary phylogenetic analysis of the full dataset above.

At present, there are essentially no methods for assessing whether topologies alternative to the optimal tree/s are statistically significant worse fits to the character data in a parsimony framework. Non-parametric paired sites tests such as the Templeton test (a variation on the Wilcoxon signed ranks test; [70], [71]), the winning-sites test [72], and the Kishino and Hasegawa, or KH test [73] have been used extensively by morphological systematists, primarily because they can be implemented in a parsimony framework, and are included in popular phylogenetics software (e.g., PAUP* 4.0b10 [66]). However, all of these tests assume a null hypothesis where the expected difference in optimality score between alternative phylogenies is zero [74]–[76]. This requires that the topologies being compared must be specified *a priori*, and without reference to the data being used for the test. However, *nearly all* uses of these tests involve comparing alternative topologies to the optimal topology estimated from the data. This application guarantees that the null expectation of difference will always be larger than zero, and violates any assumption of a normal distribution of differences in optimality scores between topologies [76]. More recently, non-parametric tests, such as the Shimodaira-Hasegawa, or SH test [75] and the Approximately Unbiased, or AU test [77]; and parametric tests, such as the SOWH test [75], [76], have been developed that explicitly avoid these shortcomings. However, there is currently no implementation available for these tests using morphological data in a parsimony framework.

As noted by Goldman et al. [76], there is one possible modification of the KH test that allows for much more limited, but statistically valid, interpretation of its results in the context of the SH test. The *P*-value that would be obtained under the SH test is necessarily greater than or equal to half the *P*-value obtained by the KH test [76]. Thus, if the adjusted *p*/2 value from a KH test is greater than 0.05 (i.e., for a 5% significance level), which would indicate the inability to reject an alternate topology, than the *P*-value from the SH test would necessarily give the same conclusion [76]. However, in all cases where a KH test would indicate rejction of the alternative topology, (i.e., where KH test *p*/2<0.025), it is impossible to know whether the SH test would, or would not, indicate rejection of the alternate topology at a 5% significance leve (i.e., the SH test *P*-value will exceed the KH test *p*/2 value by an unknown amount) [76]. This severly limits the informativeness of the KH test, and essentially renders it an asymmetrical test of alternative topologies. If the KH test *p*/2>0.05, it can be concluded (on the basis of the SH test), that the present dataset cannot significantly reject the alternative topology as an equally good approximation of the phylogeny. Note however, that an additional confounding factor is that these statistical tests do not make corrections for assessing multiple trees, nor is a simple multiple-test correction such as the Bonferroni correction applicable to the problem [78].

KH tests were performed in PAUP* 4.0b10 [66] to assess differences between most parsimonious trees resulting from the unconstrained analysis of the full dataset, and most parsimonious trees obtained under the six constraint analyses outlined above. *P*-values were halved and interpreted following the recommendations of Goldman et al. [76]. Winning-sites and Templeton tests were also computed in PAUP* 4.0b10 [66], purely for comparison with the results of the modified KH tests, bearing in mind the statistical invalidity of these tests as outlined above.

## Results

### Higher-level Phylogeny of Waterbirds

**Figure 2:**
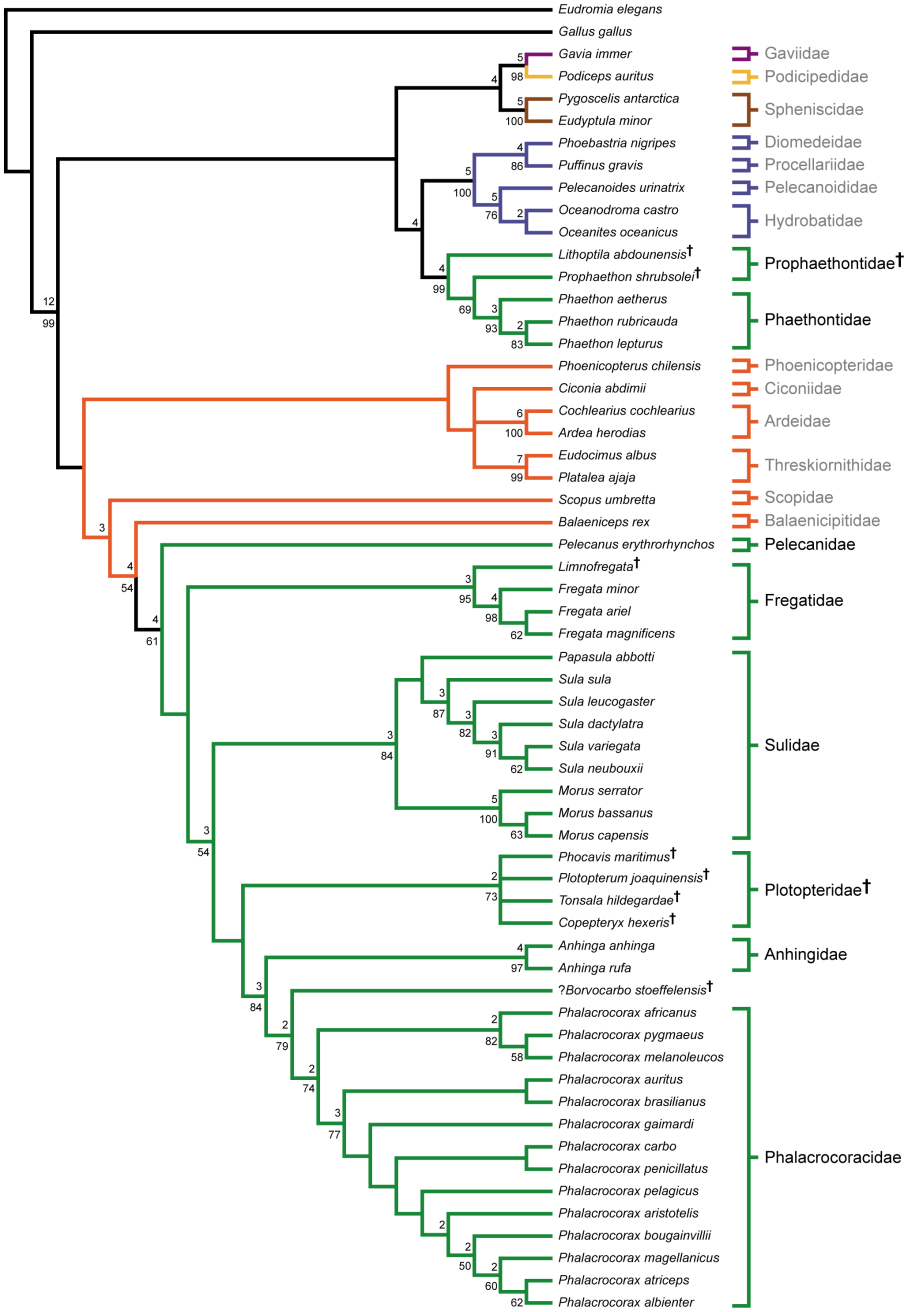
Strict consensus of 6 MPTs from the full dataset analysis. Tree length: 1222, C.I.: 0.441, R.I.: 0.852. Bootstrap proportions greater than 50% are shown below nodes. Bremer decay values greater than one are shown above nodes. Fossil taxa are indicated with a “**†**” superscript after taxon name. Branch colors are as in Figure 1. Waterbird families are indicated to the right of taxa, with members of a traditional Pelecaniformes indicated in black, and all others in grey.

Phylogenetic analysis of the full dataset resulted in the recovery of six most parsimonious trees (MPTs), the strict consensus of which is presented in Figure 2. Individual MPTs were 1222 steps, with consistency and retention indices of 0.441 and 0.852, respectively. MPTs differed only in the relationships among the four plotopterids, and the relationship of Ciconia relative to Ardeidae and Threskiornithidae. The monophyly of individual waterbird families (where more than one member was included in the analysis) are well supported in most cases (e.g., Spheniscidae, Phaethontidae, Ardeidae, Threskiornithidae, Fregatidae, Sulidae, Anhingidae, Phalacrocoracidae).

A monophyletic Pelecaniformes is not recovered, with Phaethontidae (tropicbirds) and the extinct Prophaethontidae comprising the sister taxon to Procellariiformes as part of a larger group including loons, grebes, and penguins (Figure 2). All other pelecaniforms are recovered in a monophyletic Steganopodes [79] (i.e., pelicans, frigatebirds, sulids, anhingas, cormorants). A sister taxon relationship between Anhingidae and Phalacrocoracidae is strongly supported, and this clade is recovered as the sister taxon to the extinct Plotopteridae. This Phalacrocoracoidea + Plotopteridae clade is recovered as the sister taxon to Sulidae in a monophyletic Suloidea. Fregatidae and Pelecanidae form successive sister taxa to Suloidea in a monophyletic Steganopodes (Figure 2). Balaeniceps and Scopus are recovered as successive sister taxa to Stegnopodes.

A ‘reduced’ Ciconiiformes clade (Ciconiimorphae *sensu* Livezey and Zusi [4]) is recovered as the sister taxon to the *Scopus* + *Balaeniceps* + Steganopodes clade. Monophyly of this ‘reduced’ Ciconiiformes clade is not strongly supported in the present analysis (Figure 2). The monophyly of both Ardeidae and Threskiornithidae is strongly supported, though their relationships to each other and to *Ciconia* are not clear. In three of the MPTs Ardeidae and Threskiornithidae are sister taxa, and *Ciconia* is recovered as the sister taxon to this larger clade. In the other three MPTs, *Ciconia* is recovered as the sister taxon to Threskiornithidae, and this larger clade is sister taxon to Ardeidae.

A large, basally diverging clade including loons, grebes, penguins, procellariforms and tropicbirds is recovered, similar to Livezey and Zusi's [4] Subdivision Pygopodo-tubinares, with the exception of the inclusion of tropicbirds (Figure 2). The monophyly of this larger clade is not particularly well supported, however, and none of the three basal-most divergences in this clade are supported by bootstrap values greater than 50% (Figure 2). As noted above, Phaethontidae and the extinct Prophaethontidae are recovered as the sister taxon to Procellariiformes. Penguins are recovered as the sister taxon to a well supported loon/grebe clade, and this larger clade forms the sister taxon to the procellariform/tropicbird clade (Figure 2).

### Extant Taxa Only Analysis

**Figure 3 pone-0013354-g003:**
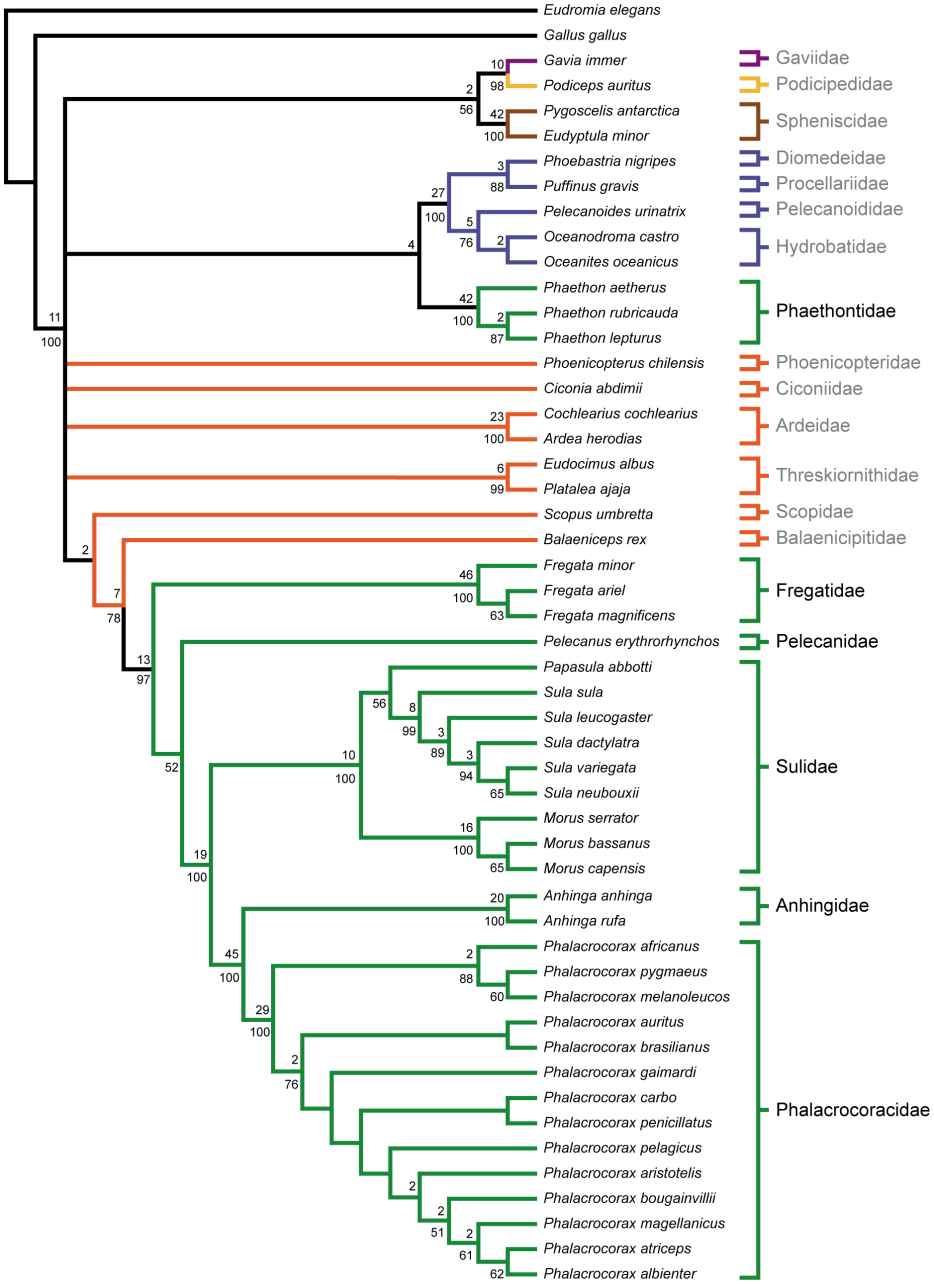
Strict consensus of 7 MPTs from the analysis of extant taxa only. Tree length: 1154, C.I.: 0.461, R.I.: 0.860. Bootstrap proportions greater than 50% are shown below nodes. Bremer decay values greater than one are shown above nodes. Branch colors are as in Figure 1. Waterbird families are indicated to the right of taxa, with members of a traditional Pelecaniformes indicated in black, and all others in grey.

Phylogenetic analysis of the extant taxa only dataset resulted in the recovery of seven MPTs (Figure 3). Individual most parsimonious trees were 1154 steps, with consistency and retention indices of 0.461 and 0.860, respectively. The MPTs are nearly identical to those recovered in analysis of the full dataset, with several notable exceptions. First, there is not unequivocal support for the monophyly of a large clade including loons, grebes, penguins, procellariforms, and tropicbirds. The monophyly of this clade is recovered in only six out of the seven MPTs, though two monophyletic subclades (one consisting of loons, grebes and penguins, and one consisting of procellariforms and tropicbirds) are recovered in all MPTs. In one MPT, the procellariforms + tropicbirds subclade and the loons + grebes + penguins subclade are recovered as successive sister taxa to all other ingroup taxa. Second, the large ‘reduced’ Ciconiiformes clade from the full dataset analysis is not recovered as monophyletic in all MPTs. In two MPTs, herons, threskiornithids, *Ciconia*, and *Phoenicopterus* are recovered as four separate lineages forming successive sister taxa to the *Scopus* + *Balaeniceps* + Steganopodes clade. In one MPT, a monophyletic clade of herons, threskiornithids, and *Ciconia* is recovered as the sister taxa to the *Scopus* + *Balaeniceps* + Steganopodes clade, and *Phoenicopterus* is recovered more basally as the sister taxon to this more inclusive group. Finally, in a single MPT, *Phoenicopterus* is recovered as the sister taxon to a large clade including loons, grebes, penguins, Procellariiformes, and tropicbirds. The positions of *Fregata* and *Pelecanus* have also switched relative to the MPTs from the full dataset analysis. In the extant taxa only analysis, *Pelecanus* is recovered as the sister taxon to Suloidea, with *Fregata* as the sister taxon to this larger clade in a monophyletic Steganopodes.

### ILD Tests and Partition Analyses

**Figure 4 pone-0013354-g004:**
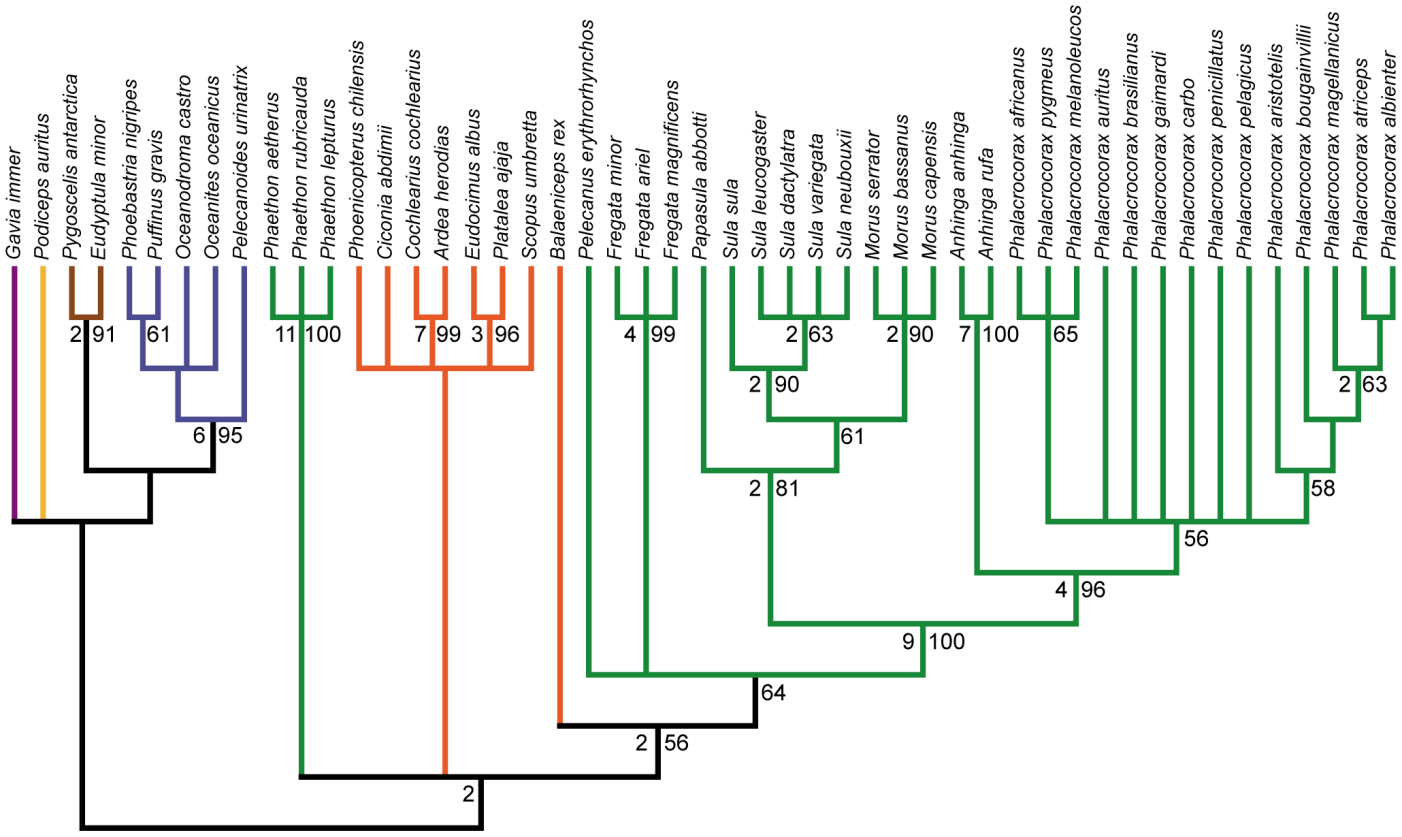
Strict consensus of 61 MPTs from the cranial partition analysis. Tree length: 211, C.I.: 0.493, R.I.: 0.879. Bootstrap proportions greater than 50% are shown to the right of nodes. Bremer decay values greater than one are shown to the left of nodes. Branch colors are as in Figure 1.

The incongruence length difference test comparing the three major anatomical regions (cranial, pectoral, pelvic) recovered a significant difference in phylogenetic signal between partitions (*p* = 0.002). The pairwise ILD tests suggest that this incongruence may primarily be between the pectoral and pelvic anatomical partitions, which was the only one of the three pairwise ILD tests that recovered significant incongruence (*p* = 0.005; cranial vs. pectoral *p* = 0.115; cranial vs. pelvic *p* = 0.205). The strict consenses of most parsimonious trees resulting from each of the partitioned analyses are presented in Figures 4–6.

**Figure 5 pone-0013354-g005:**
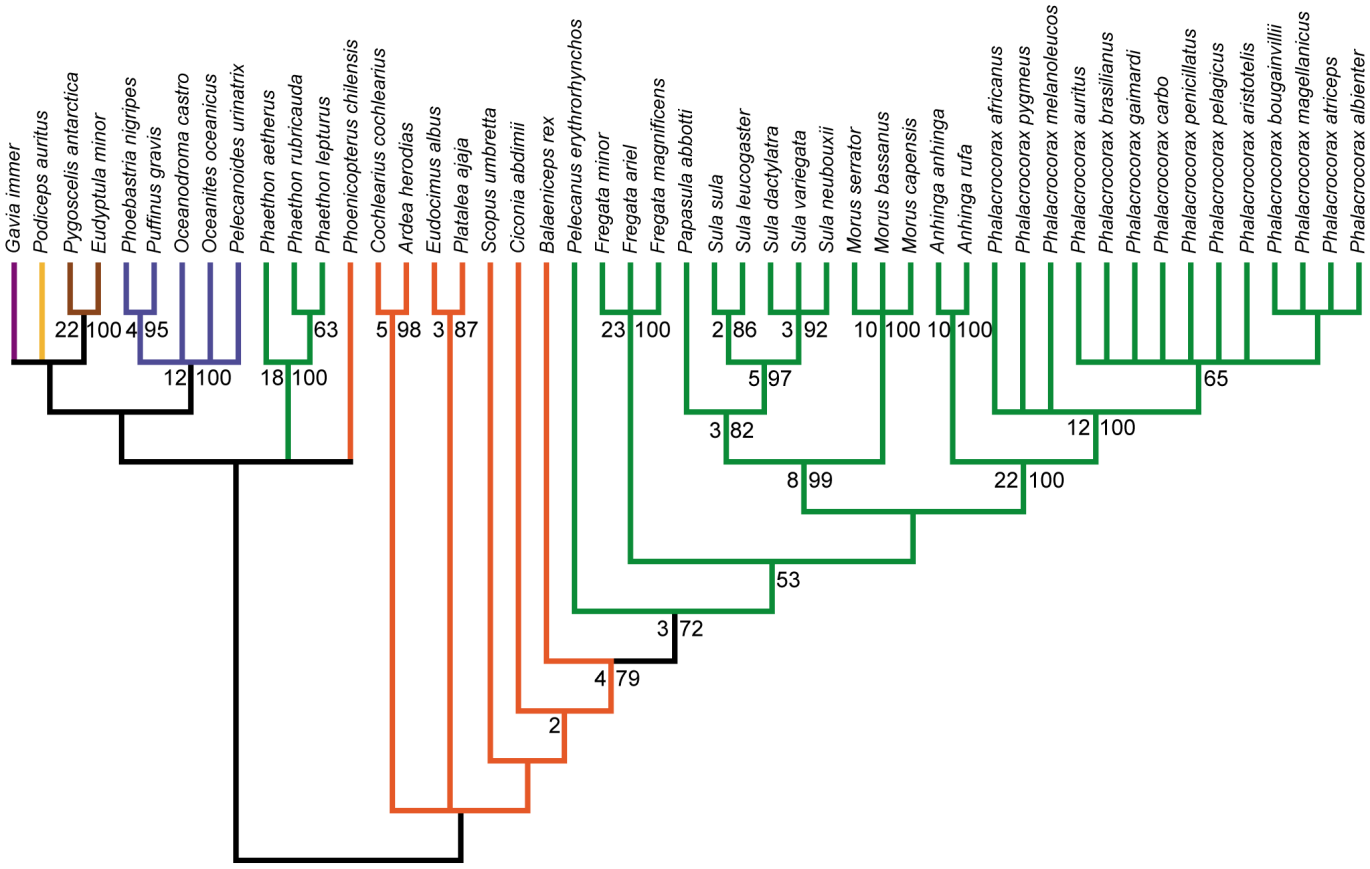
Strict consensus of 7 MPTs from the pectoral partition analysis. Tree length: 470, C.I.: 0.468, R.I.: 0.860. Bootstrap proportions greater than 50% are shown to the right of nodes. Bremer decay values greater than one are shown to the left of nodes. Branch colors are as in Figure 1.

**Figure 6:**
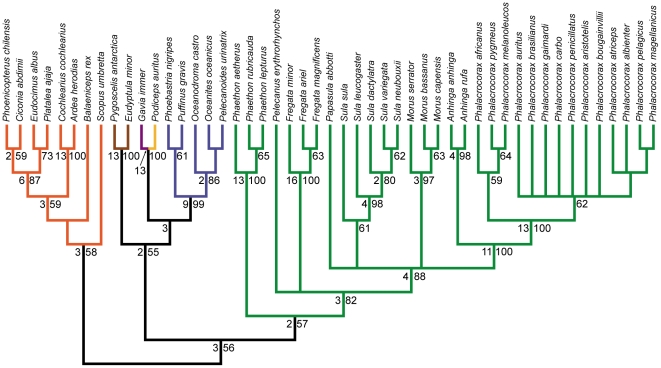
Strict consensus of 22 MPTs from the pelvic partition analysis. Tree length: 388, C.I.: 0.503, R.I.: 0.879. Bootstrap proportions greater than 50% are shown to the right of nodes. Bremer decay values greater than one are shown to the left of nodes. Branch colors are as in Figure 1.

Relationships within Steganopodes are relatively consistent between the partitioned analyses (Figures 4–6). A notable exception is the relative position of *Papasula*, which is recovered as the sister taxon to *Sula* in the pectoral partition analysis (as in the full dataset and extant taxa only analyses), and as the sister taxon to all other Sulidae in the cranial partition analysis. An additional interesting result is that neither the cranial nor pelvic partitioned analyses unambiguously resolve the relative positions of *Fregata* and *Pelecanus* to Suloidea, though the pectoral partition does recover a sister taxon relationship between *Fregata* and Suloidea. In light of the results of the extant taxa only analyses, which recover a sister clade relationship between *Pelecanus* and Suloidea, this suggests that the phylogenetic signal supporting a *Pelecanus* + Suloidea clade is cumulative across the three major anatomical partitions [80], as none of the remaining 12 characters (i.e., those not included in the partitioned analyses) provide unambiguous support for a closer relative relationship of *Fregata* or *Pelecanus* to Suloidea.

Relationships of the members of the ‘reduced’ Ciconiiformes clade differ considerably between the three partitioned analyses (Figures 4 – 6). In the MPTs from the cranial partition analysis, these taxa are recovered in a weakly supported (bootstrap support <50%) monophyletic clade, which also includes *Scopus* (Figure 4). In contrast, the MPTs from the pectoral partition analysis recover a sister taxon relationship between *Ciconia* and a *Scopus* + *Balaeniceps* + Steganopodes clade, with Threskiornithidae and Ardeidae in an unresolved polytomy basal to this group. *Phoenicopterus* is recovered as a member of the large basal clade including loons, grebes, penguins, procellariforms, and tropicbirds, though none of these splits are supported by bootstrap values greater than 50% (Figure 5). Finally, in the pelvic partition analysis, a monophyletic group consisting of the ‘reduced’ Ciconiiformes taxa is recovered, and *Phoenicopterus* and *Ciconia* are resolved as sister taxa closely related to Threskiornthidae (Figure 6). Additionally, *Scopus* and *Balaeniceps* are recovered as successive sister taxa to this ‘reduced’ Ciconiiformes clade, and this larger group is resolved as being one of the basal two phylogenetic splits in the ingroup (Figure 6).

The relative relationships of members of the large basal clade including loons, grebes, penguins, procellariforms, and tropicbirds (Figures 2, 3), also differ between the partitioned analyses (Figures 4–6). In the MPTs from the cranial partition analysis, this group is not monophyletic, with tropicbirds recovered as more closely related to the other ingroup taxa. A monophyletic clade including loons, grebes, penguins, and procellariforms is still recovered as one of the two basal splits in the ingroup, though within this group loons and grebes are not unambiguously monophyletic, and a sister taxon relationship between penguins and procellariforms is weakly supported (Figure 4). The MPTs of the pectoral partition analysis bear the most resemblance to the results of the full dataset and extant taxa only analyses (Figure 5). In these a large, basally splitting clade including loons, grebes, penguins, procellariforms, and tropicbirds is recovered, though *Phoenicopterus* is also recovered in this group. A clade including loons, grebes and penguins is weakly supported, as well as a sister taxon relationship between this group and Procellariiformes. In the pelvic partition analysis, tropicbirds are recovered as sister taxon to Steganopodes in a traditional Pelecaniformes (Figure 6). A monophyletic clade including loons, grebes, penguins and procellariforms is recovered as the sister taxon to Pelecaniformes, with an expanded Ciconiiformes clade more distantly related, as noted above. The monophyly of loons and grebes is strongly supported in the pelvic partition analysis, and this clade is weakly supported as the sister taxon to Procellariiformes. Penguins form the sister clade to this larger group (Figure 5).

### Constraint Analyses

The constraint analysis enforcing the higher-level waterbird topology recovered by Livezey and Zusi's [4] morphological anlaysis resulted in the recovery of three MPTs, each of which were 1243 steps (21 steps longer than the unconstrained MPTs), with consistency and retention indices of 0.434 and 0.847, respectively. The constraint analysis enforcing the higher-level waterbird topology recovered by Hackett et al.'s [29] molecular analysis recovered three MPTs, each of which were 1371 steps (149 steps longer than the unconstrained MPTs), with consistency and retention indices of 0.393 and 0.820, respectively. The sister taxa relationships of the unconstrained fossil taxa in the MPTs of the constraint analyses are not different from in the unconstrained analyses. ?*Borvocarbo stoeffelensis* is still recovered as the sister taxon to *Phalacrocorax*; Plotopteridae is still recovered as the monophyletic sister taxon to Phalacrocoracoidea, *Limnofregata* is still recovered as the sister taxon to *Fregata*, and *Prophaethon* and *Lithoptila* are still recovered as successive sister taxa to *Phaethon*.

Constraint analyses enforcing a monophyletic traditional Pelecaniformes resulted in the recovery of six MPTs, (C.I. 0.438; R.I. 0.850). These MPTs are only nine steps longer than the MPTs of the unconstrained full dataset analysis (1231 vs. 1222 steps). As above, the relative sister taxon relationships of the fossil taxa in this constraint analyses are the same as in the full dataset analysis. In the MPTs, a *Limnofregata* + *Fregata* clade is recovered as sister taxon to Suloidea, with *Pelecanus* forming the sister taxon to this larger group. A monophyletic Phaethontidae + Prophaethontidae clade is recovered as the sister taxon to Steganopodes. *Balaeniceps* and *Scopus* are recovered as successive sister taxa to Pelecaniformes. Interestingly, enforcing a monophyletic Pelecaniformes also results in *Phoenicopterus* being recovered as the sister taxon to the large basal clade including loons, grebes, penguins, and procellariforms. Within this group, penguins and procellariforms are recovered as sister taxa, and this group is sister taxon to a monophyletic loon + grebe clade. The remaining members of the ‘reduced’ Ciconiiformes clade from the full dataset analysis are recovered in a monophyletic clade, with *Ciconia* forming the sister taxon to Threskiornithidae.

Constraint analyses enforcing the monophyly of a Plotopteridae + Spheniscidae clade resulted in the recovery of 12 MPTs (C.I. 0.440; R.I. 0.851). These MPTs are only four steps longer than the MPTs of the unconstrained full dataset analysis (1226 vs. 1222 steps). In the MPTs, a monophyletic Plotopteridae + Spheniscidae clade is recovered as the sister taxon to a clade including loons and grebes. The remaining relationships in the MPTs are nearly identical to those recovered in the full dataset analysis, with two exceptions. First, the relative relationships of the *Limnofregata*/*Fregata* clade and *Pelecanus* to each other are not resolved. Second, two alternate placements of *Papasula*: as the sister taxon to *Sula*, or as the sister taxon to all other sulids; are equally parsimonious.

Enforcing a monophyletic clade consisting of *Balaeniceps*, *Scopus*, and *Pelecanus* results in the recovery of 28 MPTs (C.I. 0.432; R.I. 0.846). These MPTs are 25 steps longer than the MPTs from the unconstrained full dataset analysis (1247 vs. 1222 steps). As above, the relative sister taxon relationships of the fossil taxa in this constraint analysis are the same as in the full dataset analysis. Much of the higher-level relationships outside of the *Fregata* + Suloidea clade are not resolved, with a basal polytomy including: a monophyletic clade of loons, grebes and penguins; *Phoenicopterus*; Ardeidae; Threskiornithidae; *Ciconia*; Procellariiformes; a monophyletic clade of *Scopus*, *Balaeniceps* and *Pelecanus*; and a monophyletic clade of Prophaethontidae and *Phaethon*. An Adams consensus of the 28 MPTs reveals that much of this poor resolution can be attributed to the uncertain phylogenetic placement of *Phoenicopterus*.

Constraints analyses enforcing the monophyly of an Anhingidae + Sulidae clade resulted in the recovery of 23 MPTs (C.I. 0.427; R.I. 0.843). MPTs are 39 steps longer than those from the unconstrained full dataset analysis (1261 vs. 1222 steps). Relationships recovered are virtually identical to those present in the MPTs from the unconstrained analysis, with the exception of several areas of less resolution within Suloidea: **1)** the relationships within *Morus*; **2)** the relationship of *Papasula* relative to *Morus* and *Sula*; and **3)** the relationships within the ‘microcormorants’.

The results of the winning-sites, Templeton, and modified KH tests assessing the alternative constrained topologies are presented in Table 1. Note that for each set of constrained MPTs, only the *P*-value for the best fitting MPT is reported in order to make assessment of significance conservative. The *p*-values from the three paired-sites tests are all congruent in the rank order that the six suboptimal constrained topologies are placed in, which is also congruent with their rank order ascertained by the number of extra character changes implied by these topologies relative to the optimal unconstrained MPTs (Table 1). Topologies enforcing monophyly of Pelecaniformes, and monophyly of a penguin/plotopterid clade, represent the only two constrained topologies with KH test *p*/2 values >0.05, supporting the interpretation that these topologies would not be considered significantly worse fits to the present dataset than the optimal topologies under an SH test [76]. MPTs recovered under constraints matching the Livezey and Zusi [4] topology approach this threshold, with KH test *p*/2 values slightly below 0.05 (Table 1). However, a result where SH tests fail to reject an alternative topology (e.g., an inability to reject the null hypothesis) is difficult to interpret as positive evidence that the alternative tree is as good a fit to the character data as the optimal tree, as the SH test is known to be particularly conservative [76]–[78], [81]. Given these issues with interpretation, as well as the problems inherent in these paired sites tests as discussed above (see also [76], [78]), the results of these tests will not be discussed further.

**Table 1:** Results of the constrained analyses and pairwise tests of topologies.

Constrained Topology	Extra character changes implied relative to optimal MPTs	Winning-sites test *p*-value*	Templeton test *p*-value*	KH test *p*/2-value*
Hackett et al. [29]	149	<0.0001	<0.0001	<0.00005
*Anhinga*, Sulidae Monophyly	39	<0.0001	<0.0001	<0.00005
*Scopus*, *Balaeniceps*, *Pelecanus* Monophyly	28	0.0303	0.0179	0.00875
Livezey & Zusi [4]	21	0.3711	0.0834	0.04585
Pelecaniform Monophyly	9	0.3729	0.4428	0.2162
Penguin, Plotopteridae Monophyly	4	1.0000	0.6684	0.3463

*Note that *P*-values reported are from the best fitting tree of those in the set of constrained MPTs. See text for details.

### Relationships of Prophaethontidae and Phaethontidae

In the present analysis, Prophaethontidae is recovered as a paraphyletic grade leading to Phaethontidae, with *Prophaethon* more closely related to modern tropicbirds than to *Lithoptila*, a result slightly different from that of Bourdon et al. [26], who recovered a monophyletic Prophaethontidae. Only two unambiguous synapomorphies support a *Prophaethon* + Phaethontidae clade to the exclusion of *Lithoptila* (42:0–>1; 59:0–>1), both of which were discussed by Bourdon et al. [26]. A larger clade of Prophaethontidae and Phaethontidae is recovered as the sister taxon to Procellariiformes, similar to the results recovered by Bourdon [25] and Bourdon et al. [26]. This Procellariiformes + (Prophaethontidae + Phaethontidae) clade is supported by 15 unambiguous synapomorphies (57:0–>1; 102:0–>1; 114:1–>2; 138:0–>1; 150:0->1; 152:0–>1; 188:0–>1; 248:0–>2; 259:0–>1; 320:0–>1; 339:0–>1; 379:0–>1; 399:0–>1; 461:0–>1; 463:0–>1), three of which exhibit no homoplasy on the MPTs (57:0–>1; 399:0–>1; 463:0–>1). Several of these characters are briefly discussed below.

#### 57. Basioccipital, metotic process, foramen or notch for passage of arteria ophthalmica externa near lateral edge: present (0); absent (1) ([26]: character 24); see also ([64]: character 62)

Arteria ophthalmica externa is also known as the stapedial artery (see [82]). In most waterbirds, arteria opthalmica externa branches off of the internal carotid artery posterior to the metotic process, and perforates the lateral edge of the metotic process below the base of the paroccipital process as a distinct foramen or notch. The remaining portion of the internal carotid artery typically perforates the metotic process also as it passes rostrally. However, in Procellariiformes, Phaethontidae, and Prophaethontidae, arteria opthalmica externa passes lateral to the metotic process and does not perforate it. This may be related to the relative reduction of the metotic processes in these taxa, or the relative angle at which the internal carotid artery enters the head ([83]: p. 113).

#### 248. Ulna, relative proximodistal postions of distal condyles: condyles subequal in distal extent (0); condylus dorsalis significantly proximal to condylus ventralis (1); condylus dorsalis extended distally to condylus ventralis (2) ([64]: character 1530); ([25]: character 74)

A proximally located condylus dorsalis is present in both Spheniscidae and Plotopteridae. However, a condylus dorsalis that is situated significantly distally relative to condylus ventralis is only present in Procellariiformes and *Phaethon* among waterbirds. The condition in Prophaethontidae is unknown.

#### 259. Radius, small, distally directed tuberosity at cranioventral border of sulcus tendineus, with small fossa located distal and slightly caudal to it: absent (0); present (1) ([25]: character 75)

This tuberosity is low and rounded, and typically not as well developed as tuberculum aponeurosis. Its associated fossa is circular to slightly craniocaudally elongate. Though the fossa extends slightly onto the cranial edge of the distal radius, it typically does not extend to the caudal edge. The tuberosity is slightly more prominent in procellariforms than in *Phaethon*. Contra Bourdon [25], the derived state is also present in *Anhinga*. A similar fossa is also present in this area in *Gavia*, but it is not associated with a distinct tubercle as in taxa possessing the derived state. The condition in Prophaethontidae is unknown.

#### 399. Fibula, marked caudal offset of fibular shaft relative to long axis of tibiotarsus proximal to m. iliofibularis tubercule, in some cases resulting in a narrow, proximodistally elongate fissuriform foramen between tibiotarsus and fibula in lateral aspect: absent (0); present (1) ([64]: character 2195)

In Procellariiformes and *Phaethon* the proximal third of the fibula is markedly offset caudally relative to the long axis of the tibiotarsus or distal fibula. There can be some degree of variation in this caudal deflection, even between right and left elements of the same specimen (e.g., *Puffinus gravis* FMNH 364582). This deflection often, but not always, creates a visible gap between the proximal tibiotarsus and fibula in lateral aspect. Note that I do not recognize a distinction between subtle caudal deflection of the fibula as in states “a” and “b” of Livezey and Zusi ([64]: character 2195), as there is not a strong distinction between the morphology of taxa possessing either of these two character states, nor a clear morphological gap separating the states. The condition in Prophaethontidae is unknown.

#### 463. Pes, relative development of medial and lateral grooves on unguals (particularly ungual III): absent or weak (0); grooves strongly excavated into ungual (1) New Character

In most avian taxa, the medial and lateral grooves on the unguals are absent or weakly developed. However, in at least *Phaethon* and *Phoebastria*, these grooves are extremely well excavated. I am unable to assess the condition in the unguals of other procellariformes included in the present dataset. In *Phaethon* in particular, the degree of excavation is so strong as to divide the anteriormost tip of the ungual into two distinct dorsal and ventral points. There is typically an asymmetry in the relative development of medial and lateral grooves in any individual ungual.

Ten unambiguous synapmorphies support the monophyly of a Prophaethontidae and Phaethontidae clade in the present analysis (29:0–>1; 50:0–>1; 54:0–>1; 64:0–>1; 68:0–>1; 72:0–>1; 83:0–>1; 223:0–>2; 433:0–>1; 437:0–>1), four of which exhibit no homoplasy on the MPTs (50:0–>1; 54:0–>1; 68:0–>1; 72:0–>1). Several of these characters are discussed briefy below.

#### 29. Quadrate, orientation of the squamosal and otic condyles relative to the long axis of the skull: obliquely oriented, angle between 20-75° (0); nearly perpendicular, angle between 75-90° (1); nearly parallel, angle less than 20° (2) ([64]: character 148)

**Figure 7:**
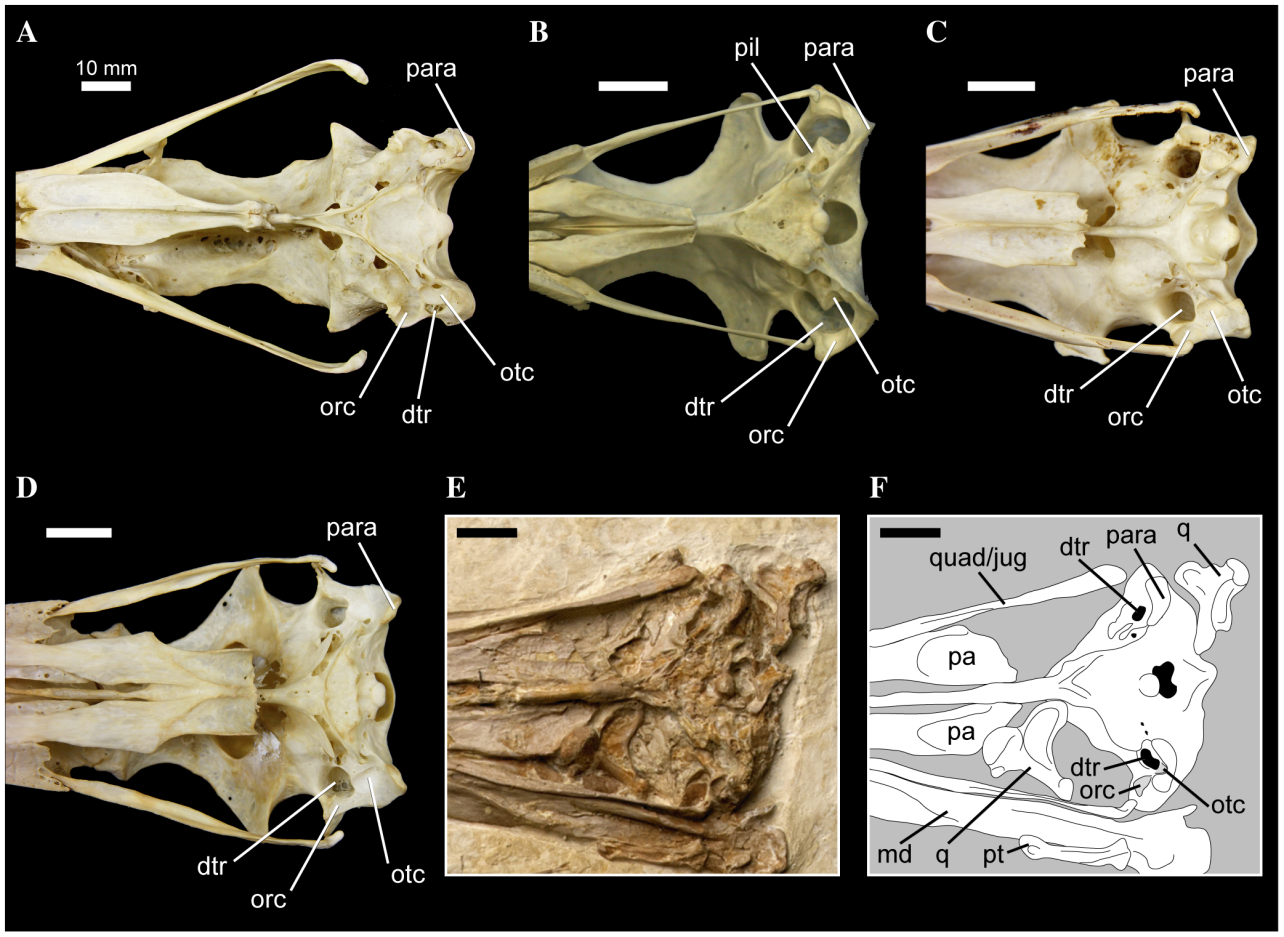
Pelecaniform skulls in ventral aspect. *Pelecanus occidentalis* FMNH 342303 (A), *Phaethon aethereus* FMNH 348136 (B), *Sula sula* FMNH 339372 (C), *Fregata magnificens* FMNH 339418 (D), and *Limnofregata azygosternon* FMNH PA755 (D, E). Scale bars equal 10 mm. Abbreviations: **dtr**, dorsal tympanic recess; **md**, mandible; **pa**, palatine; **para**, paroccipital process; **pil**, pila otica; **pt**, pterygoid; **orc**, orbital ( =  squamosal) cotyle; **otc**, otic cotyle; **q**, quadrate; **quad/jug**, quadratojugal/jugal.

In most waterbird taxa, the squamosal and otic condyles of the quadrate are obliquely oriented relative to the long axis of the skull. *Anhinga* is unique in that the squamosal condyle is shifted markedly forward, such that the angle formed by the long axis of the skull and a transverse line through the center of both condyles is extremely acute, and the condyles are nearly parallel to the skull's long axis. However, in Prophaethontidae, Phaethontidae, *Cochlearius*, and contra Livezey and Zusi ([64]: character 148), *Balaeniceps*, the quadrate condyles are oriented much more strictly mediolaterally to each other, and nearly perpendicular to the long axis of the skull (Figure 7B).

#### 50. Squamosal, relative length of rostral border of squamosal that joins zygomatic process and caudal wall of orbit: not elongated (0); elongate and thin, with constant thickness throughout ([26]: character 14)

Bourdon et al. ([26]: p. 166; character 14) noted that in both Phaethontidae and Prophaethontidae, the strut of bone connecting the zygomatic process to the caudal wall of the orbit is elongated relative to other waterbirds, and that in the latter taxon, this bony strut is also relatively uniform in thickness throughout its length. However, I chose only to recognize two states for this character (contra Bourdon et al. [26]), emphasizing the elongation of the rostral border of the squamosal in *Phaethon*, *Prophaethon*, and *Lithoptila*, noting that in *Phaethon* this process is thin and relatively consistent in thickness (Figure 7B). This bony strut also forms the rostrolateral border of the dorsal tympanic recess in Prophaethontidae and Phaethontidae.

#### 54. Squamosal/Prootic, pila otica elongated, strongly protruding caudoventrolaterally, so that cotyla quadratica otici faces laterally: absent (0); present (1) ([26]: character 30)

Prophaethontidae and Phaethontidae are unique among waterbirds in possessing a robust and elongate pila otica that protrudes caudoventrally and laterally ([26]: Figure 7A). This relative orientation results in a large portion of the otic cotyle facing laterally (Figure 7B). *Phoebastria* approaches the derived condition, though only the rostroventral-most portion of the otic cotyle is everted slightly laterally.

#### 68. Dorsal tympanic recess, greatly enlarged, much longer than broad, extending rostral to and between cotylae quadratica in a figure-8 shape: absent (0); present (1) ([26]: character 32)

**Figure 8:**
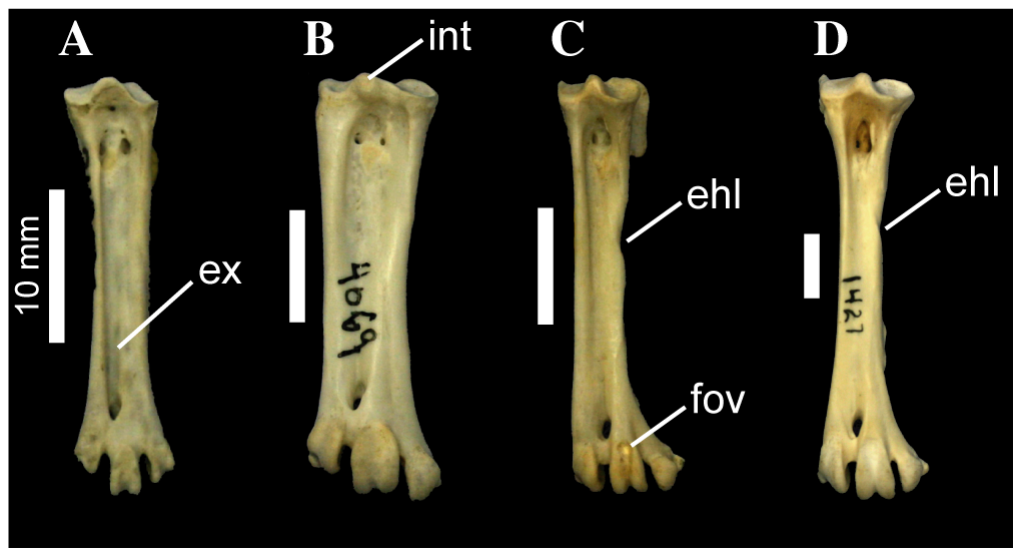
Pelecaniform right tarsometatarsi in cranial aspect. *Phaethon aethereus* FMNH 348136 (A), *Sula sula* FMNH 339372 (B), *Phalacrocorax africanus* FMNH 368742 (C), and *Phalacrocorax carbo* FMNH 339390 (D). Scale bars equal 10 mm. Abbreviations: **ehl**, furrow for the tendon of musculus extensor hallucis longus; **ex**, sulcus extensorius; **fov**, fovea at proximal end of trochlea metatarsi III; **int**, eminentia intercondylaris.

In *Phaethon*, *Prophaethon* and *Lithoptila*, the dorsal tympanic recess is greatly enlarged, and extends rostral to, and caudally between, the quadrate cotyles (Figure 7B). In ventral aspect, the outline of the dorsal tympanic recess in these taxa also takes on a slightly medially bent, figure-8 shape, with circular caudal and rostral portions, and a constriction at the rostral borders of the quadrate cotyles (Figure 7B). The rostral portion of the recess is slightly larger and more extensive medially.

#### 72. Foramen nervi maxillomandibularis location relative to entrance of recessus tympanicus rostralis: rostral (0); caudal (1) ([26]: character 29); ([64]: character 46); see also ([33]: character 27)

In most waterbird taxa, the foramen for the exit of the maxillomandibular nerve is located rostral to the opening for the rostral tympanic recess on the ventrolateral side of the braincase. However, in Prophaethontidae and Phaethontidae, foramen nervi maxillomandibularis exits just slightly caudally relative to the opening of the rostral tympanic recess. Note that I disagree with Bourdon et al.'s [26] codings for Diomedeidae and *Fregata*. In these taxa the foramen nervi maxillomandibularis exit is approximately in the same plane as the opening for the rostral tympanic recess, though of the waterbirds exhibiting the plesiomorphic state, these taxa most closely approach the derived condition. Bourdon et al. ([26]: p. 169) note that this character is variable within the Procellariidae. In some cormorants (e.g., *Phalacrocorax auritus*, *Phalacrocorax carbo*), the two foramina are nearly side-by-side, with foramen nervi maxillomandibularis only slightly rostral to the recessus tympanicus rostralis.

#### 437. Tarsometatarsus, relative development of distal end of sulcus extensorius in area of foramen vasculare distale: sulcus present but relatively shallow (0); suclus extremely deep (1) ([7]: character TMT5)

In most birds, sulcus extensorius typically becomes shallower moving distally along the tarsometatarsal shaft, and is very shallow near the area of foramen vasculare distale. However, in *Papasula* (including both adult and juvenile specimens; USNM 560862, 560863) and *Phaethon*, the sulcus is still extremely deep at its distal end (Figure 8A). This morphology is also present in the extinct prophaethontid, *Lithoptila* ([53]: p. 758; Figure 2M).

### Monophyly of Steganopodes

A monophyletic Steganopodes consisting of Pelecanidae, Fregatidae, Sulidae, Anhingidae, Phalacrocoracidae, and the extinct Plotopteridae, is recovered in the present analysis. Steganopodes is supported by 28 unambiguous synapomorphies (11:0–>1; 44:0–>1; 80:1–>2; 83:0–>1; 135:0–>1; 140:0–>1; 160:0–>1; 169:0–>1; 263:0–>1; 268:0–>1; 274:2–>1; 280:0–>1; 285:0–>1; 287:0–>1; 300:0–>1; 312:0–>1; 315:0–>1; 356:0–>1; 374:0–>1; 376:0–>1; 381:0–>1; 390:1–>0; 395:0–>1; 397:0–>2; 398:0–>1; 404:2–>0; 440:0–>1; 448:0–>1), though only one exhibits no homoplasy across the MPTs (374:0–>1). Several of these characters are discussed in more detail below.

#### 169. Coracoid, development of sulcus associated with cranial border of impressio ligamenti acrocoracohumeralis: absent or weak (0); strong sulcus present (1) ([64]: character 1276)

Most waterbird taxa have a clearly visible muscle scar on the craniolateral border of the acrocoracoid process, where the acrocoracohumeralis ligament attaches. In Steganopodes, a well-developed sulcus is associated with the impression for this liagmentous attachment. This sulcus is also clearly present in the Oligocene plotopterid *Plotopterum joaquinensis* (USNM 8927–cast of LACM 8927), but the sulcus is extremely weak or absent in *Tonsala hildegardae* (USNM 256518) and a large Miocene specimen from the Ashiya Formation referred to *Tonsala*? sp. (USNM 243775–cast of KMNH VP 200,003; see [5]). If *Plotopterum joaquinensis* is a basal member of Plotopteridae, as suggested by Olson and Hasegawa [5], this character distribution adds support to the hypothesis that the family has pelecaniform affinities, with the loss of the sulcus being a derived condition in some plotopterids (e.g., *Tonsala* and *Copepteryx*).

#### 268. Os carpi radiale, pneumatic foramina on distal surface: absent (0); present (1) ([64]: character 1563)

The distal face of os carpi radiale is pneumatic in *Pelecanus*, *Fregata*, *Sula*, and *Morus*. This pneumaticity is often developed as a large, shallowly rimmed opening, with additional foramina and trabeculae inside it. In *Phalacrocorax* and *Anhinga*, this surface is not pneumatic, but it is well excavated as a broad depression on the distal surface of os carpi radiale.

#### 374. Tibiotarsus, proximodistal length of foramen interosseum distale relative to foramen interosseum proximale: subequal or foramen interosseum distale slightly longer (0); foramen interosseum distale significantly longer (1); foramen interosseum distale significantly shorter, essentially occluded by its proximity to tibiotarsus (2) ([64]: characters 2129, 2130)

Steganopodes are unique among waterbirds in possessing a foramen interosseum distale that is elongate relative to foramen interosseum proximale. *Limnofregata* (WSGS U1-2001) also clearly possesses a relatively elongate foramen interosseum distale. *Pygoscelis* also approaches this derived condition, however. *Phaethon* and two distantly related, non-waterbird genera (*Pterocles* and *Turnix*) possess a short, and nearly occluded foramen interosseum distale [64].

#### 376. Tibiotarsus, morphology of tuberosity for attachment of proximomedial portion of retinaculum mm. extensorum: proximodistally elongate, raised crest (0); oval to circular scarred impression (1); tuberosity absent (2) ([64]: character 2133)

On the distal end of linea extensoria, proximomedial to the pons supratendineus (when present) most avians possess an impression or tuberosity that represents the anchor for the proximomedial portion of retinaculum mm. extensorum ( =  “retinaculum extensorium tibiotarsi”). This retinaculum is a tough, obliquely oriented fibrous arch under which the tendons for m. tibialis cranialis and m. extensor digitorum longus pass. In ratites tuberosities associated with the medial attachment of this retinaculum are absent. In most waterbirds, a linear, proximodistally elongate crest is present on the distal portion of linea extensoria that marks the medial attachment of this retinaculum. In *Gavia*, Steganopodes, and several non-waterbird taxa the proximomedial tuberosity for attachment of retinaculum mm. extensorum is not linear, but rather an oval to round scarred tuberosity. Note that I disagree with Livezey and Zusi ([64]: character 2133) regarding the condition in penguins, *Balaeniceps* and *Phaethon*, all of which I interpreted as possessing the plesiomorphic state. A distinct lineate scar is most clearly present in *Balaeniceps*. The scar is fainter and less marked in penguins and *Phaethon*, but clearly present and proximodistally elongate.

#### 440. Tarsometatarsus, concave incisure in the medial side of the distal edge of trochlea metatarsal II: absent (0); present (1) ([64]: character 2351)

**Figure 9:**
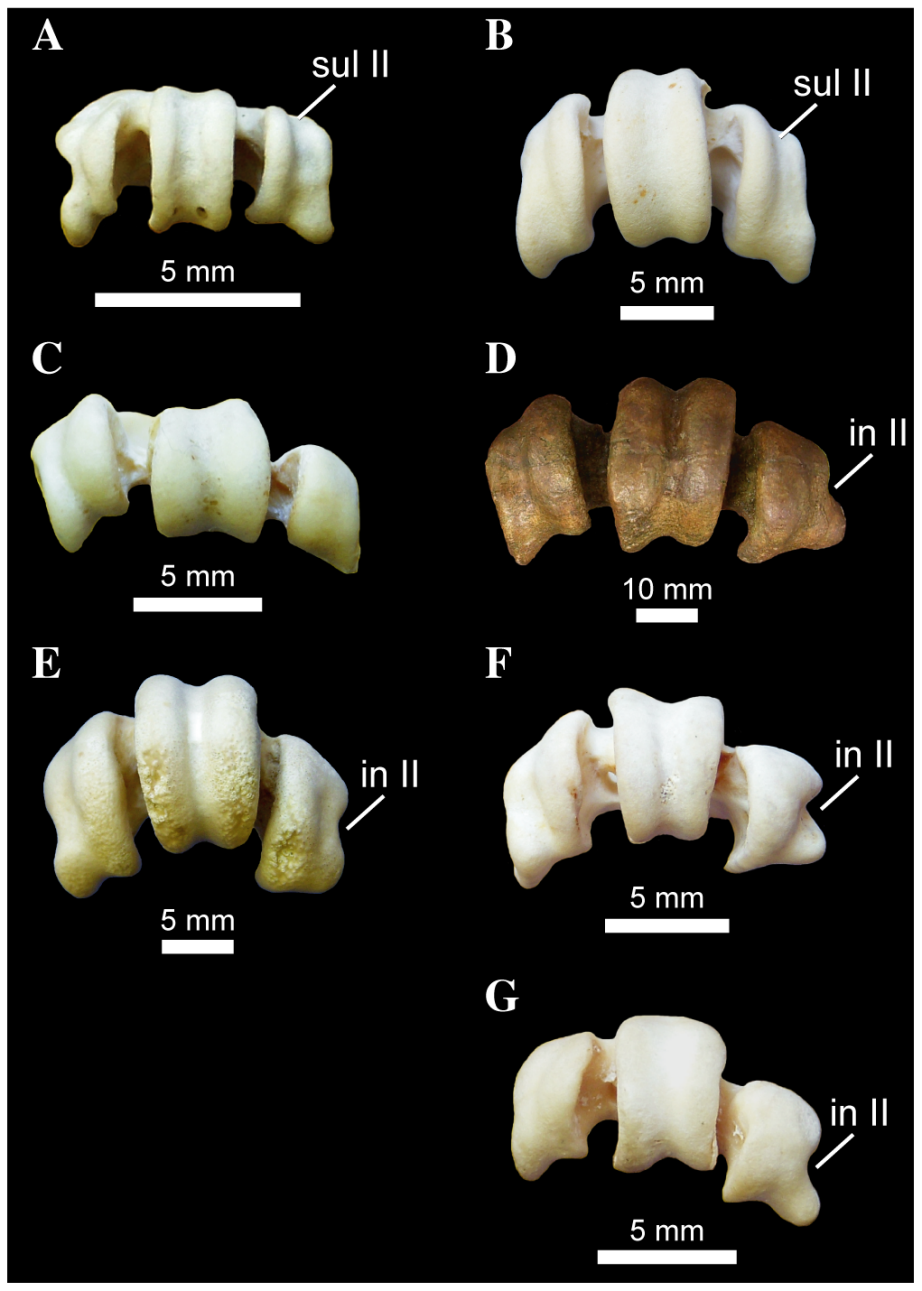
Waterbird right tarsometatarsi in distal aspect. *Phaethon aethereus* FMNH 348136 (A), *Phoebastria nigripes* FMNH 339601 (B), *Eudyptula minor* FMNH 106505 (C), *Copepteryx hexeris* USNM 243773–cast of KMNH VP 200,001 (D), *Pelecanus erythrorhynchos* FMNH 445082 (E), *Sula sula* FMNH 339372 (F), and *Fregata minor* FMNH 339421 (G). Scale bars equal 5 mm for (A–C, E–G), and 10 mm for (D). Abbreviations: **in II**, incisure in the medial side of the distal edge of trochlea metatarsal II; **sul II**, sulcus on the dorsal face of trochlea metatarsal II.

**Figure 10:**
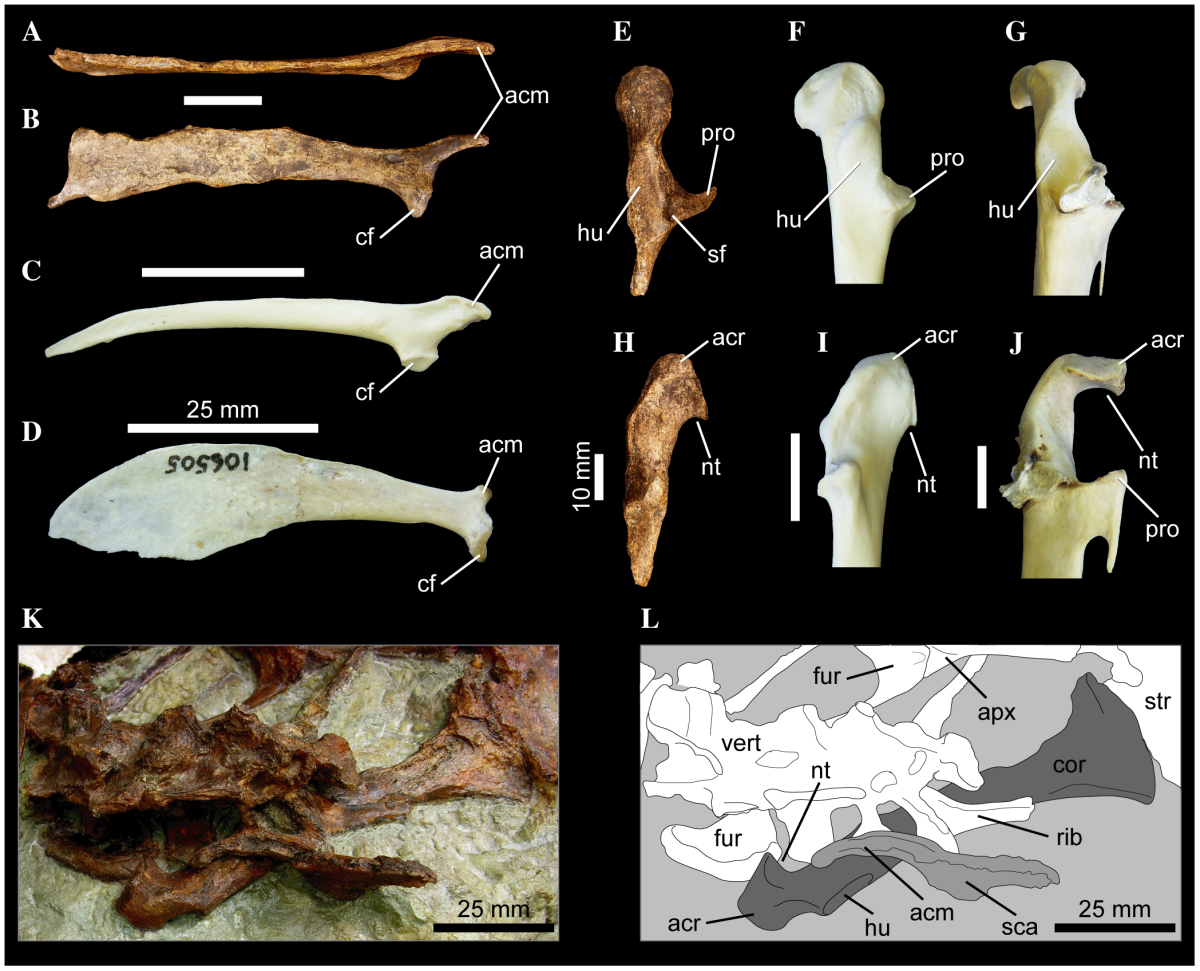
Pectoral girdle elements of several waterbird taxa. Right scapula of *Tonsala hildegardae* USNM 256518 (A, B), *Anhinga anhinga* FMNH 339387 (C), and *Eudyptula minor* FMNH 106505 (D), in dorsal (A), and lateral (B–D) aspects. Left coracoid of *Tonsala hildegardae* USNM 256518 (E, H), *Anhinga anhinga* FMNH 339387 (F, I), and *Pygoscelis antarctica* FMNH 390994 (G, J), in lateral (E–G), and dorsal (H–J), aspects. Partial skeleton of *Copepteryx hexeris* USNM 486682–cast of KMNH VP 200,006 (K, L) in dorsal aspect. Scale bars equal 25 mm (A–D), 10 mm (E–J), and 25 mm (K, L). Abbreviations: **acm**, acromial process; **acr**, acrocoracoid process; **apx**, cranial apex of sternal keel; **cf**, coracoid facet; **cor**, coracoid; **fur**, furcula; **hu**, humeral articular facet; **nt**, medial notch to furcular facet; **pro**, procoracoid process; **vert**, dorsal vertebrae; **rib**, dorsal rib; **sf**, scapular facet; **sca**, scapula; **str**, sternum.

Among waterbirds, *Pelecanus*, *Fregata*, and Suloidea are unique in the presence of a distinct notch on the medial side of trochlea metatarsal II (Figure 9). This incisure is often best viewed in distal aspect, and is more pronounced in Suloidea than in *Fregata* and *Pelecanus*. Contra Livezey and Zusi ([64]: character 2351), this notch is not present in *Phaethon*, though this taxon does have a slightly expanded and medially everted plantar process on trochlea metatarsal II. Interestingly, the derived feature is present in *Copepterxy hexeris* (Figure 9D; [5]: Figure 10C), but it is not clear whether it is present in *Phocavis* ([60]: Figure 2F).

### Monophyly and Relationships of the Plotopteridae

Despite being highly incomplete in some cases (e.g., *Phocavis*, *Plotopterum*), the four purported plotopterids are recovered together in a monophyletic clade that is supported by eight unambiguous synapopmorphies (176:0–>1; 178:0–>1; 409:0–>1; 410:0–>1; 414:0–>1; 427:0–>1; 435:0–>1; 445:3–>2), three of which exhibit no homoplasy on the MPTs (178:0–>1; 409:0–>1; 435:0–>1). Several of these characters are described in more detail below. A variety of additional characters in the highly modified forelimbs of plotopterids also variably support the monophyly of the Plotopteridae (since many of these elements are unknown in *Phocavis* and *Plotopterum*, their status as synapomorphies are partially dependant upon method of character optimization). Many of these characters (e.g., characters 107, 159, 164, 165, 198, 210, 220, 229, 248, 256) are also present in penguins, interpreted on the MPTs recovered as being derived independently in the two clades.

#### 156. Scapula, shape of acromial process: blunt to rectangular process (0); extremely compressed dorsoventrally, elongate, finger-like morphology (1). New Character

The acromial process of the plotopterids *Tonsala hildegardae* (USNM 256518; [50]: Figure 4A) and *Copepteryx hexeris* (USNM 486682–cast of KMNH VP 200,006; [5]) is distinct not only for being extremely craniocaudally elongate, but the process is also strongly dorsoventrally compressed, giving the acromion a finger-like morphology in medial or lateral aspect (Figure 10). The elongate acromial process of these two plotopterids is also distinctly concave laterally (Figure 10A). Interestingly, scapulae referred to the late Eocene stem-Cariamidae taxon *Elaphrocnemus phasianus* also possess an extremely elongate acromial process that is somewhat dorsoventrally compressed, though not to the degree seen in plotopterids ([84]: Figure 1). The acromial process of *Elaphrocnemus phasianus* is also not distinctly concave laterally as in Plotopteridae ([84]: Figure 1). Mayr and Mourer-Chauviré [84] suggest that an elongate acromial process may function in strengthening the triosseal canal and acrocoracoid process of the coracoid.

Note that as the derived state is unknown in *Plotopterum* and *Phocavis*, this character state is only reconstructed as a synapomorphy of Plotopteridae under ACCTRAN character optimization in trees where *Copepteryx* and *Tonsala* are sister taxa.

#### 176. Coracoid, relative convexity of caudal portion of the triosseal canal: flat to weakly concave (0); distinctly convex and swollen (1). New Character

Howard [43] originally noted that in alcids, sphenisciformes, and *Plotopterum joaquinensis* (USNM 8927–cast of LACM 8927), the caudal portion of the triosseal canal is swollen and convex, particularly in the former two taxa. Howard ([43]: p. 69) suggested that this convexity might be an adaptation to wing-propelled diving, as it “tends to narrow and deepen the passageway for the pectoral tendon, and presumably afforded support to the tendon so as to strengthen the upstroke of the wing in swimming”. Based on the topologies of the MPTs, the derived state is inferred to have evolved independently in Sphenisciformes and Plotopteridae. The condition in *Phocavis* is unknown [60].

#### 178. Coracoid, relative orientation of facies articularis clavicularis: faces cranioventrally and medially (0); caudal (sternal) end of facet is strongly everted, enhancing the cranial and medial components to its orientation (1). New Character

In plotopterids, including *Plotopterum joaquinensis* (USNM 8927–cast of LACM 8927), *Tonsala hildegardae* (USNM 256518), a large Miocene specimen from the Ashiya Formation referred to *Tonsala*? sp. (USNM 243775–cast of KMNH VP 200,003; see [5]), and *Copepteryx hexeris* (USNM casts; [5]), the caudal end of the furcular facet of the coracoid is strongly everted medially and cranially, changing the orientation of the facet, and creating a distinct caudal notch between the coracoid shaft and furcular facet in dorsal aspect (Figure 10; see also [50]: Figure 4B). The condition in *Phocavis* is unknown [60]. Both *Anhinga* and Spheniscidae approach the derived condition.

#### 272. Ossa metacarpalia, degree of fusion and proximodistal extent of metacarpal I (alulare): distinguishable, extending no farther distal than symphysis intermetacarpalis proximalis (0); diminutive, synostotic with metacarpal II and proximal digit I (1); distinguishable, comparatively elongate, extending significantly distal to symphysis intermetacarpalis proximalis (2) ([64]: character 1580; see also characters 1749, 1751)

In penguins, metacarpal I is synostotic with metacarpal II and proximal digit I. In the plotopterids *Tonsala hildegardae* (USNM 256518; [50]) and *Copepteryx hexeris* (USNM 486682–cast of KMNH VP 200,006; [5]), metacarpal I is extremely elongate, extending distally past symphysis intermetacarpalis proximalis. This morphology is very similar to *Mancalla* [50]. However, this elongate morphology of metacarpal I is also present in the extinct stem penguin, *Icadyptes salasi*, and at least embryonically in some extant penguins ([85]: p. 145; Figures 12, 15). Also, contra Livezey and Zusi ([64]: character 1580), state “2” is present in *Gavia* as well.

Note that as the derived state is unknown in *Plotopterum* and *Phocavis*, this character state is only reconstructed as a synapomorphy of Plotopteridae under ACCTRAN character optimization in trees where *Copepteryx* and *Tonsala* are sister taxa.

#### 409. Tarsometatarsus, relative mediolateral position of eminentia intercondylaris ( =  “intercotylar prominence”) on tarsometatarsus: at or near midline of tarsometatarsus (0); distinctly lateral to midline of tarsometatarsus (1). New Character

The intercotylar process is a variably robust process that projects proximally from between the proximal cotyles of the tarsometatarsus as a rounded triangular eminence. As noted by Goedert ([60]: p. 98), the intercotylar prominence of *Phocavis* and *Copepteryx hexeris* (USNM 243773–cast of KMNH VP 200,001; [5]: Figure 10) is located slightly lateral to the midline of the tarsometatarsus, unlike the condition in other avian taxa.

#### 410. Tarsometatarsus, relative proximodistal position of tubercle for insertion of m. tibialis cranialis on the dorsal face of metatarsals II and III: proximal, near the end of the tarsometatarsus (0); at or just proximal of the midpoint of the tarsometatarsus (1) ([64]: character 2236); ([7]: character TMT10)

Penguins and *Fregata* are unique among extant waterbirds in that the tubercle where m. tibialis cranialis inserts on the dorsal face of the tarsometatarsus is relatively distally positioned, near the midpoint of the element. The recovery of these morphologies as convergent in the MPTs is supported by the fact that the stem-frigatebird, *Limnofregata*, possesses the plesiomorphic morphology (USNM 22753; WSGS U1-2001). The plotopterids *Copepteryx hexeris* (USNM 243773–cast of KMNH VP 200,001; [5]: Figure 10), and *Phocavis maritimus* ([60]: Figure 2) also have tubercles that are more distally placed than taxa possessing the plesiomorphic state.

Warheit ([7]: character TMT10) suggested that an additional distinct state of this character exists for *Sula*, *Papasula*, and *Phaethon*, which all have the tubercle for insertion of m. tibialis cranialis placed so far proximally that its proximal edge extends to the distal border of the foramina vascularia proximalis. However, the distinction between this condition and that of other taxa where the tubercle is very near the distal edge of the foramina is extremely subtle with continuous variation, thus I did not recognize this additional state.

#### 414. Tarsometatarsus, relative development of dorsal rim of lateral cotyle: rim present (0); rim extremely reduced or absent (1) ([64]: character 2253); ([7]: character TMT3)

A reduced to completely obsolete dorsal rim of the lateral cotyle of the tarsometatarsus is present in *Fregata*, *Podiceps*, and contra Livezey and Zusi ([64]: character 2253), *Gavia*. In *Gavia*, the rim is completely absent and the cotylar surface itself slopes onto the dorsal face of the proximal tarsometatarsus. Some specimens of *Limnofregata* (e.g., WSGS U1-2001) still possess a weak dorsal rim to the lateral cotyle, though the holotype of *Limnofregata* has a very reduced dorsal rim (USNM 22753). *Limnofregata* has been tentatively coded as possessing the plesiomorphic condition in this analysis. The derived condition is also present in *Phocavis* ([60]: p. 100; Figure 2), and the plotopterid *Copepteryx hexeris* (USNM 243773–cast of KMNH VP 200,001; [5]: Figure 10). *Balaeniceps* and *Pelecanus* approach the derived condition.

#### 427. Tarsometatarsus, mediolateral position of crista medialis hypotarsi relative to medial proximal cotyle of tarsometatarsus: crista located at midline or slightly lateral to midline through medial cotyle (0); crista located medial to midline of medial cotyle (1). New Character

**Figure 11:**
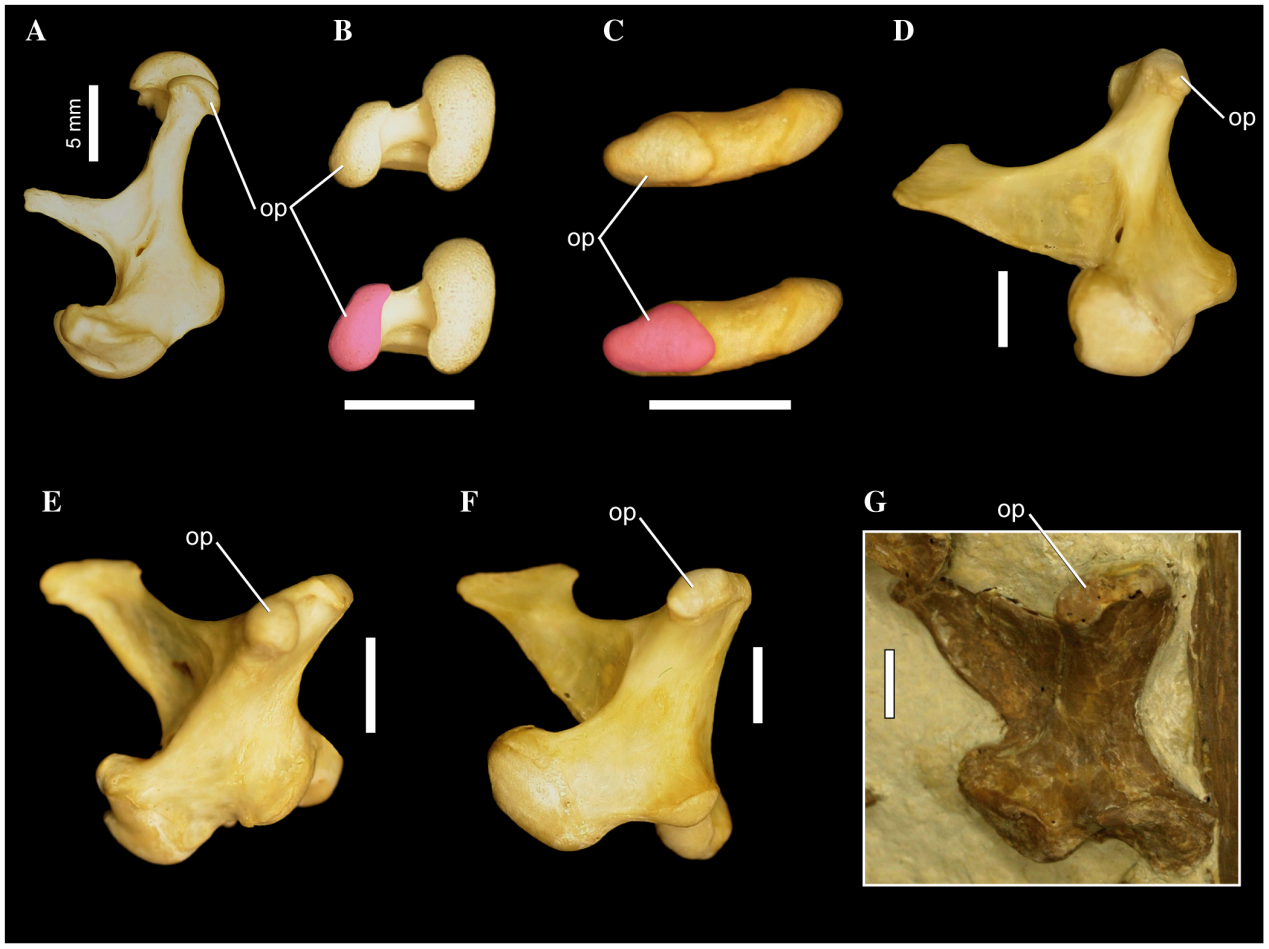
Right quadrates of several waterbird taxa. *Phalacrocorax carbo* FMNH 339390 (A, B), *Fregata minor* FMNH 339421 (C, D, F), *Phoebastria nigripes* FMNH 339601 (E), and *Limnofregata azygosternon* FMNH PA 719 (G), in medial (A, D); dorsal (B, C); and medial/posterodorsal (E–G) aspects. In (B, C) the articular surface of the otic process of the quadrate is highlighted in magenta. Scale bars equal 5 mm. Abbreviations: **op**, otic process.

**Figure 12:**
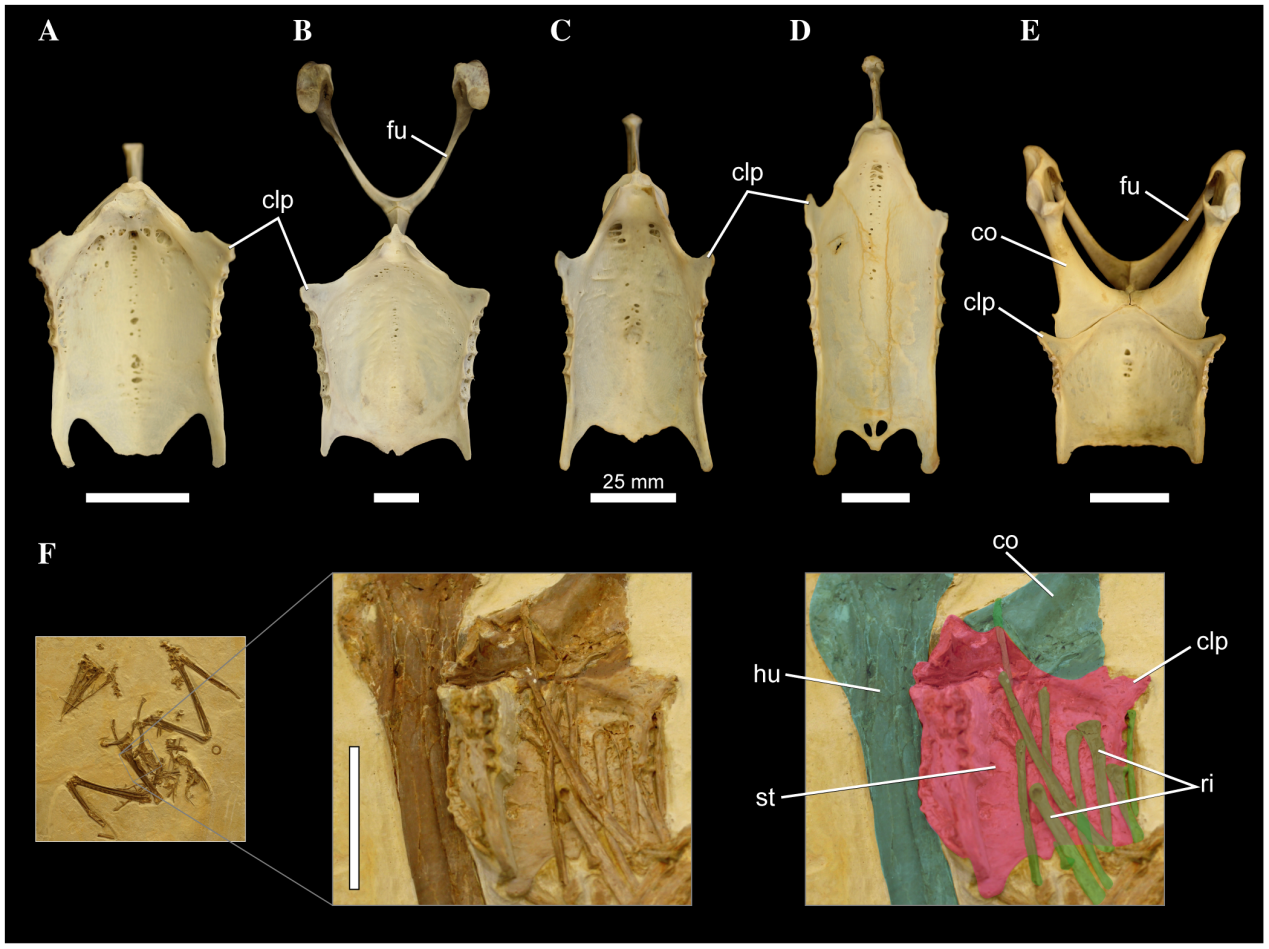
Sterna of several waterbird taxa. *Ciconia abdimii* FMNH 368771 (A), *Pelecanus erythrorhynchos* FMNH 445082 (B), *Sula sula* FMNH 339372 (C), *Morus serrator* FMNH 339366 (D), *Fregata minor* FMNH 339421 (E), and *Limnofregata azygosternon* FMNH PA 755 (F), in dorsal (A–E), and dorsal and slightly left lateral (F) aspects. Individual elements are color-coded in (F). Scale bars equal 25 mm. Abbreviations: **clp**, craniolateral process; **co**, coracoid; **fu**, furcula; **hu**, humerus; **ri**, ribs; **st**, sternum.

Olson and Hasegawa ([5]: p. 746) originally noted that the crista medialis hypotarsi of the plotopterid *Copepteryx hexeris* (USNM 243773–cast of KMNH VP 200,001; [5]: Figure 10D) is located relatively medially on the proximal tarsometatarsus compared to most taxa. Indeed, this morphology is also present in *Phocavis* ([60]: Figure 2). The derived state is also present in modern penguins, though the hypotarsus in these birds is strongly reduced. However, the distribution among extinct, stem penguins appears to be more complex [85], [86]. The plesiomorphic state is present in *Palaeeudyptes antarcticus* ([85]: Figure 14), *Palaeeudyptes klekowskii* ([86]: Figure 7), *Palaeeudyptes gunnari* ([86]: Figure 8), *Anthropornis nordenskjoeldi* ([86]: Figures 5, 6), *Marambiornis exilis* ([87]: Figure 6) *Delphinornis larseni* ([86]: Figure 10), *Delphinornis gracilis* ([86]: Figure 11), and *Delphinornis artowskii* ([86]: Figure 12); ([87]: Figure 6), while the derived state is present in *Palaeospheniscus patagonicus* ([85]: Figure 15), and *Paraptenodytes antarcticus* ([88]: Figure 21). The distribution of this character amongst fossil and extant penguins, and the phylogeny of Ksepka et al. [85] would suggest that the plesiomorphic state was present throughout much of the “spine” of basal penguin phylogeny, and that the derived state evolved in a slightly more inclusive group than crown penguins. This would imply that the derived morphology is convergent between penguins and plotopterids, as is recovered in the present analysis.

#### 435. Tarsometatarsus, relative dorsoplantar position of fossa metatarsi I on tarsometatarsal shaft: plantar, with some minor medial component (0); primarily medial (1) ([64]: character 2314)

In almost all avian taxa, the fossa for attachment of metatarsal I to the tarsometatarsus is located on the plantar to medioplantar edge of the tarsometatarsal shaft. Only in basal Avialae (e.g., *Archaeopteryx*, *Confuciusornis*, *Hesperornis*) and the plotopterids *Copepteryx hexeris* and *Phocavis* (USNM 243773–cast of KMNH VP 200,001; [5], [60]) is this fossa located primarily on the medial border of the tarsometatarsal shaft.

As is evident from figure 2, a monophyletic Plotopteridae is recovered as the sister taxon to the Phalacrocoracoidea ( =  Phalacrocoracidae + Anhingidae). Contra Mayr [23], a close relationship between plotopterids and penguins is not supported, despite the numerous similarities in forelimb morphology mentioned briefly above. The clade uniting the Plotopteridae and Phalacrocoracoidea is not particularly well supported in the bootstrap or Bremer support analyses, but is none-the-less supported by six unambiguous synapomorphies (174:1–>0; 204:0–>1; 285:1–>0; 314:0–>1; 391:0–>1; 412:0–>2). Several of the characters supporting a close relationship between the Plotopteridae and Phalacrocoracoidea are described in more detail below.

#### 204. Humerus, anterior surface of crista bicipitalis ( =  “intumescentia”): inflated and bulbous (0); weakly convex or planar (1) ([64]: character 1405)

In most waterbird taxa, the cranioventral portion of the proximal humerus in the area of crista bicipitalis ( =  “intumescentia”) is enlarged as a cranially convex swelling. This distinct bulbous swelling is absent, however, and the area of crista bicipitalis is relatively planar in several taxa, including *Phoebastria*, *Anhinga*, *Phalacrocorax*, the extinct plotopterids *Copepteryx hexeris* (USNM 486682–cast of KMNH VP 200,006; [5]) and *Tonsala hildegardae* (USNM 256518; [50]), and the extinct *Protoplotus beauforti* [42].

#### 314. Pelvis, extreme lateral expansion of cranial end of preacetabular process of ilium, coupled with reduction or “waisting” of preacetabular process in region just cranial to acetabulum: absent (0); present (1) ([64]: character 1828)

In most waterbird taxa the cranial end of preacetabular process of the ilium is wider mediolaterally than the region just anterior to the acetabulum. In *Phalacrocorax* and *Anhinga*, the cranial end is relatively widened even more, and the region anterior to the acetabulum is reduced, such that the mediolateral width of the former is greater than two times the width of the latter. This also gives the pelvis a “dumbbell”, or “hourglass” shape in dorsal aspect. The derived morphology is also clearly present in the plotopterid *Copepteryx hexeris* (USNM 243773–cast of KMNH VP 200,001; [5]: Figure 7A). Contra Livezey and Zuis ([64]: character 1828), I did not consider *Fregata* to possess the derived state. Although the preacetabular process of the ilium of *Fregata* is slightly expanded, the ilium is not strongly narrowed, or “waisted” caudal to this expansion as in the other taxa possessing the derived state.

#### 391. Tibiotarsus, symmetry of medial and lateral margins of proximal end of trochlea cartilaginis tibialis: relatively symmetrical (0); markedly asymmetric at proximal end, with distinct lateral kink, or displacement, of proximal end of medial margin (1) ([64]: character 2172)

Trochlea cartilaginis tibialis is a sulcus on the caudal side of the distal tibiotarsus that houses cartilago tibilalis, a fibrocartilaginous block over which the tendons of m. gastrocnemius and superficial flexor muscles pass [82]. Deep flexor tendons of the pedal digits also pass through canals in cartilago tibialis [82]. In most waterbirds trochlea cartilaginis tibialis is a variably well-developed sulcus with sharp and distinct medial and lateral ridges. These ridges are also typically subparallel to each other. However, in *Gavia*, penguins, *Anhinga*, *Phalacrocorax*, and to a lesser degree *Phoebastria* and *Puffinus*, the medial ridge of this trochlea is kinked sharply laterally at its proximal end, and is not parallel to the corresponding lateral edge. In *Anhinga* and *Phalacrocorax*, this kink occurs more distally than in penguins, and the resulting laterally displaced medial edge of the trochlea extends further proximally up the distal tibiotarsal shaft. Although the distal tibiotarsus of the plotopterid *Copepteryx hexeris* (USNM 243773–cast of KMNH VP 200,001; [5]) is slightly damaged, a laterally inflected medial margin of trochlea cartilaginis tibialis is still apparent.

#### 412. Tarsometatarsus, relative proximal extents of cotyles: subequal (0); medial cotyle proximal to lateral cotyle (1); medial coytle distal to lateral cotyle (2) ([64]: character 2250; see also character 2248)

Among waterbirds, *Phaethon*, some penguins (e.g., *Pygoscelis* but not *Eudyptula*), *Anhinga*, *Phalacrocorax*, and the plotopterids *Copepteryx hexeris* and *Phocavis* (USNM 243773–cast of KMNH VP 200,001; [5]: Figure 10A; [60]: Figure 2A) are unique in that the medial cotyle of the tarsometatarsus is situated at a level slightly distally to the lateral cotyle.

### Relationships of *?Borvocarbo stoeffelensis* and Phalacrocoracidae

The late Oligocene ?*Borvocarbo stoeffelensis* [54], [55] is recovered as the sister taxon to Phalacrocoracidae in the present analysis. Three unambiguous synapomorphies support the monophyly of a ?*Borvocarbo* + Phalacrocoracidae clade (91:0–>1; 446:1–>2; 464:0–>1), one of which (464) exhibits no homoplasy in the MPTs. These characters are described in more detail below.

#### 91. Mandible, surangular, area at posteromedial end of attachment of M. adductus mandibulae externus profundus: indistinct or lacking tuberosity (0); presence of a single robust, knob-like tuberosity (1); presence of a large, bipartite flange (2) ([33]: character 41); see also ([89]: Figure 54D)

In most waterbirds, the area where M. adductus mandibulae externus profundus inserts on the mandible, just anterior to the quadrate cotyles, is relatively unmarked. However, in several species of *Phalacrocorax*, including ‘microcormorants’, *P. brasilianus*, *P. auritus*, and *P. gaimardi*, a dorsally prominent, knob-like tuberosity is present. The tuberosity is particularly tall and well developed in *P. brasilianus* and *P. auritus*. Mayr ([55]: Figure 4A,C) describes the presence of this tuberosity in ?*Borvocarbo stoeffelensis*.

In *Phoenicopterus*, the area of insertion of m. adductus mandibulae externus profundus is marked by an extremely robust, bipartite flange. The medial and lateral portions of this flange are divided by a longitudinal midline sulcus. The medial portion has multiple ridges and extends slightly medially and dorsally. The lateral portion is much larger and flange-like, being somewhat flattend dorsoventrally. It extends primarily laterally, but also slightly caudodorsally. The lateral portion also has oblique, buttressing ridges on both its dorsal and ventral portion.

#### 446. Pes, relative lengths of digits III and IV: digit III longer than digit IV (0); digit IV slightly longer than digit III (1); digit IV significantly longer than digit III, often by nearly the entire distal phalanx of digit IV (2) ([64]: character 2371)

In most avian taxa, digit III is longer than digit IV. However, within waterbirds, Procellariformes, loons, grebes, penguins, and some Pelecaniformes (though not *Fregata* and *Limnofregata*) have a digit IV that is slightly longer than digit III. *Phalacrocorax* are unique in having a digit IV that is significantly longer than digit III, often by nearly an entire phalanx. This character state is also present in ?*Borvocarbo stoeffelensis* [54], [55]. Contra Livezey and Zusi ([64]: character 2371), *Balaeniceps* possesses the plesiomorphic state, and has a relative length of digit III/IV that is similar to both *Ciconia* and *Scopus*. Also, contra Livezey and Zuis ([64]: character 2371), digit III is longer than digit IV in *Fregata*, as is also the case for *Limnofregata*. Though character state “1” exhibits some homoplasy on the MPTs, being independently derived at least twice, character state “2” is inferred to have only evolved once, in the clade uniting *Phalacrocorax* and ?*Borvocarbo stoeffelesnsis*, with no reversals to the other two states.

#### 464. Pes, strong dorsoventral compression of phalanges of pes: absent (0); present (1). New Character

In most waterbirds, the shafts of the phalanges of the pes are subcylindrical. However, in *Phalacrocorax* and ?*Borvocarbo stoeffelensis* [54], ([55]: p. 939), the phalanges are stongly dorsoventrally compressed. Note that both *Gavia* and *Anhinga* approach the derived condition. However, the latter does not exhibit the same degree of compression seen in *Phalacrocorax*, and in the former, typically only the distalmost phalanges are strongly compressed.

Within Phalacrocoracidae, a basal dichotomy is recovered separating ‘microcormorants’ and remaining members of the family. Two unambiguous synapmorphies support the monophyly of the ‘microcormorants’ (94:0–>1; 430:1–>2), while four diagnose the remaining cormorants to the exclusion of ‘micorcormorants’ (254:0–>1; 340:0–>1; 381:1–>2; 445:3–>2). A basal split between ‘microcormorants’ and all other cormorants within the Phalacrocoracidae has not been recovered previously in a morphological analysis, though Mayr [55] recently suggested that morphological data might support such a topology. Several related phylogenetic analyses based on mitochondrial data support this basal dichotomy [2], [16], [18], [34]. However, most of these [2], [16], [18] have not rigorously tested the monophyly of ‘microcormorants’, including only a single exemplar for the group. Only Holland et al. [34] have included more than one ‘microcormorant’ in a phylogenetic analysis and recovered ‘microcormorant’ monophyly. Several characters that support the monophyly of ‘micorcormorants’, and those supporting monophyly of a clade of phalacrocoracids exclusive of ‘microcormorants’, are described below.

#### Character 94. Mandible, relative size of fossa aditus: large (0); small, fossa typically no larger than associated neurovascular canal (1) ([33]: character 46); see also ([64]: character 689)

A small fossa aditus on the medial side of the posterior mandible is present in ‘microcormorants’. This trait is convergently present in *Fregata*.

#### 254. Radius, proximally concave indentation in edge of humeral cotyle created by robust tubercle on proximal radius caudodorsal to biceps tubercle: absent (0); present (1). New Character

In ‘microcormorants’ this tubercle is mound-shaped, and abuts but does not deform the edge of the humeral cotyle. In all other Phalacrocoracidae, the tubercle is larger, and a proximodistally elongate, upside-down triangle shape, and it does create a proximally concave indentation in the edge of the humeral cotyle.

#### 340. Femur, laterally everted, tab-like tuberosity associated with insertion scar of m. iliotrochantericus medialis: absent (0); present (1). New Character, though see ([33]: characters 113, 119)

Most waterbird taxa possess a distinct muscle scar for the insertion of m. iliotrochantericus medialis on the lateral surface of the proximal femur, near the craniodistal end of trochanter femoris. In *Phalacrocorax*, with the exception of ‘microcormorants’, this insertion scar is associated with a distinct, tab-like tuberosity projecting laterally from the craniodistal edge of the muscle scar.

#### 381. Tibiotarsus, relative distal extent of condyles: subequal (0); distal end bent medially, and condylus medialis protruding slightly further distally than condylus lateralis (1); condylus medialis protruding significantly further distally, giving the edge of the distal tibiotarsus a near “L”-shaped outline in cranial or caudal aspect (2) ([23]: character 45); ([64]: character 2145)

Among waterbirds, a variety of taxa, including *Gavia*, penguins, *Pelecanus*, Suloidea and the extinct pelecaniforms *Copepteryx hexeris* and *Limnofregata*, possess a tibiotarsus with a slight medial kink distally and a medial condyle that projects further distally than the lateral condyle. This condition is also present in the ‘microcormorants’. However, in all other *Phalacrocorax*, the distal protrusion of the medial condyle is extremely accentuated, resulting in the distal condyles being strongly offset in cranial or caudal aspect. Though states “0” and “1” exhibit homoplasy across the MPTs, state “2” is unique to *Phalacrocorax* exclusive of ‘microcormorants’.

#### 430. Tarsometatarsus, concavity of lateral margin of distal tarsometatarsal shaft in dorsal perspective: concave, distally curving smoothly to lateral face of trochlea of digit IV, resulting in symmetrical (or nearly so) medial and lateral borders of distal tarsometatarsal shaft (0); sublinear, trochlea IV splays laterally only slightly, resulting in asymmetry with medial border (1); linear, trochlea IV extends almost straight distally at the distal end of the tarsometatarsus (2) ([64]: character 2289); ([7]: character TMT15)

In most avian taxa, the medial and lateral borders of the distal tarsometatarsal shaft are nearly symmetrical, and the lateral border is concave in dorsal aspect, smoothly curving into the lateral edge of trochlea of digit IV. However, among waterbirds, most Procellariiformes (note I disagree with Livezey and Zusi [64] regarding the coding for *Oceanites*), loons, grebes, penguins, *Pelecanus*, *Sula*, *Anhinga*, and most *Phalacrocorax* have a sublinear lateral border, and a trochlea metatarsal IV with only a slight lateral splay, which makes the distal tarsometatarsal shaft appear markedly asymmetric in dorsal aspect. The presence of this morphology in loons and grebes is almost certainly related to the high degree of mediolateral compression of the tarsometatarsal shaft in these taxa. ‘Microcormorants’ (e.g., *Phalacrocorax africanus*, *P. pygmaeus*, *P. melanoleucos*) have a more extreme morphology where trochlea IV extends nearly straight distally, such that the entire lateral border of the tarsometatarsus has a linear appearance in dorsal or plantar aspect. Interestingly, extinct probable stem members of Phalacrocoracidae such as ?*Borvocarbo stoeffelensis* [54], [55], and *Nectornis miocaenus* ([90]: Plate 39, Figures 6, 7), possess the intermediate morphology, supporting the interpretation of state “2” as a synapomorphy of ‘microcormorants’. Although state “1” of this character exhibits homoplasy across the MPTs, state “2” is uniquely present in ‘microcormorants’.

#### 445. Tarsometatarsus, relative distal extents of trochleae metatarsals: II < III > IV, and II subequal to IV (0); II < III > IV, and II much less than IV (1); II < III ≥ IV, and II >IV (2); II > III ≥ IV (3) ([64]: character 2361); ([23]: character 49); ([7]: character TMT9)

Despite the multitude of states for this character, most waterbird taxa are consistent in possessing a trochlea metatarsal II that does not extend further distally than trochlea metatarsal III (character states 0, 1, 2). However, *Fregata*, Sulidae, and *Anhinga* are unique in that metatarsal II is relatively elongate, and its trochlea extends distally past that of metatatarsal III (Figure 8B). Interestingly, most *Phalacrocorax* possess state “2”, with the exception of ‘microcormorants’ (e.g., *Phalacrocorax africanus*, *P. pygmaeus*, *P. melanoleucos*), the flightless cormorant, *Phalacrocorax harrisi*, and also the recently described fossil taxon ?*Borvocarbo stoeffelensis* [55], which may be a stem member of Phalacrocoracidae. These taxa all possess a metatarsal II that extends further distally than metatarsal III, suggesting that state “2” present in most cormorants may represent a reversal in the clade excluding ‘microcormorants’.

The fact that the synapomorphies supporting a basal split between ‘microcormorants’ and remaining Phalacrocoracidae are distributed throughout the skeleton may argue against these relationships being erroneously inferred due to ecomorphological convergence. However, several characters (characters 254, 340) diagnosing the ‘non-microcormorant’ clade relate to the robust development of tubercles associated with muscle insertion, and thus could potentially be due to body-size differences between the two groups rather than phylogenetic history. Although the monophyly of ‘micorcormorants’ is only weakly supported at present, being diagnosed by two synapomorphies, in some cases (e.g., character 430), the morphology of potential fossil stem cormorants [54], [55], [90] appears to support interpretation of these characters as synapomorphies rather than plesiomorphies spread across the stem of Phalacrocoracidae.

At present, the remaining relationships within Phalacrocoracidae are weakly supported (Figures 2–6). Interestingly, several clades recovered are congruent with recent estimates of cormorant phylogeny based on mitochondrial data [16], [18], [34], including a sister-taxon relationship between *Phalacrocorax auritus* and *P. brasilianus*; and a clade including *P. bougainvilli*, *P. magellanicus*, *P. atricepts*, and *P. albiventer*. However, inconsistencies still remain (e.g., the relative position of *P. carbo*; the absence of a close relationship between *P. pelagicus* and *P. penicillatus*). Rigorously assessing the species-level relationships within Phalacrocoracidae will require additional taxon and character sampling in both morphological and molecular datasets. Furthermore, serious discrepancies noted between the current dataset and that of [33] warrant a critical reappraisal of the character definitions and codings present in the latter dataset (see also discussion in [34]).

### Monophyly of Suloidea

A monophyletic Suloidea (superfamily Suloidea *sensu* [3]; also considered parvorder Sulida [4], or suborder Sulae [5]) is well supported in the present analysis (Figure 2). This clade is supported by 20 unambiguous synapomorphies (2:1–>0; 8:1–>2; 22:0–>1; 35:0–>1; 41:0–>1; 47:0–>1; 63:0–>1; 64:0–>1; 66:0–>1; 71:0–>1; 87:0–>1; 96:0–>1; 112:2–>0; 158:1–>0; 181:2–>0; 358:0–>1; 367:3–>1; 373:1–>0; 458:0–>1; 461:0–>1), six of which exhibit no homoplasy on the MPTs (22:0–>1; 35:0–>1; 41:0–>1; 71:0–>1; 96:0–>1; 458:0–>1). Several of these characters are described in more detail below.

#### 22. Palatine, dorsal surface of palatine a nearly flat, horizontal plate: no (0); yes (1) ([23]: character 11)

In most waterbirds, the dorsal surface of the palatine is relatively tall dorsoventrally, particularly at its posterior end where it contacts the parasphenoid rostrum. In suloids, by contrat, this area of the palatine is relatively flat, forming a horizontal plate, with the exception of very short dorsal extensions near the posteromedial edge of the palatine at the pterygoid articulation. Note that this character describes the dorsal surface of the palatine, and not the ventral surface, which often exhibits a distinct ventral crest (see e.g., [64]: character 440). For example, *Sula sula* (FMNH 339372) has the ventral expansion, but the palatine is still flat dorsally.

#### 35. Quadrate, orbital process reduced in dorsoventral thickness, tapering to a point distally: no (0); yes (1) ([3]: character 13), ([23]: character 20); see also ([64]: characters 533, 539); ([26]: character 40)

In most waterbird taxa, the orbital process of the quadrate is fairly broad dorsoventrally, and relatively uniform in thickness throughout its length, or slightly expanded at its tip. In *Anhinga*, *Phalacrocorax*, and Sulidae, the orbital process is conspicuously reduced in dorsoventral thickness, and more triangular in medial or lateral aspect, tapering to a point distally. Note that Livezey and Zusi ([64]: character 533) do not consider the derived state to be present in Sulidae. However, the orbital process of the quadrate is clearly reduced in dorsoventral thickness in sulids, as originally noted by Cracraft [3], and typically tapers to a finer point than in some cormorants.

#### 41. Frontal, suture with lacrimal: facing laterally (0); facing ventrally and not obliterated in adults (1); facing ventrally and obliterated in adults (2) ([26]: character 9); see also ([64]: character 564)

In most waterbirds, the lacrimal articular surface on the frontal is exposed laterally. In *Anhinga*, Sulidae, and juvenile *Phalacrocorax* the articular surface for the lacrimal on the frontal faces distinctly ventrally. In adult *Phalacrocorax* the suture between the lacrimal and frontal is completely fused.

#### 47. Processus postorbitalis, length and orientation: long and ventrally oriented (0); short and ventrolaterally oriented (1) ([7]: character Skl 6)

In all Suloidea, with the notable exception of *Papasula*, the postorbital process is relatively shortened and projects ventrolaterally. In most waterbirds, the postorbital process is elongate, with a distal end that is projected primarily ventrally.

#### 66. Paroccipital processes, distal tips protrude strongly caudally: no (0); yes (1) ([3]: character 17), ([23]: character 14), ([26]: character 21), and ([64]: character 132)

In most waterbirds, the distal tips of the paroccipital process are directed straight ventrally or nearly so. In Gaviidae, Anhingidae, Phalacrocoracidae, and Sulidae, the distal tips of the paroccipital processes are distinctly everted caudally (Figure 7C). The paroccipital processes of *Pelecanus* (e.g., FMNH 445082) extend significantly caudally relative to other taxa (e.g., *Fregata*), but the distal articular tips of these processes are still directed ventrally, in contrast to the condition in taxa with the derived state.

#### 71. Rostral tympanic recess, relative development of posterolateral flange of ala parasphenoidale: large, posterolaterally expanded flange (0); extremely reduced or absent (1) ([3]: character 14); see also ([26]: character 28), and ([64]: character 229)

The posterolateral flange of ala parasphenoidale forms the rostral and ventral rim of the anterior tympanic recess, and is typically located ventrally to the exit foramen for the trigeminal nerve (foramen nervi maxillomandibularis). In most waterbirds, this flange is well developed and everted strongly laterally, creating a posterolaterally facing ring of bone. There is quite a bit of variation in the morphology of this flange. For instance, in *Pelecanus*, *Phoebastria*, and *Fregata*, the flange is not as circular, and its ventral component is more laterally extensive than its reduced dorsal component. Also, in many taxa (e.g., Ardeidae, Threskiornithidae) the lateral edge of this flange approaches or contacts the quadrate shaft. However, in Suloidea this flange is extremely reduced, particularly in its lateral extent, or completely absent.

#### 96. Atlas, morphology of dorsal rim of atlantal body: dorsal rim incomplete, with a broad gap between paired transverse ligament tuberosities (0); dorsal rim complete, resulting in peforate atlantal body (1) ([64]: character 771)

**Figure 13:**
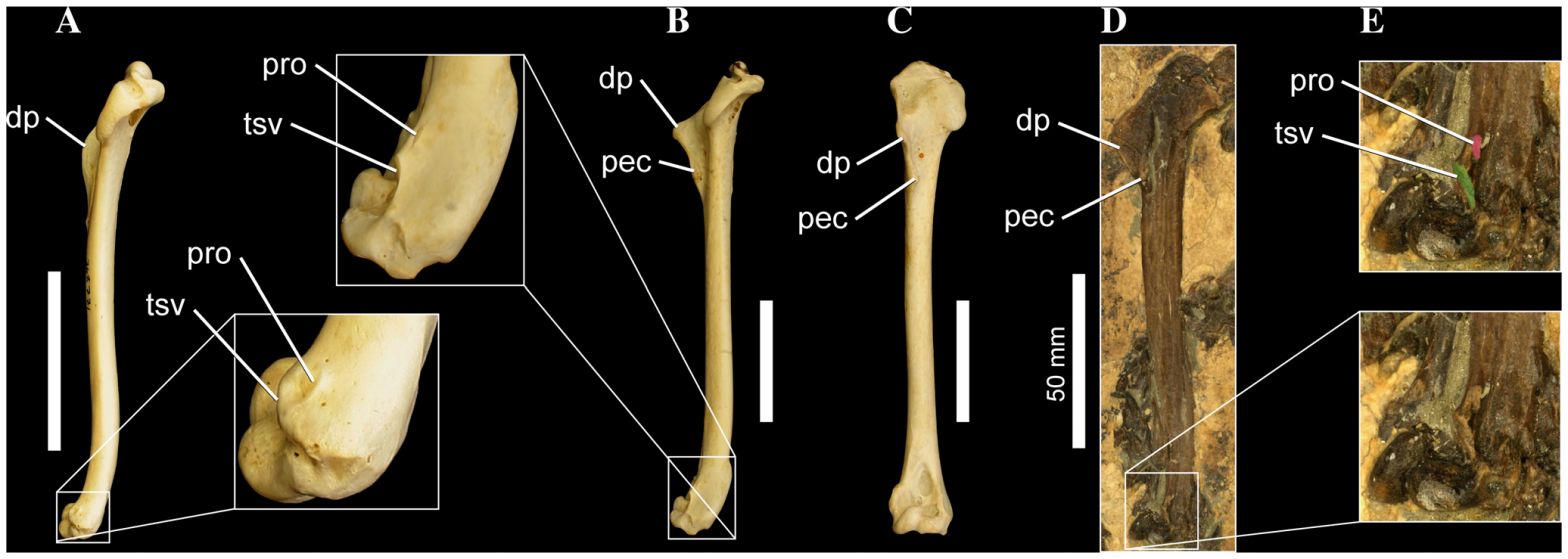
Right humeri of several waterbird taxa. *Ciconia abdimii* FMNH 368771 (A), *Fregata magnificens* FMNH 339418 (B, C), and *Limnofregata azygosternon* UWGM 6919 (D, E), in medial (A, B; with distal portion enlarged), anterior (C), and anteromedial (D, E; with distal portion enlarged, and color-coded in E). Scale bars equal 50 mm. Abbreviations: **dp**, deltopectoral crest; **pec**, tubercle for attachment of m. pectoralis superficialis, pars deep; **pro**, scar for attachment of m. pronator superficialis; **tsv**, tuberculum supracondylare ventrale.

In most avian taxa, the dorsal rim of the atlantal body is not continuous between the transverse ligament tuberosities, creating a U-shaped gap where the atlas receives the dens of the axis. This gap is thus also continunous with the neural canal of the atlas. However, in Suloidea, the dorsal rim of the atlantal body is complete and closed off from the neural canal, resulting in a circular foramen perforating the main body of the atlas, which receives the dens of the axis. This atlantal foramen is also present in *Cacatua galerita* ([64]: Figure 13B). The plesiomorphic condition is also present in the holotype of *Limnofregata hasegawai* (GMNH PV 170; see [59]: Figure 5).

#### 458. Pes, proximodistal length of IV-1 relative to III-1: IV-1 distinctly shorter than III-1 (0); IV-1 subequal in length to III-1 (1); IV-1 distinctly longer than III-1 (2). New Character

In most avian taxa, phalanx IV-1 is significantly shorter than III-1. However, in Suloidea, phalanx IV-1 is subequal in length to phalanx III-1, and in *Podiceps*, IV-1 is significantly longer than III-1. Among suloids, *Anhinga* possesses the relatively shortest IV-1, though it is still much more similar to other suloids than to taxa that possess the plesiomorphic state. Interestingly, the late Oligocene ?*Borvocarbo stoeffelensis* [54], [55] and most *Phalacrocorax* (though apparently not *Phalacrocorax africanus*) possess a phalanx IV-1 that is slightly longer than phalanx III-1, though not considerably so. This relative elongation in *Phalacrocorax*, along with relative elongation of the other phalanges of digit IV, is likely what contributes to this taxon's apomorphically elongated digit IV (see character 446).

### Relationships within the Sulidae

A monophyletic Sulidae is well supported in the present analyses (Figures 2,3). Sulidae is also strongly supported as the sister taxon to a clade comprised of Phalacrocoracoidea and Plotopteridae. Support for *Morus* is robust, with this genus being diagnosed by 16 unambiguous synapomorphies (3:0–>1; 48:0–>2; 108:0–>2; 113:0–>1; 152:1–>2; 177:0–>1; 186:0–>1; 193:0–>1; 235:0–>1; 236:0–>1; 245:1–>0; 251:0–>1; 363:0–>1; 365:0–>1; 383:0–>1; 408:1–>2), nine of which exhibit no homoplasy on the MPTs (3:0–>1; 48:0–>2; 108:0–>2; 177:0–>1; 193:0–>1; 235:0–>1; 236:0–>1; 363:0–>1; 383:0–>1). A single synapomorphy unites a clade of *Morus bassanus* and *Morus capensis* in the present dataset (297:1–>0; the absence of pneumaticity associated with caudomedial [i.e., contra-articular] face of the antitrochanter), though this character is quite labile across the MPTs (C.I. = 0.143). This grouping is identical to that tentatively proposed by Nelson [91] (see also Warheit [7], who was unable to resolve the relationships within *Morus*), though it contrasts with the sister taxon relationship of *Morus capensis* and *Morus serrator* recovered by Friesen and Anderson [6] in their analysis of partial cytochrome *b* sequences of sulids. The genus *Sula* is also strongly supported, diagnosed by 10 unambiguous synapomorphies (5:0–>1; 48:0–>1; 87:1–>2; 180:0–>1; 201:0–>1; 221:0–>1; 281:0–>1; 335:0–>1; 346:0–>1; 424:0–>1), 4 of which exhibit no homoplasy on the MPTs (48:0–>1; 201:0–>1; 221:0–>1; 346:0–>1). Relationships within *Sula* are well resolved, and congruent with both previous analyses of morphological data [6], [7], and partial sequences of the mitochondrial gene cytochrome *b* [6].

*Papasula abbotti* is resolved as the sister taxon to *Sula* in the present analysis (Figure 2), identical to the topology recovered by Warheit [7], but at odds with the sister taxon relationship with *Morus* recovered by Friesen and Anderson [6]. However, the position of *Papasula* is not particularly well supported, and the MPTs of the pelvic anatomical partition analysis support the placement of *Papasula* as sister taxon to all other Sulidae (Figure 6). Six unambiguous synapomorphies support a sister group relationship between *Papasula* and *Sula* (112:0–>1; 122:1–>0; 190:0–>1; 192:0–>1; 339:0–>1; 430:0–>1), only one of which exhibits no homoplasy on the MPTs (192:0–>1). Several of these characters are discussed below.

#### 112. Sternum, degree of pneumaticity of incisurae intercostales: absent (0); present, few small foramina (1); present, heavily pneumatized with numerous foramina (2) modified from ([64]: character 1115)

A variety of waterbird taxa, including *Phoebastria*, *Phaethon*, *Phoenicopterus*, Ardeidae, Threskiornithidae, *Ciconia*, *Scopus*, *Balaeniceps*, *Pelecanus*, and *Fregata* are characterized by incisurae intercostales that are heavily pneumatized (character state “2”). An additional character state “1”, from the original character description of Livezey and Zusi ([64]: character 1115) is included here to describe the condition present in *Sula* and *Papasula*. In these two genera, pneumatic foramina are present in the incisurae intercostales, but they are often extremely small and few in number, and appear to represent an intermediate condition between taxa that lack this pneumaticity altogether (e.g., *Morus*), and taxa that possess heavily pneumatized incisurae intercostales.

#### 190. Coracoid, relative mediolateral width of labrum externum relative to dorsoventral height: wide and narrow (0); mediolaterally thin and dorsoventrally tall, forming a semilunate to triangular facet (1) ([7]: character COR1)

In *Papasula* and *Sula*, the labrum externum of the coracoid is relatively shortened mediolaterally compared to most waterbird taxa, and also much higher dorsoventrally. In *Pelecanus* and *Scopus* a similar condition is present, though in these taxa the labrum externum is not as high dorsoventrally. Also, in both *Scopus* and *Pelecanus* the labrum externum is situated more medially compared to most waterbird taxa, at the medialmost point of the sternal edge of the coracoid.

#### 192. Coracoid, intersection of anterior intermuscular line with labrum externum: intermuscular line intersects lateral portion of labrum externum (0); intermuscular line intersects labrum externum more medially, near midpoint of labrum externum (0) ([33]: character 56)

In *Papasula* [92] and *Sula*, the intersection of the anterior intermuscular line with labrum externum is located medially relative to other waterbird taxa.

### Relationships of *Limnofregata*

*Limnofregata* is robustly supported as the sister taxon to a monophyletic *Fregata* in the present analysis (Figure 2). 18 character states are reconstructed as unambiguous synapomorphies of the *Limnofregata*/*Fregata* clade in the set of most parsimonious trees (30:0–>1; 101:2–>1; 102:0–>1; 103:1–>0; 108:0–>1; 114:1–>2; 133:1–>2; 134:1–>0; 155:1–>0; 201:0–>2; 206:2–>1; 221:0–>2; 223:0–>2; 293:0–>1; 316:2–>1; 394:0–>1; 404:0–>1; 408:1–>2). Of these, 6 synapomorphies exhibit no homoplasy within the dataset (30:0–>1; 108:0–>1; 201:0–>2; 221:0–>2; 293:0–>1; 394:0–>1). Several of these characters are described in more detail below.

#### Character 30. Quadrate, shape of otic head in dorsal aspect: round or bulbous (0); compressed anteroposteriorly and distinctly elongate mediolaterally (1). New Character

Most waterbirds have a quadrate otic head that is rounded or subspherical in dorsal aspect, and is not significantly broader mediolaterally than long anteroposteriorly. An anteroposteriorly compressed and mediolaterally elongate otic head is present in *Fregata*. A specimen of *Limnofregata azygosternon* (FMNH PA 720) includes a well-preserved right quadrate that is exposed in posterodorsal and medial view (Figure 11). The otic process is clearly visible, and possesses the mediolaterally elongate morphology present in *Fregata*.

#### 101. Number of cervical vertebrae: 13 or 14 (0); 15 or 16 (1); 17 or more (2) modified from ([64]: character 798)

As originally noted by Olson [35], *Limnofregata* and *Fregata* both possess 15 cervical vertebrae. Nearly all other Pelecaniformes and Ciconiiformes (with the exception of *Scopus*) possess 17 or more cervical vertebrae.

#### 102. Osseous bridge from processus transversus to processus articularis caudalis on third cervical vertebra: absent (0); present (1) ([93]: character 52; Fig 6D). See also ([23]: character 24); ([64]: character 806)

On the third cervical of *Fregata* there is a distinct strut of bone that extends from the posterior side of the transverse process to the caudal end of the neural arch, just anterior to the postzygapophysis. This strut thus forms the lateral border of a dorsoventrally open foramen. In cervical 4, the strut is not complete, with only small prongs protruding posteriorly from the transverse process and anteriorly from the caudal neural arch, resulting in a partially open foramen. In *Fregata*, the medial border of this foramen formed by the neural arch is also pneumatized. The derived state is also present in cervicals 3–4 of *Limnofregata hasegawai* (GMNH PV 170; [59]: Figure 5). In addition to *Fregata* and *Limnofregata*, this osseous strut is present in *Phaethon*, *Ciconia*, Threskiornithidae, some procellariforms, and *Gallus*.

#### 103. Cervical vertebrae 8–11 with processus carotici ankylozed along the midline, forming an osseous canal: no (0); yes (1) ([23]: character 25)

In several waterbird families, including Ardeidae, Threskiornithidae, *Balaeniceps*, Anhingidae, Pelecanidae, and Sulidae, the carotid processes on the anteroventral side of the centra of the mid-cervical vertebrae extend medially and fuse at the midline, resulting in an anteroposteriorlly open canal. Based on the topology of the MPTs, the presence of the plesiomorphic condition in *Limnofregata* and *Fregata* is interpreted as a reversal. In *Fregata*, the carotid processes of cervicals 8–11 approach each other gradually moving posteriorly through the vertebral column, but never form an osseous canal when they eventually meet (∼cervical 12) and form a single midline ridge.

#### 108. Sternum, shape and relative craniocaudal length to mediolateral width of dorsal surface of sternal body: rectangular, sternal body longer than wide (0); square-shaped, sternal body wider than long (1); elongate rectangular, sternal body more than twice as long than wide (2) modified from ([64]: characters 1099, 1100, 1101)

Most waterbird taxa have a main sternal body that is longer anteroposteriorly than wide mediolaterally. The three extant species of *Morus* are unique in having an extremely elongate sternal body that is rectangular-shaped. *Fregata* is unique in possessing a sternum that is abbreviated craniocaudally, such that the anteroposterior length of the sternal body is less than its mediolateral width, and also less than the overall length of the coracoid. The holotype specimen of *Limnofregata azygosternon* includes a sternum that is preserved in left ventrolateral aspect, and is clearly abbreviated craniocaudally as in *Fregata* ([35]: Figures 2, 16, 17). An additional specimen of *Limnofregata azygosternon* (FMNH PA 755) preserves a sternum exposed in dorsal aspect, with the right coracoid in articulation and several fragmentary portions of ribs lying on the dorsal surface (Figure 12). As in USNM 22753, the sternum of FMNH PA 755 is box-like and mediolaterally wider than long anteroposteriorly. Olson ([35]: p. 2) first recognized the box-like and mediolaterally wide morphology of the sternal body as a similarity between *Limnofregata* and *Fregata*, and included this character in his diagnosis of the family Fregatidae, which included both taxa.

#### 114. Number of costal facets on sternum: four (0); five (1); six (2); seven (3) ([64]: character 1119)

A variety of waterbird taxa, including most procellariforms, *Phaethon*, *Phoenicopterus*, *Eudocimus*, several *Sula*, *Fregata*, and *Limnofregata* possess six costal facets on the sternum. Based on the topologies of the MPTs, this character state is optimized as a synapomorphy of a *Fregata*/*Limnofregata* clade.

#### 133. Sternum, relative convexity of ventral carinal margin in lateral aspect: moderately convex (0); nearly straight (1); extremely convex, approaching semicircular profile (2) modified from ([64]: character 1195); see also ([7]: character STN2)

An extremely convex sternal keel is recovered as a synapomorphy of a *Fregata*/*Limnofregata* clade. However, this character state is convergently present in *Scopus*, *Ciconia*, and Threskiornithidae.

#### 134. Sternum, apex carinae of sternum pointed and projecting far rostrally to coracoid sulci: no (0); yes (1) ([23]: character 33); ([64]: character 1198)

A sternum with an apex carina that extends rostrally beyond the coracodi sulci is present in *Gavia*, *Podiceps*, Sphenisciformes, *Ciconia*, *Balaeniceps*, *Phaethon*, *Pelecanus*, and Suloidea. Based on the topologies of the MPTs, a reversal to the plesiomorphic character state is optimized as a synapomorphy of a *Fregata*/*Limnofregata* clade.

#### 155. Scapula, relative cranial extension of acromion: short, does not extend cranial to articular facies for the coracoid (0); elongate, extends well past articular facies for the coracoid (1) ([64]: character 1245)

Several waterbirds, including *Balaeniceps*, *Pelecanus*, and Suloidea possess a relatively elongate acromial process of the scapula, which extends cranially past the rounded articular facet for the coracoid (Figure 10C). Based on the topologies of the MPTs, a reversal to the plesiomorphic character state is optimized as a synapomorphy of a *Fregata*/*Limnofregata* clade.

#### 201. Humerus, tuberculum m. pectoralis superficialis, pars deep (see [89]: p. 15, Figure 13) depth: anterior surface of humeral shaft medial and distal to tuberculum relatively smooth, without depression (0); medial and distal edge of tuberculum slightly raised, with groove-like depression along its edges on the humeral shaft (1); deep groove medial and distal to tuberculum, with distal portion of tuberculum hypertrophied as a round swelling (2) ([7]: character HUM6), see also ([64]: character 1400)

Members of the genus *Sula* are unique in possessing a shallow groove medial and distal to the oval muscle scar for insertion of m. pectoralis superficialis, pars deep. However, in *Fregata*, this groove is extremely deeply excavated, and the distal portion of the muscle scar is rounded and protrudes as a strong tubercle. A shallow depression is present in some specimens of *Phalacrocorax carbo* (e.g., FMNH 339390), but this groove is not nearly as pronounced as that present in *Sula*. Olson ([35]: p. 21; Figure 20) originally described a tubercle on the distal portion of the deltopectoral crest of *Limnofregata* that he considered homologous to the tubercle for insterion of m. pectoralis superficialis, pars deep that is present in *Fregata*. Indeed, both the holotype (USNM 22753) and paratype (UWGM 6919) specimens of *Limnofregata azygosternon* include well preserved humeri that have the distinctive tubercle and associated mediodistal groove present in *Fregata*.

#### 206. Humerus, relative development and shape of deltopectoral crest: slightly protruding, low and rounded (0); strongly protruding and triangular (1); extremely reduced (2) ([23]: character 39); see also ([64]: character 1374)

Both *Fregata* and *Limnofregata* (Figure 13) possess strongly protruding deltopectoral crests that are triangular in outline in dorsal or ventral aspect. This character state is convergently present in *Phoebastria* and *Puffinus*.

#### 221. Humerus, shape of tuberculum supracondylare ventrale in ventral aspect: relatively flat or planar (0); convex cranially, particularly on the distal half of the tuberculum (1); distal half of tuberculum distinctly concave, giving the tuberculum a triangular, ‘pointed’ appearance in ventral aspect (2) ([7]: character HUM17)

Tuberculum supracondylare ventrale provides the attachment point for ligamentum collaterale ventrale, which attaches to the ventral cotyle of the proximal ulna [82]. Howard [94], cited in ([7]: p. 36) suggested that this tubercle is the “attachment of the pronator brevis”, which is incorrect. This muscle originates on a small scar ventral and caudal to tuberculum supracondylare ventrale (see [82], [89]). In most waterbird taxa, the cranial face of tuberculum supracondylare ventrale is flat. In members of the genus *Sula*, the distal portion of the tubercle is convex. *Fregata* is unique among waterbirds in that the distal half of the tubercle is concave cranially, which gives the proximal portion of the tubercle a triangular shape, and the middle portion of the tubercle a ‘pointed’ appearance in ventral view (Figure 13). The holotype (USNM 22753) of *Limnofregata azygosternon* includes complete right and left humeri, and the paratype (UWGM 6919) specimen includes a right humerus, all of which are preserved in cranioventral aspect. The distinct concave morphology of tuberculum supracondylare ventrale typical of *Fregata* is evident in all three humeri.

#### 223. Humerus, relative location of muscle scar for insertion of m. pronator superficialis ( =  “m. pronator brevis”): caudal to tuberculum supracondylare ventrale (0); caudal and distal to tuberculum supracondylare ventrale and developed as a small tubercule (1); only slightly caudal, and distinctly proximal to tuberculum supracondylare ventrale (2). New Character

The muscle scar for m. pronator superficialis is a small, proximodistally elongate elliptical scar or depression located on the ventral side of the distal humerus, proximal to the depressions for m. flexor carpi ulnaris and m. protracter profundus. Owre ([89]: p. 34) suggested that the relative volume and distal extent of m. pronator superficialis may correlate with efficiency of soaring and gliding flight. In most waterbird taxa, the scar for m. pronator superficialis is located immediately caudal to tuberculum supracondylare ventrale, and sometimes slightly distal to the midpoint of the tuberculum. In *Phoebastria*, this muscle scar is located well distal to the distal end of tuberculum supracondylare ventrale. In *Fregata* and *Phaethon*, there is no muscle scar caudal and ventral to the tuberculum supracondylare ventrale, and instead the scar for m. pronator superficialis is located slightly ventral and distinctly proximal to the tuberculum. Once again, the right humeri of both the holotype (USNM 22753) and paratype (UWGM 6919) specimens of *Limnofregata azygosternon* preserve clear muscle scars for m. prontaor superficialis on their distal end (Figure 13; see also [35]: Figure 18), and these scars are clearly located proximal to tuberculum supracondylare ventrale, as in *Fregata* and *Phaethon*. The distal portion of the left humerus of USNM 22753 is not preserved as well and a muscle scar for m. pronator superficialis cannot be discerned.

#### 293. Manus, proximodistally elongate fenestra on the distal third of the blade of II-1: absent (0); present (1). New Character, though see ([35]: p. 24)

**Figure 14:**
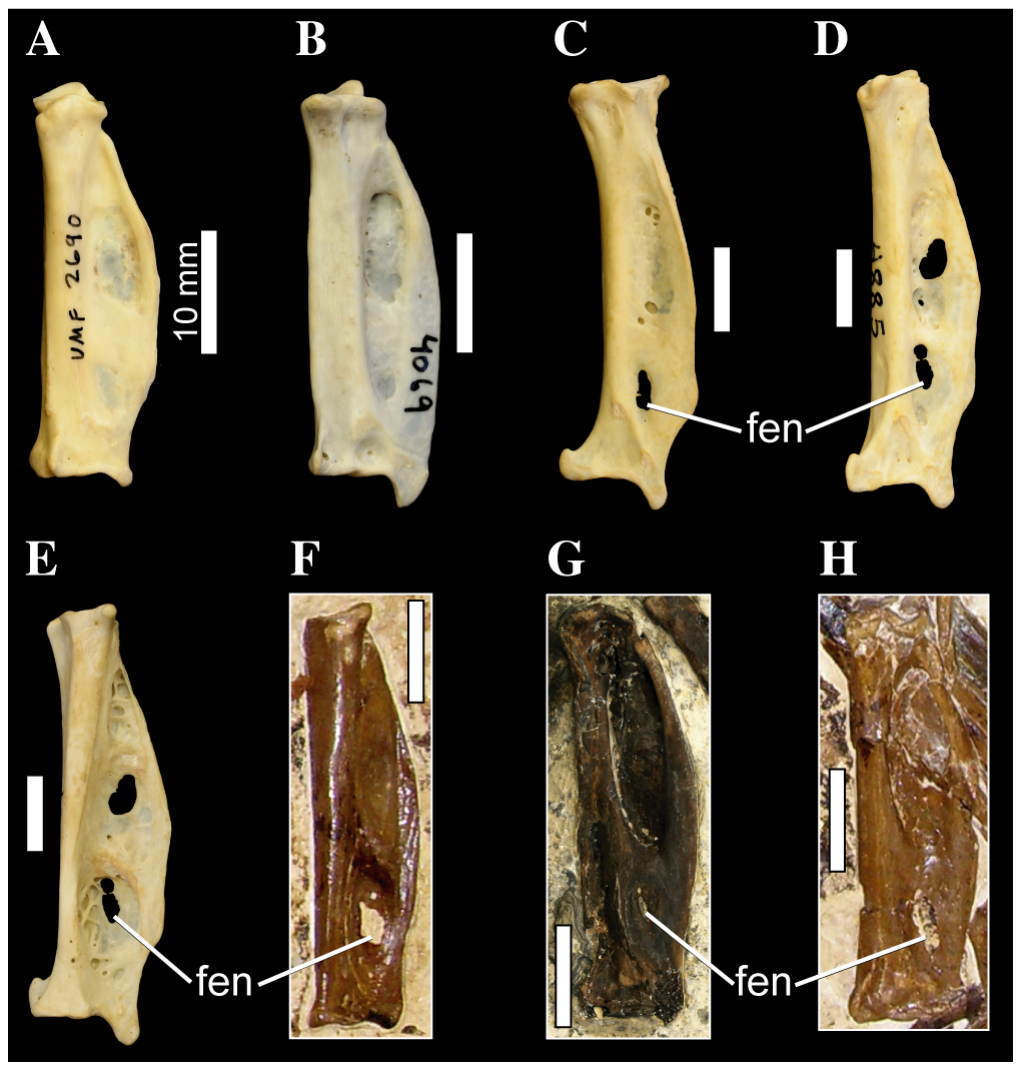
Manual phalanges II-1 of several waterbird taxa. *Ciconia abdimii* FMNH 368771 (A), *Sula sula* FMNH 339372 (B), *Fregata minor* FMNH 339421 (C), *Fregata magnificens* FMNH 339418 (D), *Limnofregata azygosternon* FMNH PA 731 (G), and *Limnofregata azygosternon* USNM 22753 (H), in ventral aspect; and left phalanges II-1 of *Fregata magnificens* FMNH 339418 (right element reversed) (E), and *Limnofregata azygosternon* USNM 22753 (F), in dorsal aspect. Scale bars equal 10 mm. Abbreviations: **fen**, fenestra.

The caudal, blade-like portion of manual phalanx II-1 is typically excavated into two large, proximodistally aligned fossae on its dorsal surface. The excavation on phalanx II-1 created by these fossae can sometimes be extreme enough to make this portion of the phalanx translucent, as in *Scopus*. In most waterbirds however, the distal fossa is not associated with any fenestration of phalanx II-1. In *Fregata*, a large, irregular and proximodistally elongate fenestra perforates the distal portion of the blade of phalanx II-1. As originally noted by Olson ([35]: p. 24) a similar fenestra is also present in the holotype of *Limnofregata azygosternon* (USNM 22753). Though perforation in such a fragile and thin area of the skeleton might easily be attributed to taphonomic or preparation damage in a single fossil specimen, both right and left elements of USNM 22753 preserve the fenestra, as well as portions of its smooth, rounded rims. In addition, several referred specimens (FMNH PA 720, FMNH PA 731; FMNH PA 755) also preserve the distal fenestra (Figure 14). Although the fenestra is slightly smaller in these specimens than in USNM 22753 and modern *Fregata*, the fenestrae in all *Limnofregata* specimens are proximodistally elongate and slightly elliptical. The fact that seven different elements of phalanx II-1 of *Limnofregata* possess a small, proximodistally elongate fenestra on their distal blade argues against the perforation being artifactual.

#### 394. Tibiotarsus, medial ridge of trochlea cartilaginis tibialis hypertrophied, robust, and mound-like: absent (0); present (1). New Character, though see also ([64]: character 2170)

**Figure 15:**
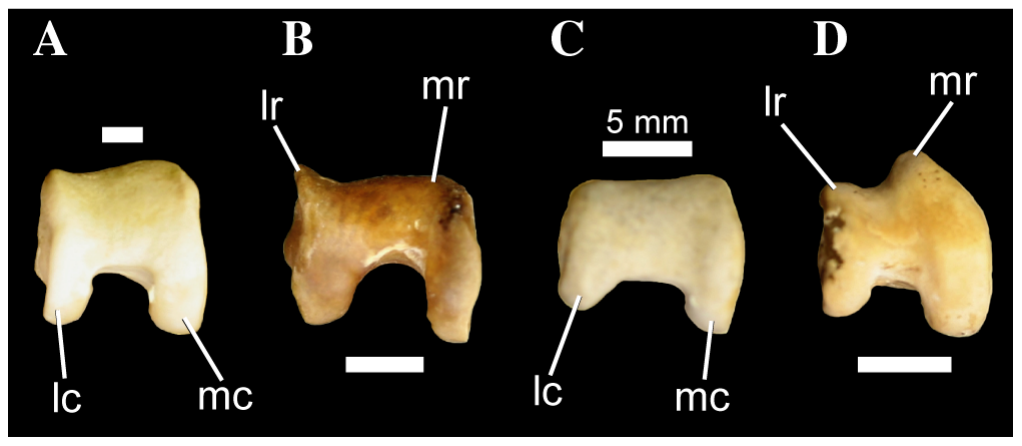
Left tibiae of several pelecaniforms in distal aspect. *Pelecanus erythrorhynchos* FMNH 445082 (A), *Phalacrocorax auritus* FMNH 348372 (B), *Sula sula* FMNH 339372 (C), and *Fregata magnificens* FMNH 339418 (D). Scale bars equal 5 mm. Abbreviations: **lc**, lateral condyle; **lr**, lateral ridge of trochlea cartilaginis tibialis; **mc**, medial condyle; **mr**, medial ridge of trochlea cartilaginis tibialis.

In most waterbirds, the medial ridge of trochlea cartilaginis tibialis on the posterior face of the distal tibiotarsus is not particularly more robustly developed than the lateral ridge. In *Fregata*, however, the medial ridge is mediolaterally widened, mound-like, and much more condylar in appearance than the lateral ridge, particularly at the proximal end of trochlea cartiliaginis tibialis on the posterior tibiotarsus (Figure 15). Olson ([35]: p. 27) originally alluded to the condition in *Limnofregata*, stating: “In posterior view the crest of the internal condyle is better developed than in *Sula* or *Phaethon* and more closely resembles the condition in *Fregata*”. The holotype of *Limnofregata azygosternon* (USNM 22753) includes a complete left tibiotarsus. The proximal third was removed and is contained on a smaller block that includes the sternum and distal third of the left femur. The distal two-thirds of the left tibiotarsus remain on the main block, and is exposed in caudal aspect, with its distal end adjacent to the left pubis and posterior portion of the ilium. The posterior face of trochlea cartilaginis tibialis is clearly visible, and the medial ridge is more robustly developed than the lateral, and condylar in appearance, similar to the condition in modern *Fregata*.

#### 408. Tarsometatarsus, development and orientation of eminentia intercondylaris ( =  “intercotylar prominence”): proximally high and well-developed (0); short, with a distinct spherical proximodorsal projection (1); short, and rounded, weakly developed with no dorsal component (2) ([7]: character TMT2)

In most taxa, eminentia intercondylaris is a robust, well-developed process that projects proximally from between the proximal cotyles of the tarsometatarsus as a rounded triangular eminence. In *Papasula* and *Sula*, this process is much shorter, more spherical, and has a distinct dorsal component to its projection as well. Interestingly, this morphology is also present in the plotopterids *Phocavis* and *Copepteryx hexeris* ([5], [60]; USNM 243773–cast of KMNH VP 200,001), and ?*Borvocarbo stoeffelensis* [54], [55]. In *Fregata*, *Limnofregata*, penguins, and *Morus*, the intercotylar process is not as well developed, and is low and rounded without a distinct dorsal projection.

### Relationships of the *Limnofregata* + *Fregata* Clade

A sister-taxon relationship between the *Limnofregata*/*Fregata* dyad and Suloidea is recovered in the present analysis, and supported by 16 unambiguous synapomorphies, two of which exhibit no homoplasy across the MPTs. Though recent studies of higher-level avian relationships based on molecular data have begun to converge on robust support for a *Fregata* + Suloidea clade [27], [29], this remains slightly incongruent with morphological phylogenetic analyses. Nearly every morphological phylogenetic analysis of pelecaniform relationships recovers *Pelecanus* as sister-taxon to Suloidea, with *Fregata* as the sister-taxon to this larger clade [3], [4], [26]. Notable exceptions to this pattern include the analyses of Mayr [22], which could not resolve the relative position of *Fregata* and *Pelecanus* to Suloidea (though Mayr [22] utilized the traditional topology for his discussion of character optimization); and Mayr [23], which recovered *Fregata* as closer to Suloidea than *Pelecanus*, though a clade formed of Spheniscidae and the extinct Plotopteridae were recovered as the actual sister-taxon to Suloidea. While the ‘switching’ of positions of *Pelecanus* and *Fregata* recovered in this analysis may seem like a relatively minor detail, the fact that the phylogenetic position of *Pelecanus* in the waterbird tree is so contentious, and that these two alternate positions for *Fregata* render the node being temporally calibrated by *Limnofregata* either congruent or incongruent with molecular topologies, merits further discussion of the characters supporting the (*Limnofregata*/*Fregata*) + Suloidea clade in the present analysis.

#### 38. Quadrate, intercondylar sulcus of mandibular process a deep, parabolic (“U-shaped”) channel with sides (craniocaudal perspective) subdiagonal (typically parallel) and directly opposite each other and dorsally foveate (ventral perspective): absent (0): present (1) ([64]: character 529)

The derived condition is present in Suloidea, *Fregata*, *Limnofregata*, and also convergently present in herons. Livezey and Zusi ([64]: character 529) considered the derived condition to also be present in *Pelecanus*. However, the U-shaped channel of the above-mentioned taxa is much more deeply excavated, and also more extensive craniocaudally than in *Pelecanus*, which more closely resembles the morphology of *Balaeniceps*.

#### 67. Dorsal tympanic recess, rostroventral position relative to quadrate cotyles: caudal to intermediate between cotyles (0); main portion of recess situated rostral/rostromedial to cotyles (1) ([23]: character 16); see also ([26]: character 31) and ([64]: character 223)

In most waterbirds, the main portion of ventral opening of the dorsal tympanic recess is situated in between, or caudal to, the quadrate cotyles. However, in a variety of taxa, including Sphenisciformes, some procellariforms, Phaethontidae, Prophaethontidae, *Fregata*, *Limnofregata*, and Suloidea, this primary opening for this recess is located rostral, or rostromedial, to the quadrate cotyles (Figure 7). Though Livezey and Zusi ([64]: character 223) considered the derived condition to also be present in *Pelecanus*, the main opening of the dorsal tympanic recess is primarily between the quadrate cotyles in this taxon (Figure 7), and very similar to the position present in *Ciconia*. *Pelecanus* is additionally unique in that a portion of the dorsal tympanic recess opens caudolateral to the quadrate cotyles (Figure 7; [26]: character 31).

#### Character 119. Sternum, angle of long axis of craniolateral process relative to midline of sternum: perpendicular, ∼90 degrees (0); diagonal, ∼45 degrees (1), parallel, ∼0 degrees (2) ([64]: character 1142)

In most waterbirds, and indeed most avian taxa, the craniolateral process extends straight laterally from the edge of the sternum, at an angle near perpendicular to the midline. In *Gallus*, and most galliformes, the angle of the craniolateral process is near parallel to the midline. In *Balaeniceps*, *Fregata*, Sulidae, *Anhinga*, and *Phalacrocorax*, these processes extend craniolaterally from the edge of the sternum, at an angle closer to 45 degrees (Figure 12). Note also that I disagree with Livezey and Zusi ([64]: character 1142) regarding the condition in *Pelecanus*, which I interpreted as possessing the plesiomorphic state (Figure 12). The holotype of *Limnofregata azygosternon* (USNM 22753) contains a nearly complete sternum preserved in left ventrolateral view on a small block that is counterpart to the main slab. The left craniolateral process is preserved an angled approximately 45 degrees from the midline, as in *Fregata* and Suloidea. An additional specimen, FMNH PA 755, also includes a near-complete sternum, oriented in dorsal aspect. Only the right craniolateral process is preserved, and its cranial tip is missing, but it clearly projects craniolaterally from the edge of the sternum at an angle near 45 degrees (Figure 12). The fact that *Balaeniceps* also possesses state “1” renders this a synapomorphy of a *Fregata* + Suloidea clade under under DELTRAN character optimization.

#### 217. Humerus, distinct ridge extending significantly proximally up humeral shaft from proximal end of tuberculum supracondylare ventrale: absent (0); present (1) ([33]: character 83)

In *Fregata*, *Limnofregata*, Sulidae, and *Phalacrocorax*, a distinct raised ridge is present at the proximal end of tuberculum supracondylare ventrale. This low ridge extends proximally up the shaft of the humerus. *Anhinga* represents a reversal to the plesiomorphic state.

#### 275. Ossa metacarpalia, relative distal extent of metacarpals II and III: metacarpal II equal to or longer than metacarpal III (0); metacarpal II shorter than metacarpal III (1) ([64]: character 1591)

Among waterbirds, a metacarpal II that is distinctively shorter in distal extent than metacarpal III is present in Sphenisciformes, Ardeidae, Threskiornithidae, *Ciconia*, *Scopus*, *Balaeniceps*, and *Pelecanus*. *Fregata*, Suloidea, and the extinct plotopterid *Tonsala hildegarde* (USNM 256518; [50]: Figure 3) all possess a metacarpal II that is as long or longer than metacarpal III distally. State “0” is also present in *Gavia*, *Podiceps*, Procellariiformes, *Phaethon*, and *Phoenicopterus*.

#### 330. Pubis, middle of pubic shaft distinctly reduced in diameter relative to proximal and distal portions: absent (0); present (1) ([64]: character 1932)

In most waterbirds, the pubic shaft is relatively consistent in diameter throughout most of its length. However, in *Fregata*, *Anhinga*, *Phalacrocorax*, and Sulidae, the middle portion of the pubic shaft is extremely constricted in diameter relative to the proximal and distal portions. The area of this constriction is typically at the level of pubic shaft ventral to the ilioischiadic foramen. Olson ([35]: p. 15) noted that: “In *Limnofregata* the width of the pubis is about the same throughout its length”, and though only the left pubis of the holotype of *Limnofregata azygosternon* (USNM 22753) is complete, this does appear to be the case. Two other specimens of *Limnofregata* (FMNH PA 755; WSGS U1-2001) also exhibit well-preserved pubes, and though these elements have been flattened slightly and do not retain their original 3-dimensional morphology, the shafts of the pubes do appear to be relatively uniform in thickness throughout. The plesiomorphic state also appears to be present in BMS E 25336 ([59]: Figure 7). Thus, the exact distribution of the derived character state is uncertain at present, and represents either a synapomorphy of a *Fregata* + Suloidea clade (reversed in *Limnofregata*), or two cases of independent acquisition in modern frigatebirds and suloids.

#### 332. Pubis, distinct ventral “kink” in pubic shaft near terminal end, resulting in an oblique angle formed between the caudoventrally directed apex pubis and the body of the pubis in lateral aspect: absent (0); present (1) ([64]: character 1945)

In most waterbirds there is no marked inflection in the shaft of the pubis near its terminal end. In *Fregata*, Sulidae, *Anhinga*, and *Phalacrocorax*, the distal portion of the pubic shaft has a distinct ventral ‘kink’ at the level where the reduced middle portion of the shaft (see character 330 above) meets the more robust distal portion. The ‘kink’ occurs along the pubis near the level of the terminal end of the ischium. This ventral inflection produces a distinct oblique ventral angle between the distal apex of the pubis and the middle portion of the pubic shaft, and also creates a low, triangular crest on the dorsal margin of the pubic shaft. Though several specimens of *Limnofregata* include well-preserved pelves and associated pubes (e.g., USNM 22753; FMNH PA 723; FMNH PA 755; WSGS U1-2001), all of these are preserved in ventral aspect and have been subject to some degree of dorsoventral compression, making it impossible to assess any degree of inflection in the distal pubic shaft. This character can also not be assessed for BMS E 25336 ([59]: Figure 7), which includes a pelvis preserved in dorsal aspect.

#### 341. Femur, development of caudal margin of facies articularis antitrochanterica: low, linear caudal edge (0); robust edge, distinctly everted caudally and extending beyond facies caudalis (1) ([64]: character 1975)

In most waterbirds, facies articularis antitrochanterica is confined to the proximal portion of the femur, and its caudal edge is linear. In *Fregata*, Sulidae, and *Anhinga*, the caudal portion of this articular facies is robustly developed, such that it extends slightly further caudally, and is everted slightly distally beyond facies caudalis. This results in the caudal portion of facies articularis antitrochanterica forming a distinct ‘lip’ on the caudal side of the proximal femur. This condition is particularly accentuated in *Fregata*. An extremely well preserved specimen of *Limnofregata hasegawai* (WSGS U1-2001) includes both right and left femora exposed in caudal aspect. Though the proximal portion of the right element is slightly better preserved, both femora exhibit a strong caudal expansion of facies articularis antitrochanterica and the associated everted ‘lip’, and are very similar to the morphology present in *Fregata*. The caudal portion of facies articularis antitrochanterica is slightly damaged in one of the paratype specimens of *Copepteryx hexeris* (USNM 243774–cast of NSMT VP 15035; [5]), though a caudally everted ‘lip’ appears to be present in another paratype (USNM 243773–cast of KMNH VP 200,001). Additionally, the derived state is clearly present in *Copepteryx titan* (USNM 486685–cast of KMNH VP 200,004; [5]), and multiple femora of various sizes referred to *Copepteryx* from the same Formation (USNM casts; [5]). Note that the current evaluation of this character departs considerably from the original construction of this character and the associated character state distribution of Livezey and Zusi ([64]: character 1975), who considered the derived condition to be present in *Lithornis*, *Eudromia*, and Galloanserae.

#### 401. Fibula, distinct caudodistally trending sulcus on craniolateral border of fibula: absent (0); present (1). New Character

In most waterbird taxa, the craniolateral edge of the fibula below the expanded fibular head is relatively smooth and unmarked. However, in a variety of taxa, including *Gavia*, *Fregata*, Sulidae, *Phalacrocorax*, and *Anhinga*, a distinct sulcus is present in this area. The sulcus begins just distal to the cranial edge of the fibular head and extends distally and curls caudally across the lateral surface of the fibula, though it remains well proximal to the tubercle for insertion of m. iliofibularis. The cranial edge of the sulcus is typically better defined and ridge-like than the caudal border. In several taxa where the craniolateral tubercle on the fibular head is distinct (e.g., *Sula*, *Morus*), the proximal portion of this sulcus actually extends across and carves a groove in the craniolateral tubercle. Though several specimens of *Limnofregata* contain complete or partial fibulae (e.g., USNM 22753; FMNH PA 723; FMNH PA 755; WSGS U1-2001), none of these elements are well preserved enough, or oriented in the correct direction, to allow assessment of this character.

#### 413. Tarsometatarsus, relative sizes of articular facets of proximal cotyles: subequal (0); medial cotyle distinctly more expansive than lateral cotyle (1) ([64]: character 2251); ([7]: character TMT3)

In most waterbirds, the articular areas of the medial and proximal cotyles of the tarsometatarsus are approximately the same size. However, in *Fregata*, Sulidae, *Anhinga*, and *Phalacrocorax*, the medial cotyle is significantly larger than the lateral cotyle. This condition is also present in the plotopterids *Copepteryx hexeris* [5], and *Phocavis* ([60]: p. 98). Both tarsometatarsi of the holotype of *Limnofregata azygosternon* (USNM 22753) are preserved, though the proximal cotyles of the left element are obscured by matrix. The articular cotyles of the right element are visible however, though the right tibiotarsus partially covers the posterior portion of the lateral cotyle. Despite this, it is clear that the medial cotyle is relatively larger than the lateral cotyle. Both tarsometatarsi of a specimen of *Limnofregata hasegawai* (WSGS U1-2001) are also preserved in anterior aspect, however they are still in articulation with their respective tibiotarsi, such that the relative areas of their proximal cotyles cannot be confidently assessed.

#### 445. Tarsometatarsus, relative distal extents of trochleae metatarsals: II < III > IV, and II subequal to IV (0); II < III > IV, and II much less than IV (1); II < III &ge ; IV, and II > IV (2); II > III ≥ IV (3) ([64]: character 2361); ([23]: character 49); ([7]: character TMT9)

Among waterbirds, *Fregata*, *Limnofregata*, Sulidae, *Anhinga*, ?*Borvocarbo stoeffelensis*, and ‘microcormorants’ are unique in possessing trochleae metatarsal II that extend further distally than trochleae metatarsal III (i.e., state “3”). Note however, that two reversals to state “2” are inferred within the *Fregata* + Suloidea clade in the MPTs: one on the lineage leading to Plotopteridae, and one on the lineage leading to non-‘microcormorant’ *Phalacrocorax*.

## Discussion

### Pelecaniform Fossil Calibration Points and Implications for Temporal Patterns of Diversification

Both *Prophaethon* [95], and more recently *Lithoptila* [27], [28], have been utilized as fossil calibration points for analyses of divergence timing in higher-level avian clades. Of these two taxa, *Lithoptila* represents the oldest record of Prophaethontidae and stem-group tropicbirds, with the oldest specimens recovered from upper Paleocene (Thanetian) sediments [26], [53], though *Prophaethon*, from the lower Eocene (Ypresian) London Clay, is only slightly younger [61]. The only previously published phylogenetic analysis to include members of Prophaethontidae [26] also recovered strong support for the recovery of both *Prophaethon* and *Lithoptila* as stem tropicbirds. Thus, at present, the use of either of these taxa as a calibration point for the node uniting Phaethontidae with its closest extant relative appears to be well justified.

However, considerable disagreement exists regarding the closest extant relative of Phaethontidae. Recent analyses of morphological data have suggested that Phaethontidae are sister taxon to Procellariiformes [25], [26], (the present study), or to the remaining members of a traditional Pelecaniformes [4]. Several recent higher-level molecular studies sampling nuclear genes suggest that Phaethontidae is a member of an ecologically diverse clade of neoavians variably referred to as “Metaves” [19], [27], [29]. However, the relative relationships of tropicbirds within this clade are not particularly well supported or congruent across these studies. Additionally, a recent mitogenomic study of higher-level avian relationships recovered Spheniscidae as the sister taxon to Phaethontidae, with this larger clade as the sister taxon to a (Fregatidae + Sulidae + Phalacrocoracidae) clade [28]. Accordingly, though the phylogenetic placement of *Prophaethon* and *Lithoptila*, and their use as a calibration point for the divergence of tropicbirds from their closest extant relative may be well-supported, the relative impact this calibration point has on patterns of temporal divergence in higher-level avian phylogeny will be dependent on the successive extant sister group relationships of modern tropicbirds, which remain extremely labile at present.

Given the topology recovered in the present analysis (Figure 2), a late Paleocene (Thanetian) age for *Lithoptila* implies that the lineages leading to Procellariiformes and to the loon + grebe + penguin clade had both diverged prior to the Eocene. This is largely consistent with the fossil record of the Procellariiformes. The oldest fossil material tentatively assigned to this clade is the nearly complete right humerus of *Tytthostonyx glauconiticus* from the Late Cretaceous or early Paleocene of New Jersey [96]. However, Olson and Parris ([96]: p. 16) stressed the tentativeness of a referral to Procellariformes, noting similarities between *Tytthostonyx* and Charadriformes and Pelecaniformes. Similarly, Bourdon et al. ([53]: p. 759) noted similarities between *Tytthostonyx* and *Lithoptila*, as well as Charadriformes, and rejected procellariform affinities for this taxon. As De Pietri et al. ([97]: p. 1) note, fragmentary Eocene fossils from throughout the world have been referred to extant procellariform families [98]–[100]; and more complete stem procellariforms are known from the early Oligocene [97], [101]. The future analysis of this material in a rigorous phylogenetic framework would allow more definitive statements regarding the earliest records of stem and crown Procellariiformes.

Similarly, the oldest purported members of the Gaviiformes are known from the Cretaceous, and include *Neogaeornis wetzeli*, an isolated tarsometatarsus from Campanian/Maastrichtian sediments of Chile [102]; and *Polarornis gregorii*, a partially associated skeleton from the Late Cretaceous of Antarctica [103]. However, doubts have been raised regarding the gaviiform affinities of both of these taxa [104], [105]. The late Eocene *Colymboides anglicus* [106], [107], consisting of a coracoid (and subsequently a referred humerus and frontal portion of a skull; [108]) constitutes the next oldest fossil material referred to Gaviiformes, though more informative, partially associated skeletons are not known until the early Oligocene [104], [109].

The oldest stem penguin fossils include several partial skeletons from the Paleocene of Antarctica [110] and New Zealand [111]. Of these, *Waimanu manneringi* represents the oldest known stem penguin, dated with calcareous nannofossils as late early Paleocene (60.5–61.6 Myr; [111]: p. 1145, suppl. mat.). The relationships of *Waimanu manneringi*, and its slightly younger sister taxon *Waimanu tuatahi*, have also been evaluated in several phylogenetic analyses of morphological, and combined molecular and morphological datasets, and are consistently recovered as the basal-most members of Sphenisciformes [85], [111], [112]. Given that relatively complete, associated skeletons of *Waimanu* have been recovered, the temporal data associated with this fossil material is well constrained, and the phylogenetic relationships of *Waimanu* have been rigorously tested, this material represents a key calibration point for use in studies of higher-level avian diversification.

*Limnofregata* has likewise been utilized as a fossil calibration point for analyses of divergence timing in recent studies [27], [28]. Though *Limnofregata* has long been considered as closely related to extant frigatebirds [23], [35], [36], [51], [59], [113], the present study represents the first time this taxon has been evaluated in a modern phylogenetic analysis. *Limnofregata* is robustly supported as the sister taxon to modern frigatebirds in the present analysis (Figure 2). The characters supporting this clade are distributed throughout the entire avian skeleton, which argues against a close relationship being erroneously inferred due to ecomorphological convergence in a particular anatomical subregion [114]. In addition, many of the characters supporting a *Limnofregata*/*Fregata* clade are preserved in multiple specimens of *Limnofregata*, which provides confidence that character states have been accurately assessed, and cannot be attributed to taphononomic or preparation damage of an individual specimen (e.g., the distal fenestration of manual phalanx II-1). This robust phylogenetic placement, coupled with the detailed age control on the the early Eocene Fossil Butte Member of the Green River Formation (from which the majority of *Limnofregata* specimens are known), which recent radiometric dating indicates is 51.97+/−0.16 Myr [58], makes *Limnofregata* particularly attractive as a potential fossil calibration point for future studies of higher-level avian divergence timing.

The present study also represents some of the first support from morphological phylogenetic analysis for a sister taxon relationship between Fregatidae and Suloidea. Additional evidence supporting the monophyly of a *Fregata* + Suloidea clade includes the unique synovial intraramal joints in the mandibles of these taxa ([115]; [64]: character 712; though see also Louchart et al. [116], who consider the joint to be present in *Pelecanus*). Most previous analyses of morphological data recover *Pelecanus*, and then *Fregata*, as successive sister-taxa to Suloidea [3], [4], [26]. Morphological support for a sister taxon relationship between Fregatidae and Suloidea is noteworthy, as a growing body of evidence from molecular phylogentic analyses of higher-level avian relationships also recovers this clade, often with strong support [19], [27]–[29]. Thus, there is somewhat of a provisional consensus across morphological and molecular studies on the extant sister group of Fregatidae, and accordingly the node being calibrated by *Limnofregata* fossils. An important caveat however, is that relative waterbird relationships outside of this *Fregata* + Suloidea clade still remain largely incongruent between morphological and molecular analyses (Figure 1), and recent studies [4], [19], [23], [26]–[29] often do not recover strong statistical support for these higher-level clades. Thus, the relative impact that a temporal calibration from *Limnofregata* would provide will be highly topology dependant, and involves which clades represent successive sister taxa to the *Fregata* + Suloidea clade.

Given the topology recovered in the present analysis (Figure 2), *Limnofregata* represents the earliest representative of Steganopodes, and suggests that the lineages leading to Fregatidae and Suloidea diverged by the early Eocene. This is largely in agreement with the fossil record of Suloidea, particularly if plotopterids belong within this clade (see below). As previously noted, this implies an enormous gap in the fossil record of Fregatidae, which, with the exception of *Limnofregata*, includes only Quaternary fossils from oceanic islands [36], [59]. In addition, the age of *Limnofregata* and the paraphyletic arrangement of *Scopus*, *Balaeniceps*, and *Pelecanus* to the *Fregata* + Suloidea clade recovered in the present analyis (Figure 2), suggest that the lineages leading to the former three taxa also diverged by the early Eocene. This again implies a large amount of missing fossil record for these three groups, as the Scopidae includes only one fossil taxon, *Scopus xenopus*, known from a distal tarsometatarsus and partial coracoid from the early Pliocene of South Africa [36], [117]; the Balaenicipitidae is known from only a few more fossil occurrences [36], the oldest of which is *Goliathia andrewsi* from the early Oligocene Jebel Qatrani Formation in Egypt [118]; and the fossil record of Pelecanidae, while slightly more extensive than that of Scopidae or Balaenicipitidae, reliably extends only to the early Oligocene of France [36], [90], [116]. This anomaly could stem from many factors, including the possibility that these lineages were represented by species-poor clades throughout their histories (e.g., both Scopidae and Balaenicipitidae are monotypic families, and the approximately seven species of extant *Pelecanus* are generally considered to represent recent divergences), or that much of these lineages' biogeographic history took place in areas that have poor fossil records, or have not been well-sampled (e.g., the current distributions of Scopidae and Balaenicipitidae are much more restricted than many other, more cosmopolitan, waterbird families). Alternatively, the phylogenetic position of these taxa recovered in the current analysis may be incorrect. As noted earlier, a growing body of evidence from molecular studies supports the monophyly of a clade including *Pelecanus*, *Balaeniceps*, and *Scopus* [19], [20], [24], [27], [29], and divergence dates recovered from molecular clock studies utilizing these topologies are in closer accord with the known fossil records for these three lineages ([27]: Figure 2). The divergence times recovered by Ericson et al. ([27]: Figure ESM-9) for the split between *Pelecanus* and its closest relative varied between ∼30 Myr for their PATHd8 analysis and ∼50 Myr for their Penalized Likelihood analysis. The former of which is more closely in accord with the earliest fossil record of the Pelecanidae, a nearly complete skull and neck from Rupelian strata (28.25–33.00 Myr) of southeastern France [116].

The majority of plotopterid specimens described are from late Oligocene and Miocene deposits [43], [49], [50], [59]. The oldest member of the family is *Phocavis maritimus*, from the Late Eocene to Early Oligocene Keasey Formation of the Pacific northwestern United States [60], [119]. Establishing the exact age of the Keasey Formation has been problematic ([119]: p. 16474). When Goedert ([60]: p. 99) originally described *Phocavis*, which is from the upper part of the informal middle member of the Keasey Formation, he considered the Keasey Formation to be Late Eocene, correlated with the Priabonian Chronostratigraphic Stage (40–36.6 Myr; [120]). This correlation was followed by Warheit [37], though Warheit ([38]: Appendix 2.1) placed *Phocavis* in the middle Eocene, with no clear justification. However, Prothero and Hankins [119] subsequently revised the age of the Keasey Formation utilizing magnetic stratigraphy, and suggested that the middle portion of the Keasey Formation was correlative with Chron C13r (33.5–34.6 Myr), which spans the Eocene–Oligocene boundary. In addition to *Phocavis*, Olson and Hasegawa ([5]: p. 742) briefly mention “as many as six species” of plotopterids in Japan from “older beds that are probably early Oligocene (but possibly late Eocene)”, though these specimens remain to be described in detail.

Given the phylogenetic position of Plotopteridae (Figure 2), this suggests that the lineages leading to Sulidae and to Phalacrocoracoidea had diverged by the Eocene–Oligocene boundary. This is largely consistent with the fossil record of Sulidae, which includes possible stem members such as *Eostega lebedinskyi*, from the middle Eocene of Romania [52], [121], *Masillastega rectirostris*, from the middle Eocene of Messel, Germany [51] (though see also Mlíkovsky [52], who refers this species to *Eostega*), and *Sula ronzoni* from the early Oligocene of France [36]. However, the relationships of these taxa remain to be tested in a rigorous modern phylogenetic analysis. The earliest definitive record of Phalacrocoracoidea is not known until the late Oligocene (∼24.7 Myr), with the presence of the stem phalacrocoracid ?*Borvocarbo stoeffelensis* from the Enspel fossil site in Germany ([55]; Figure 2; see also below). Fossil members of the Anhingidae are not known until the early Miocene [122]. Potential older members of Phalacrocoracoidea are known, including undescribed material referred to Phalacrocoracidae from the Eocene–Oligocene Phosphorites du Quercy, France [36], [123], and a partial premaxilla referred to Phalacrocoracidae from the early Oligocene Jebel Qatrani Formation in Egypt [118]. In addition, the enigmatic *Plotoplotus beauforti* from the Paleocene (see [124]) of Sumatra has been considered to exhibit affinities with pelecaniforms, particularly Anhingidae [36], [40], [42]. Once again, the relationships of taxa representing these purported earlier occurrences remain to be rigorously evaluated in a phylogenetic framework. Also, given the uncertainty surrounding the precise phylogenetic placement of Plotopteridae [5], [23], [50] (Figure 2), and the fact that a sister taxon relationship between Plotopteridae and penguins (as advocated by Mayr [23]) is only slightly less parsimonious in the present dataset, extreme caution should be excerised in using any member of the Plotopteridae as a potential fossil calibration point for divergence time studies.

A partial right foot, tarsometatarsus and distal tibiotarsus referable to ?*Borvocarbo stoeffelensis* (this material was originally considered ?*Oligocorax* sp. by Mayr [54]; Mayr [55] later described a new, nearly complete specimen as ?*Borvocarbo stoeffelensis* and referred this material to the new taxon) has also been utilized in one analysis of avian divergence timing [27]. This specimen was employed as a calibration of stem Phalacrocoracidae, and hence, the Anhingidae/Phalacrocoracidae split. Recovered from the late Oligocene fossil site of Enspel, dated at roughly 24.7 Myr [55], [125], ?*Borvocarbo stoeffelensis* represents the most complete and oldest fossil that can be referred to stem Phalacrocoracidae, or stem Phalacrocoracoidea (Anhingidae + Phalacrocoracidae). However, the present study represents the first time ?*Borvocarbo stoeffelensis* has been included in a morphological phylogenetic analysis, where it is recovered as the sister taxon to all other included Phalacrocoracidae (Figure 2).

Mayr ([55]: p. 940) cautioned against a straightforward assignment of ?*Borvocarbo stoeffelensis* to stem Phalacrocoracidae, based on the presence of two characters interpreted as plesiomorphic relative to Phalacrocoracoidea: **1)** paroccipital processes that are less pointed and caudally everted; and **2)** a shorter bicipital crest that meets the humeral shaft at a steeper angle. The present analysis recovered the first character as a synapomorphy of Suloidea (character 66). However, due to the potential distortion of the rear of the skull of ?*Borvocarbo stoeffelensis*, this taxon was coded as “?”, or missing data, for this character in the present analysis. The second character listed by Mayr ([55]: p. 940) was not included in this analysis. However, two additional characters that Mayr ([55]: pp. 939–940) listed as potentially uniting ?*Borvocarbo stoeffelensis* and Phalacrocoracidae were also not included in the present analysis: **1)** a well-developed crista nuchalis sagittalis along the midline of the skull, which is present in Phalacrocoracidae with the exception of the ‘microcormorants’ (see also [33]: character 3); and **2)** an accessory transverse cranial crest present caudal to crista nuchalis transversa. Thus, it is likely that inclusion of these additional characters would only increase the support for the recovery of ?*Borvocarbo stoeffelensis* as a stem Phalacrocoracidae. Despite this, the treatment of ?*Borvocarbo stoeffelensis* as a fossil calibration point for stem Phalacrocoracidae should still be viewed with some caution. If, as suggested by Mayr [55], [63], many of the characters supporting a close relationship between ?*Borvocarbo stoeffelensis* and Phalacrocoracidae actually represent secondary reversals in Anhingidae, then a ‘cormorant-like’ morphology would be expected for fossil taxa spanning the stem of both Phalacrocoracoidea and Phalacrocoracidae.

### Relationships of the Plotopteridae

The recovery of a monophyletic clade of extinct Plotopteridae as sister-taxon to the Phalacrocoracoidea suggests that wing-propelled diving evolved independently in Plotopterids and penguins, contra Mayr [23]. This study thus confirms previous work [43], [59], [50], which suggested that the skeletal anatomy of plotopterids represents a remarkable case of convergent evolution on the more familiar penguin body-plan. However, despite this exceptional degree of morphological convergence, the analysis of osteological characters in a modern phylogenetic framework is able to parse out the numerous similarities in the penguin and plotopterid pectoral girdle and appendages as the result of homoplasy (e.g., characters 107, 159, 164, 165, 198, 210, 220, 229, 248, 256). An important caveat however, is that members of Spheniscidae were relatively sparsely sampled for the current analysis (two taxa), and no fossil sphenisciforms, particularly along the stem leading to extant penguins, were included. As noted above, the character state distributions present in some fossil penguins may in some cases increase (e.g., character 272), or decrease (e.g., character 427), support for a close relationship between penguins and plotopterids. Furthermore, enforcing a monophyletic clade of Spheniscidae + Plotopteridae results in trees that are only four steps longer than the MPTs from the unconstrained analysis of the full dataset.

An additional pattern is present in the dataset that lends support to the phylogenetic position of plotopterids recovered in the current analysis. Although there are numerous characters (particularly in the pectoral girdle) shared by plototperids and penguins (see above discussion), there appear to be few, if any synapomorphies that diagnose the nodes subtending the penguin clade (i.e., those that include loons, grebes, procellariforms, and tropicbirds) that are present in plotopterids. On the contrary, there are character states present in plotopterids that represent synapomorphies of higher-level clades within Steganopodes. For example, three unambiguous synapomorphies of the *Fregata* + Suloidea clade (175:1–>2; 341:0–>1; 413:0–>1) are present in one or more plotopterid. Furthermore, three unambiguous synapomorphies that diagnose Steganopodes (169:0–>1; 395:0–>1; 440:0–>1) are also in one or more plotopterid. Three unambiguous synapomorphies diagnosing the *Balaeniceps* + Steganopodes clade (155:0–>1; 187:0–>1; 434:0–>1) are also present in one or more plotopterid. Finally, one unambiguous synapomorphy diagnosing the *Scopus* + *Balaeniceps* + Steganopodes clade (143:0–>1) is present in all three plotopterids for which this character can be scored. These synapomorphies are also distributed throughout the entire skeleton, and not concentrated in a particular anatomical region. If the phylogenetic position of plotopterids recovered in the present analysis (Figure 2) is correct, than this sort of pattern of hierarchically nested synapomorphies present in the skeletons of plotopterids is to be expected, while morphological convergence related to the evolution of wing-propelled diving would result in plotopterids sharing character states with penguins, but not with the larger clades (e.g., those that include loons and grebes, or loons, grebes, procellariforms, and tropicbirds) that penguins are nested within. Additional comparative anatomical work on fossil penguins and plotopterids, coupled with expanded taxon sampling and more taxon-specific character sampling will likely yield further insight into the phylogenetic relationships of Plotopteridae.

### Partitioned Analyses and Implications for Patterns of Character Evolution

Pairwise ILD tests revealed that the pectoral and pelvic anatomical partitions might possess discrepant phylogenetic signals. However, these results should be interpreted with caution, as the ILD test is a rather coarse tool for examining dataset incongruence, and previous workers have noted the potential for increased type 2 error (falsely rejecting the null hypothesis of dataset congruence) when: many invariant and/or parsimony uninformative characters are included [126], [127]; the level of homoplasious characters varies between partitions [128]; the size of the partitions being analyzed varies greatly [129], [130]; or character partitions are evolving at different rates [130]. Thus the interpretation of detecting significant incongruence between the pectoral and pelvic partitions is not straightforward, and could be an artifact (e.g., related to the relative sizes of these partitions), or the result of other biologically interesting phenomena (e.g., the possibility of different levels of homoplasy or rates of evolution in disparate partitions).

The results of the partitioned phylogenetic analyses also reveal that much of the discrepancy between partitions is localized outside of Steganopodes, and can primarily be attributed to a few problematic taxa (e.g., flamingos, tropicbirds, hammerkop). Furthermore, the nodes that conflict between the MPTs of the partitioned analyses are for the most part not well supported in any of the individual partitioned analyses (Figures 4–6), or the analysis of the full dataset (Figure 2). In addition to documenting the phylogenetic lability of several taxa (e.g., flamingos, tropicbirds, hammerkop), these partitioned analyses also aid in revealing signals that are not at first apparent in the full dataset analysis (Figure 2). These include the fact that the phylogenetic signal supporting the traditional relationship of a *Pelecanus* + Suloidea clade to the exclusion of *Fregata* (in the extant taxa only analyses) appears to be the result of cumulative hidden support across the three major anatomical partitions [80]. An additional interesting pattern recovered from the partitioned phylogenetic analyses is that the strong sister taxon relationship between loons and grebes is supported primarily by characters of the pelvic girdle (Figures 4–6). Partitioned analyses also reveal that pelvic characters alone are not sufficient to recover a monophyletic Sulidae. Gaining a better understanding of the patterns of phylogenetic support present in different anatomical regions could potentially go a long way toward evaluating the confidence that can be placed in the phylogenetic affinities of more fragmentary fossil waterbird specimens.

### Congruence and Conflict in Higher-level Waterbird Phylogeny

As noted in the introduction, higher-level avian phylogeny is far from robustly resolved, and the relationships of Pelecaniformes in particular remain contentious. Given this uncertainty, a brief discussion of the patterns of congruence and conflict between the present study and the most recent and exhaustive morphological [4] and molecular [29] analyses of higher-level waterbird phylogeny is warranted, as well as a consideration of several potential explanations for conflicting topologies.

Rigorously assessing patterns of congruence and conflict between datasets can be problematic in and of itself [131]. While agreement or disagreement in optimal topologies from different datasets can be taken as congruence or incongruence per se, these patterns can also be the result of idiosyncrancies in the datasets, due to differential taxon or character sampling, choice of outgroup/s, or even different methods of analysis [132]–[135]. Additionally, conflict between datasets can often be overestimated if the issue of clade support is not taken into consideration [135].

For the purposes of this discussion, bootstrap values ≥70% are treated as strong support for a clade in a given analysis. This is a pragmatic, but arbitrary cutoff, and there are obvious caveats to this criterion, including the facts that support values are not directly comparable across different analyses, bootstrap values for the studies discussed were calculated under different optimality criterion (maximum likelihood in [29] and maximum parsimony in [4] and the present study), and the bootstrap in general tends to be conservatively biased [78], [136]. Similar methods and thresholds have been used previously for discussing support in empirical studies. For example, Mayr et al. [137] utilized a cutoff of bootstrap support >60% in a comparison of phylogenetic datasets of ‘higher land birds’, Smith et al. [138] employed a threshold of >70% bootstrap support to identify strongly supported conflicting nodes in molecular and morphological estimates of echinoid phylogeny, and Wiens et al. [139] utilized bootstrap values ≥70% as their threshold for designating strong support in a combined analysis of snake phylogeny. Given this criterion for assessing strong support, “congruence” would represent cases where a particular clade is strongly supported in two or more different datasets, while “conflict” would represent cases where a taxon or clade is strongly supported in different positions in two or more datasets. Bootstrap support values were taken directly from Livezey and Zusi ([4]: Figure 14), and Hackett et al. ([29]: Figure 2). For the present study, bootstrap values from the analyis including extant taxa only (Figure 3) were utilized. When applicable, comparisons to other recent studies, as well as the additional analyses of the present dataset (Figures 2, 4 – 6), are drawn as well.

Employing this strategy for comparing the present dataset and those of Livezey and Zusi [4] and Hackett et al. [29] yields a variety of cases of congruence. To begin with, the monophyly of two relatively uncontested waterbird clades, Ardeidae and Procellariiformes, is strongly supported by all three datasets. However, only Livezey and Zusi [4] included a relatively diverse sampling of the Ardeidae, with five different genera sampled from a family of approximately 64 species. Both the present dataset and that of Hackett et al. [29] included only two herons, which allows a relatively limited test of group monophyly. Similarly, all three datasets recovered the clade Procellariiformes with 100% boostrap support, though they each sampled only five taxa from all four procellariform families. This represents a phylogenetically broad, yet sparse, sample of the approximately 110 living species of this avian order. The current analysis and that of Livezey and Zusi [4] are both congruent in recovering a sister taxon relationship between *Phoebastria* (“*Diomedea*” in [4]) and *Puffinus*, though this does not imply a sister taxon relationship between Diomedeidae and Procellariidae, as the present dataset only sampled one member of the each family, and Livezey and Zusi [4] recovered their two sampled members of Procellariidae, *Puffinus* and *Pachyptila*, as successive sister taxa to *Phoebastria* (“*Diomedea*” in [4]). Both the Livezey and Zusi [4] and Hackett et al. [29] analyses recover strong support for a sister taxon relationship between Sphenisciformes and Procellariiformes. This is in contrast to the results of the present analysis, which recover Sphenisciformes as the sister taxon to a loon/grebe clade, and Procellariiformes as sister taxon to tropicbirds, though neither relationship is strongly supported. Interestingly, when tropicbirds are excluded from the dataset, MPTs are recovered that include a sister taxon relationship between penguins and procellariforms, which is supported in 63% of boostrap replicates (using the same phylogenetic methodology and search strategies as given in the “Methods” section above). Thus, there is clearly some character support in the present dataset for the procellariform/penguin clade that is well supported in both the Livezey and Zusi [4] and Hackett et al. [29] analyses.

Three other areas of congruence between the datasets center around relationships within Pelecaniformes. All three analyses recover an Anhingidae/Phalacrocoracidae clade ( =  “Phalacrocoracoidea”) with 100% bootstrap support. As noted in the introduction, a close affinity between Anhingidae and Phalacrocoracidae has generally been accepted as uncontroversial, with the exception of several mitochondrial datasets that recovered an unconventional sister taxon relationship between Anhingidae and Sulidae [2], [17]. Kennedy et al. [17] have suggested that long-branch attraction, exacerbated by a short internal branch separating Sulidae, Anhingidae, and Phalacrocoracidae, is likely confounding these datasets. The Livezey and Zusi [4], Hackett et al. [29], and present analyses also all strongly support the monophyly of Suloidea, a clade including Sulidae, Anhingidae, and Phalacrocoracidae. This clade has been recovered in nearly every modern phylogenetic study that has tested pelecaniform and/or waterbird relationships, and there is virtually no controversy regarding its monophyly [22]. Finally, both the current analysis and that of Livezey and Zusi [4] are congruent in recovering strong support for a monophyletic Steganopodes. However, as discussed above, the relative relationships of Pelecanidae and Fregatidae to Suloidea differ between these two analyses, though are not in “strong” conflict with each other, applying the criteria used for this discussion.

Several significant incongruencies between the present analysis, Livezey and Zusi [4], and Hackett et al. [29] are apparent, most of which involve problematic taxa or clades that have been noted previously. Chief among these is the recovery of strong support for a loon/grebe clade in the present analysis and Livezey and Zusi [4], and strong support separating these taxa in Hackett et al. [29]. A loon/grebe clade has been repeatedly tested and rejected by molecular phylogenetic analyses (see review by Mayr [140]), with grebes strongly supported as the sister taxon to flamingos, and this clade recovered well outside of a monophyletic waterbird assemblage. Recently, a growing body of morphological characters have been identified that may also support a close relationship between flamingoes and grebes [29], [140]–[142]. Interestingly, Livezey and Zusi ([4]: p. 48) noted that the majority of characters supporting a loon/grebe clade are from the pelvic girdle and hindlimb. The results of the partitioned analyses from the present dataset also bear out this pattern. The pelvic partition analysis recovers 100% bootstrap support for a sister taxon relationship between loons and grebes, while both the skull and pectoral partition analyses recover these two lineages in polytomies with other taxa (Figures 4 – 6). Coupled with the fact that both loons and grebes are foot-propelled pursuit divers, this pattern raises the possibility that this relationship is the result of morphological convergence in the pelvis and hindlimb of these taxa.

Wiens et al. [143] outlined a list of three explicit criteria for detecting whether adaptive convergence has misled a phylogenetic study. The first, “*strong morphological support for a clade that unites the taxa that share the similar selective environment*” ([143]: p.502), appears to be satisfied for loons and grebes, particularly with reference to characters in the pelvic girdle and hindlimb. Wiens et al.'s ([143]: p. 503) second criterion, “*Evidence that the characters that unite the putatively convergent clade are associated with the shared selective environment*” is slightly more difficult to establish. Hinic-Frlog and Motani [144] reported correlations between foot-propelled diving and the narrowing and elongation of the pelvis, as well as the proximal expansion of the cnemial crest; and indeed, several of the morphological characters supporting a loon/grebe clade in the present analysis are related to these structures (e.g., characters 296, 316, 324, 360, 361). However, the association of these morphological characters with foot-propelled diving remains to be demonstrated across a broader sample of taxa, and with rigorous phylogenetic comparative methods. The third criterion of Wiens et al. ([143]: p. 503), “*Phylogenetic evidence that the species that share the common selective environment are not actually a monophyletic group*, *preferably consisting of strong support for the contradictory clades from two or more unlinked molecular data sets*”, does appear to be satisified. Thus, although the explanation of adaptive convergence related to foot-propelled diving remains a tempting explanation for the loon/grebe signal in morphological datasets, more rigorous functional and comparative phylogenetic analyses are needed to support this claim.

Another point of incongruence present between the present analysis, Livezey and Zusi [4], and Hackett et al. [29], is the position of *Pelecanoides* within Procellariiformes. Livezey and Zusi [4] recover this taxon as the basal-most split within the order, while the present analysis recovers *Pelecanoides* as the sister taxon to a monophyletic Hydrobatidae. Hackett et al. [29] recover *Pelecanoides* as even more highly nested within Procellariiformes, as the sister taxon to *Puffinus*. The postions of *Pelecanoides* in the partitioned analyses are somewhat labile, which may explain some of this conflict (Figures 4–6). As noted above, Procellariiformes are much more diverse than the five taxa that each of the three phylogenetic studies have sampled, so taxon sampling, particularly with respect to the diverse Procellariidae, may be playing a role in this incongruence. However, a more detailed study of relationships within Procellariiformes, albeit based soley on the mitochondrial cytochrome *b* gene, by Nunn and Stanley [145] recovered an arrangement of procellariform families that is generally congruent with that of Hackett et al. [29], with the exception of the position of Oceanitinae.

The relative positions of both *Ciconia* and *Phoenicopterus* are also strongly incongruent between the analyses of Livezey and Zusi [4] and Hackett et al. [29], though not necessarily between either of these datasets and the present analysis, which has difficultly confidently placing either of these taxa (Figures 2 – [Fig pone-0013354-g004] [Fig pone-0013354-g005] 6). The relationships of flamingos within higher-level avian phylogeny as inferred from morphological data have long been contentious, with purported affinities including Anseriformes, Ciconiidae, and Recurvirostridae [30], [142]. Livezey and Zusi [4] recover flamingos and storks as strongly supported sister taxa, well nested within a clade of other ciconiiforms. Flamingos and storks are particularly labile in the present analyses (Figures 2–6). The results of the partitioned analyses (Figures 4–6) suggest that the phylogenetic signal driving their union as sister taxa and derived placement relative to other ciconiiforms may be predominantly coming from characters in the hindlimb (e.g., characters 337, 384, 393, 415, 418), though few characters in the present dataset appear to be unique to these two taxa. As previously noted, a large body of recent molecular evidence supports a sister taxon relationship between grebes and flamingos, with this couplet placed outside the waterbird clade [140]. The position of storks in recent molecular studies of higher-level avian phylogeny has been more uncertain. Hackett et al. [29] recover storks with strong support as the basal-most split of a mixed clade of pelecaniforms and ciconiiforms, though Ericson et al. [27] failed to resolve the position of storks relative to other waterbirds, and the mitogenomic study of Brown et al. [28] recovered storks in an unorthodox position as the sister taxon to steatornithid caprimulgiforms in their optimal topology, though these authors noted that this arrangement was suspect.

Another strong point of conflict between the present analysis and the analyses of Livezey and Zusi [4] and Hackett et al. [29] concerns the relationships of *Scopus*, *Balaeniceps*, and *Pelecanus* within waterbirds. Livezey and Zusi [4] recover *Pelecanus* as the sister taxon to Suloidea, with frigatebirds and tropicbirds as successive sister taxa to this clade in a monophyletic Pelecaniformes. *Balaeniceps* is recovered as the well-supported sister taxon to Pelecaniformes, while *Scopus* is allied with several other ciconiiforms. The results of the present analysis are more similar to those of Mayr [22], [23] and Bourdon et al. [26], in recovering a paraphyletic grade including *Scopus*, *Balaeniceps*, and *Pelecanus*, leading to a *Fregata* + Suloidea clade (Figures 2,3). Though it should be noted that the position of *Pelecanus* and *Fregata* is variable between these studies, and these datasets included few [22], [23], or no [26], other members of a traditional Ciconiiformes. In contrast, a large body of molecular data, including the anlaysis of Hackett et al. [29], supports a monophyletic clade of *Scopus*, *Balaeniceps*, and *Pelecanus*, though the relationships among these three taxa are not robustly resolved (see review by Mayr [140]).

As originally noted by Cottam [146] (though see also Mayr [22], [140]), there are several features suggestive of a close relationship between *Pelecanus* and *Balaeniceps*. An additional character identified in the present study that is unique to *Pelecanus* and *Balaeniceps* is the presenc of a rugose tuberosity distal to the bicipital crest on the proximal humerus (character 205). Both *Pelecanus* and *Balaeniceps* also have sterni with a costal margin that is extremely long relative to other waterbirds (character 127). *Scopus*, *Pelecanus*, *Balaeniceps*, and also *Fregata* appear to be unique in possessing a impression for m. biceps brachii on the coracoid that is situated significantly cranially, well above the facies articularis clavicularis (character 181). Note however, that this likely represents a facies articularis clavicularis that is more caudally placed on the cranial end of the coracoid, rather than a cranial migration of impressio m. biceps brachii. The condition in *Fregata* is difficult to code considering the fusion of the coracoid and furcula. However, the impressio ligamenti acrocoracohumeralis is situated well cranial to the area of fusion between the furucla and coracoid, and a pit-like depression that likely represents impressio m. biceps brachii is located just ventrally and caudomedially to the cranial end of impressio ligamenti acrocoracohumeralis, and is also well cranial to the fused furcula and coracoid. The possibility thus exists that morphological characters supporting a *Scopus*, *Pelecanus*, and *Balaeniceps* have gone previously unnoticed.

A final prominent area of conflict between the present analysis and the analyses of Livezey and Zusi [4] and Hackett et al. [29] regards the relationships of Phaethontidae (tropicbirds). The analysis of Livezey and Zusi [4] recovers tropicbirds in a traditional position, as the well supported, basal-most split within a monophyletic Pelecaniformes. Tropicbirds are also fairly nested within waterbirds in this analysis, being subtended by two more inclusive, well-supported clades. In contrast, the present analysis recovers tropicbirds as the sister taxon to Procellariiformes, similar to recent studies by Bourdon [25] and Bourdon et al. [26]. This position is not particularly well supported in the present analyses (Figures 2–6), though the clade uniting *Balaeniceps* and Steganopodes (and thus excluding tropicbirds), is strongly supported, which conflicts with the results of Livezey and Zusi [4]. In contrast, a growing body of molecular data (e.g., [19], [27]), including the Hackett et al. [29] analysis, strongly supports the placement of tropicbirds well outside of Pelecaniformes, and even outside the waterbird clade, though the close relatives of tropicbirds remain variable in these studies. The recent mitogenomic study of Brown et al. [28] recovered tropicbirds as the sister taxon to penguins, nested within a larger clade primarily composed of waterbirds. These authors' noted the dubious nature of this novel placement however, as was similarly the case with the placement of storks in their analysis [28].

The phylogenetic postion of tropicbirds is thus strongly incongruent and considerably variable among both morphological and molecular studies of higher-level avain phylogeny. Indeed, Cracraft ([3]: p. 834) noted that with regard to the Pelecaniformes, “phaethontids are the most aberrant family of the order”. Given that the divergence of crown Phaethontidae is relatively recent, likely within the past several million years [31], and that stem tropicbirds constrain the split of Phaethontidae from its closest extant relative to more than 55 Myr [26], [53], it is clear that the phylogenetic placement of tropicbirds could be susceptible to long-branch attraction in molecular datasets. Similar circumstances are present for both grebes and flamingos, where long-branch attraction has also been considered as a possible confounding factor [4]. In the present analysis, a variety of characters supporting a close relationship between tropicbirds and Procellariiformes are listed and described above in the section “*Relationships of Prophaethontidae and Phaethontidae*”. In addition to these, there are several characters where states present in either of the stem tropicbird fossil taxa *Prophaethon* and *Lithoptila* are different from those present in extant tropicbirds, and add support for a possible affinity with Procellariiformes (e.g., characters 232, 239, 366), or help to reinforce that the states present in Phaethontidae are not synapomorphic with the same states present in the basal steganopods *Pelecanus* and *Fregata* (e.g., characters 226, 362). Though the sister taxon relationship between tropicbirds and Procellariiformes recovered in the present analysis and by Bourdon [25] and Bourdon et al. [26] should be regarded as tentative, it is intriguing that the mosaic of character states present in stem tropicbirds adds support to this hypothesis, a potential phylogenetic benefit of fossil data that has long been recognized [147]–[150].

With regard to the patterns of higher-level waterbird phylogeny recovered in the present analysis and those of Livezey and Zusi [4] and Hackett et al. [29], there generally is more agreement between the former two morphological datasets than between either of these and the latter molecular dataset. Similarly, more conflict seems to be present between the Hackett et al. [29] phylogeny and either the present phylogeny or that of Livezey and Zusi [4], than between the two morphological phylogenies. Neither of these patterns is perhaps surprising. Potential causes for these conflicts are numerous, and include: **1)** taxon sampling artifacts (with regard to the position of *Pelecanoides*); **2)** limited character sampling (with regard to possible affinities between *Scopus*, *Balaeniceps*, and *Pelecanus*); **3)** adaptive convergence (with regard to support for a loon/grebe clade, but possibly also a stork/flamingo clade); and **4)** long-branch attraction (with regard to the aberrant placements of tropicbirds, the mitochondrial support for an *Anhinga*/Sulidae clade, and possibly also support for a flamingo/grebe clade). Many of these potential causes remain speculative however, and require further evaluation with rigorous comparative phylogenetic methods. Finally, the possibility remains that many higher-level waterbird clades represent temporally deep divergences that are separated by relatively short internodes. This pattern is born out by recent analyses of molecular [27]–[29] and morphological [4], [93] data, which both appear to recover higher-level internodes that are shorter, in terms of inferred character changes, relative to shallower internodes and terminal branches subtending and comprising the tips of the phylogenies. Paleontological evidence supports such a pattern as well, with many modern orders in place and anatomically distinct by the Paleogene, and evidence of pre-Cenozoic records for most neoavian clades scarce and often controversial [26], [151].

Several patterns regarding higher-level relationships inferred from the present morphological dataset are also noteworthy. To begin with, the significant conflict and incongruence regarding the position of several taxa in recent molecular analyses (e.g., [27], [29]) and the Livezey and Zusi [4] morphological analysis, is reduced in the present dataset. These cases involve the phylogenetic positions of: **1)** *Ciconia* and *Phoenicopterus*; **2)** *Scopus*, *Balaeniceps*, and *Pelecanus*; and **3)** Phaethontidae. In none of these three cases are the results of the present analysis congruent with recent molecular analyses (e.g., [27], [29]), but strongly conflicting signals in the morphological dataset appear to be absent. Furthermore, it is evident from the partitioned analyses (Figures 4 – 6) that incongruence may be attributed to characters in a particular anatomical region, rather than wholesale conflicting signal throughout the skeleton. This gives hope that problematic morphological characters capable of misleading phylogenetic analyses may be able to be identified using modern comparative methods, a point stressed previously by McCracken et al. [152]. The results of the present analysis also suggest that the inclusion of stem fossil members of highly modified and deep diverging extant lineages may aid in resolving the phylogenetic position of problematic taxa (e.g., tropicbirds), by preserving deep synapomorphies and revealing superficial convergence. Clearly however, there is need for more morphological and molecular data, more rigorous evaluation of this data, and more comprehensive phylogenetic analyses, a point that all avian systematists appear to be in agreement on [4], [27]–[29], [93], [150], [151]. Despite the obvious need for well-supported morphological phylogenies that can accurately place fossil taxa in order to assess the tempo of avian diversification, the continued collection and intergration of morphological and molecular datasets will ultimately provide reciprocal illumination of higher-level avian phylogeny and evolution.

### Conclusions

The monophyly and phylogenetic relationships of the avian order Pelecaniformes were assessed through the analysis of a morphological phylogenetic dataset of waterbirds encompassing 59 taxa and 464 characters. Parsimony analyses do not recover a monophyletic Pelecaniformes, recovering tropicbirds as distantly related to the remaining members of the order, which are supported as a monophyletic Steganopodes (pelicans, frigatebirds, sulids, darters, cormorants). Relationships of extant pelecaniforms inferred from morphology are more congruent with molecular phylogenies than previously assumed, though notable conflicts (e.g., the positions of *Pelecanus* and *Phaethon*) remain. ILD tests suggest that some major anatomical partitions of the dataset may possess different phylogenetic signals, though partitioned phylogenetic analyses reveal that these discrepancies are localized outside of Steganopodes, and can primarily be attributed to a few problematic taxa (e.g., flamingos, tropicbirds, hammerkop), or poorly supported nodes. The Plotopteridae, an extinct family of wing-propelled divers, are recovered as the monophyletic sister taxon to a cormorant–darter clade, suggesting numerous convergent adaptations in the pectoral limbs of plotopterids and penguins. However, support for this topology is relatively weak, and the higher-level relationships of Plotopteridae recovered here remain tentative.

The relationships of several fossil pelecaniforms representing key calibration points for studies of higher-level avian diversification are well resolved in the present study. These include *Limnofregata* (sister taxon to Fregatidae), *Prophaethon* and *Lithoptila* (successive sister taxa to Phaethontidae), and ?*Borvocarbo stoeffelensis* (sister taxon to Phalacrocoracidae). The sister taxon relationships of these fossil taxa are robust to alternate phylogenetic hypotheses, and do not change when ‘backbone’ phylogenetic constraints based on recent morphological and molecular studies of higher-level avian phylogeny are imposed. However, the successive outgroup relationships of several of these “stem fossil + crown family” clades (e.g., *Limnofregata*/Fregatidae; *Lithoptila*/Phaethontidae) remain highly variable and poorly supported across recent studies of avian phylogeny. Thus, the impact these fossil calibrations have on future studies of higher-level avian temporal diversification will depend heavily on the extant sister taxon relationships of Phaethontidae and Fregatidae, as well as the increased resolution and support of deep nodes in avian phylogeny.

## Supporting Information

Appendix S1 List of specimens examined.
(0.02 MB DOC)

Appendix S2 Morphological character list.
(0.16 MB DOC)

Appendix S3 Morphological data matrix.
(0.12 MB DOC)
