# Supplementary material for: Phylogenetic Analysis of Pelecaniformes (Aves) Based on Osteological Data: Implications for Waterbird Phylogeny and Fossil Calibration Studies
Source: PLoS One. 2010 Oct 14;5(10):e13354. doi: 10.1371/journal.pone.0013354 (PMC2954798; doi:10.1371/journal.pone.0013354)
Supplement: Appendix S1 — List of specimens examined. (0.02 MB DOC) [file pone.0013354.s001.doc]

# Appendix S1: List of specimens examined

## Institutional abbreviations

**FMNH**, The Field Museum of Natural History, Chicago, Illinois; **KMNH**, Kitakyushu Museum and Institute of Natural History, Kitakyushu, Japan; **MACN**, Museo Argentino de Ciencias Naturales, Buenos Aires, Argentina; **USNM**, National Museum of Natural History, Smithsonian Institution, Washington, D.C.; **UWGM**, University of Wyoming Geological Museum, Laramie, Wyoming; **WSGS**, Wyoming State Geological Survey, Laramie, Wyoming.

*Fossil specimens*

?*Borvocarbo stoeffelensis* [51,52]; *Copepteryx hexeris* USNM 486682 (holotype; cast of KMNH VP 200,006), USNM 243773 (paratype; cast of KMNH VP 200,001), USNM 486684 (paratype; cast of KMNH VP 200,002), USNM 243774 (paratype, cast of NSMT VP 15035), [4]; *Cocopteryx titan* USNM 486685 (holotype; cast of KMNH VP 200,004), [4]; *Limnofregata azygosternon* USNM 22753 (holotype), UWGM 6919 (paratype), FMNH PA 720, 723, 731, 755, [32,56]; *Limnofregata hasegawai* FMNH PA 719 (paratype), WSGS U1-2001, [32,56]; *Lithoptila abdounensis* [25,50]; *Phocavis maritimus* [57]; *Plotopterum joaquinensis* USNM 8927 (cast of LACM 8927), [40]; *Prophaethon shrubsolei* [58,59]; *Tonsala hildegardae* USNM 256518, [47]; *Tonsala*? sp. USNM 243775 (cast of KMNH VP 200,003).

*Extant specimens*

*Anhinga anhinga* FMNH 339387, 375499, 105995; *Anhinga rufa* FMNH 368755; *Ardea herodias* FMNH 441541; *Balaeniceps rex* FMNH 104903, 104644; *Ciconia abdimii* FMNH 347518, 368771, 339233; *Cochlearius cochlearius* FMNH 104961; *Phoebastria nigripes* FMNH 339601; *Eudocimus albus* FMNH 375819; *Eudromia elegans* FMNH 342227; *Eudyptula minor* FMNH 106505, 339521; *Fregata ariel* USNM 498024, 498347; *Fregata magnificens* FMNH 339418, 360077; *Fregata minor* FMNH 339421, 104235, 104717; *Gallus gallus* FMNH 104665; *Gavia immer* FMNH 342276, 458777, 444971, 461817; *Morus bassanus* FMNH 364580; *Morus capensis* USNM 558368; *Morus serrator* FMNH 339366; *Oceanites oceanicus* FMNH 339531; *Oceanodroma castro* FMNH 339537; *Papasula abbotti* USNM 560862, 560863; *Pelecanoides urinatrix* FMNH 339530, 289071; *Pelecanus erythrorhynchos* FMNH 445082; *Phaethon aethereus* FMNH 348136; *Phaethon lepturus* FMNH 339442; *Phaethon rubricauda* FMNH 313967; *Phalacrocorax africanus* FMNH 368742, 384664; *Phalacrocorax albiventer* FMNH 348121; *Phalacrocorax aristotelis* FMNH 348123; *Phalacrocorax atriceps* USNM 489485, 490792; *Phalacrocorax auritus* FMNH 348372; *Phalacrocorax bougainvillii* FMNH 339417; *Phalacrocorax brasilianus* FMNH 375472, 105997; *Phalacrocorax carbo* FMNH 339390, 368739; *Phalacrocorax gaimardi* MACN 54415; *Phalacrocorax magellanicus* MACN 54414; *Phalacrocorax melanoleucos* USNM 561522; *Phalacrocorax pelagicus* FMNH 339415; *Phalacrocorax penicillatus* FMNH 339409; *Phalacrocorax pygmaeus* USNM 431602; *Phoenicopterus chilensis* FMNH 398896, 454879; *Platalea ajaja* FMNH 360127; *Podiceps auritus* FMNH 363755; *Puffinus gravis* FMNH 364582; *Pygoscelis antarctica* FMNH 390994; *Scopus umbretta* FMNH 313701; *Sula dactylatra* FMNH 339369, 104294; *Sula leucogaster* FMNH 339376, 346052; *Sula nebouxii* FMNH 339367; *Sula sula* FMNH 339372; *Sula variegata* USNM 547923.
