# Supplementary material for: Phylogenetic Analysis of Pelecaniformes (Aves) Based on Osteological Data: Implications for Waterbird Phylogeny and Fossil Calibration Studies
Source: PLoS One. 2010 Oct 14;5(10):e13354. doi: 10.1371/journal.pone.0013354 (PMC2954798; doi:10.1371/journal.pone.0013354)
Supplement: Appendix S2 — Morphological character list. (0.16 MB DOC) [file pone.0013354.s002.doc]

# Appendix S2: Morphological character list

A total of 464 osteological characters were scored for each taxon (Appendix S3). Characters can be divided into coarsely defined anatomical regions as follows: cranial skeleton, 95; axial skeleton, 11; pectoral skeleton, 188; pelvic skeleton, 169; miscellaneous, 1. 88 (19%) characters are new or have been formulated for phylogenetic analysis for the first time. The remaining characters, or some variation thereof, have been utilized previously in phylogenetic analyses. Characters were assembled from a variety of studies, with the primary sources being Cracraft (1985), Siegel-Causey (1988), Warheit (1990), Mayr (2005a), Bourdon et al. (2005, 2006), and Livezey and Zusi (2007a). The primary sources for the characters in the present dataset are listed after the character description. Unless otherwise specified, the number or descriptor following the author and year of each citation represents the character number from that dataset, and not a page number. Note that the extensive documentation present in Livezey and Zusi (2007a) can also been consulted regarding previous usage of many characters.

1. Premaxilla, curvature of distal end of premaxilla: not strongly curved relative to the rest of the rostrum (0); slightly hooked ventral end (1); strongly ventrally curving hook with tip at nearly a right angle to long axis of beak (2). Mayr (2005a:1); see also Livezey and Zusi (2007a:265).

2. Premaxilla, premaxilla pneumatized by rostral diverticulum of sinus antorbitalis: absent (0); present (1). Bourdon et al. (2005:8). See also Livezey and Zusi (2007a:372); Witmer (1990:337).

3. Premaxilla, dorsal surface of nasal process, just anterior to nasofrontal hinge (when present): flat or slightly convex in lateral view, with roughened surface (0); slightly concave in lateral aspect, and smooth (1). Warheit (1990:Skl9).

4. Processus maxillaries of palatinum and processus maxillopalatini of maxillare largely fused: absent (0); present (1). Mayr (2005a:6); see also Livezey and Zusi (2007a:289).

5. Maxilla, cross-sectional shape at posterior end, near level of end of nasal grooves (when present): ventral margin of maxilla broader mediolaterally than the dorsal margin, resulting in a trapezoidal cross-section (0); ventral and dorsal margins equal or nearly so in mediolateral breadth, resulting in a square or rectangular cross-section (1). Warheit (1990:Skl12).

6. Maxilla, Marked furrow on lateral side of maxilla (nasal groove) extending distally from osseous narial opening: absent (0); present (1). Cracraft (1985:4); see also Livezey and Zusi (2007a:272).

7. Maxilla, nasal groove (when present), degree of excavation at posterior extent: shallow with relatively indistinct rims (0); deeply excavated, with well-developed rims (1). Siegel-Causey (1988:9).

8. Nares, osseous narial openings: large (0); reduced (1); extremely reduced to a minute foramen or absent (2). Mayr (2005a:3); see also Livezey and Zusi (2007a:331).

9. Nares, bony depression accommodating regio olfactoria of cavum nasi greatly expanded ventrally and caudally, directly communicating with fossa bulbi olfactorii: absent (0); present (1). Bourdon et al. (2005:38). See also Livezey and Zusi (2007a:42).

10. Nasal, anterior portion of osseous narial openings very close to dorsal midline, with a mediolaterally flattened internarial bar: absent (0); present (1). Bourdon et al. (2005:3).

11. Nasal, nasofrontal hinge: absent (0); present (1). Livezey and Zusi (2007a:604). See also Mayr (2005a:2).

12. Lacrimal, large circular perforation in lacrimal body: absent (0); present (1). Livezey and Zusi (2007a:196), Ksepka et al. (2006:83).

13. Lacrimal, pneumaticity associated with lacrimal: absent (0); present (1). Livezey and Zusi (2007a:197).

14. Lacrimal, shape of anteroventral surface: smooth and unornamented (0); triangular fossa excavated onto surface, with distinct medial and ventral rims (1). Siegel-Causey (1988:20).

15. Ectethmoid, vestigial or completely absent: no (0); yes (1). Mayr (2005a:7); see also Livezey and Zusi (2007a:189).

16. Mesethmoid: unfenestrated (0); large midline fenestra present on anterodorsal portion of mesethmoid (1). Siegel-Causey (1988:16).

17. Palatine, pars choanalis very deep dorsoventrally: no (0); yes (1). Mayr (2005a:9). See also Livezey and Zusi (2007a:436).

18. Palatine, pronounced lateromedial constriction of lateral and medial surfaces of lamina choanalis dorsalis of pars choanalis at rostral end: absent (0); present (1). Livezey and Zusi (2007a:437).

19. Palatine, ventral process of pars choanalis present only at pterygoid articulation: absent (0); present (1). Cracraft (1985:1); Bourdon et al. (2005:7); Warheit (1990:Skl10).

20. Palatine, width of palatines at pterygoid articulation relative to width anterior to articulation: narrow, pterygoid articulation forms distinct ‘neck’ less than half of width of joined palatines (0); broad, pterygoid articulation is not as wide as joined palatines anteriorly, but still more than half of width (1); identical, there is no distinct transition or narrowing of the conjoined palatines at the pterygoid articulation (2). Siegel-Causey (1988:32).

21. Palatine, palatines fused along midline: no (0); yes (1). Mayr (2005a:10). See also Livezey and Zusi (2007a:440).

22. Palatine, dorsal surface of palatine a nearly flat, horizontal plate: no (0); yes (1). Mayr (2005a:11).

23. Vomer: present (0); absent (1). Cracraft (1985:9); see also Mayr (2005a:12), and Livezey and Zusi (2007a:461).

24. Vomer, shape of fused vomers: roughly cylindrical to blade-like (0); dorsoventrally flattened and laminate (1). Modified from Livezey and Zusi (2007a:462); see also Mayr and Clarke (2003:20).

25. Jugal, fenestra ventrolateralis (sensu Livezey and Zusi, 2007a:290) on ventral face of maxillary rostrum: absent, not entirely enclosed caudally (0); present, closed caudally by osseous strut between jugal and maxilla (1). Livezey and Zusi (2007a:290).

26. Jugal, degree of ventral curvature: absent or weak (0); strongly bowed ventrally (1). Modified from Livezey and Zusi (2007a:298).

27. Jugal, pneumatic foramina associated with rostral end of jugal: absent (0); present (1). Livezey and Zusi (2007a:304).

28. Suprajugal: absent (0); present (1). Livezey and Zusi (2007a:720).

29. Quadrate, orientation of the squamosal and otic condyles relative to the long axis of the skull: obliquely oriented, angle between 20-75º (0); nearly perpendicular, angle between 75-90º (1); nearly parallel, angle less than 20º (2). Livezey and Zusi (2007a:148).

30. Quadrate, shape of otic head in dorsal aspect: round or bulbous (0); compressed anteroposteriorly and distinctly elongate mediolaterally (1). New Character.

31. Quadrate, pneumatic foramen at ventromedial base of orbital process: absent (0); present (1). Livezey and Zusi (2007a:508).

32. Quadrate, pneumatic foramen at caudomedial edge of quadrate shaft, just ventral to otic process: absent (0); present (1). Livezey and Zusi (2007a:509).

33. Quadrate, strong lateral ridge bounding sulcus on caudal shaft of quadrate, trending obliquely from orbital process to pterygoid articulation: absent (0); present (1). New Character.

34. Quadrate, pneumatic foramen/foramina on caudal surface of dorsal end of quadrate, between proximal condyles: absent (0); present (1). Mayr and Clarke (2003:36); Livezey and Zusi (2007a:554).

35. Quadrate, orbital process reduced in dorsoventral thickness, tapering to a point distally: no (0); yes (1). Mayr (2005a:20). See also Livezey and Zusi (2007a:533, 539); Bourdon et al. (2005:40).

36. Quadrate, relative elongation of orbital process (exceeding otic process in length) and rostral expansion into spatulate tip (latter at least as broad as any other part of the orbital process): absent (0); present, rostral tip broadly rounded (1); present, rostral tip spatulate with bifid appearance (2). Livezey and Zusi (2007a:535).

37. Quadrate, robust suboval tuberosity projecting caudomedially from caudolateral edge of quadrate shaft: absent (0); present (1). New Character.

38. Quadrate, intercondylar sulcus of mandibular process a deep, parabolic (“U-shaped”) channel with sides (craniocaudal perspective) subdiagonal (typically parallel) and directly opposite each other and dorsally foveate (ventral perspective): absent (0): present (1). Livezey and Zusi (2007a:529).

39. Quadrate, pneumatic foramina associated with intercondylar sulcus of mandibular process of quadrate: absent (0); present (1). Livezey and Zusi (2007a:528).

40. Frontal, suclus for the nasal gland marked and situated on dorsal surface of supraorbital margin of frontal: no (0); yes (1). Mayr (2005a:18). See also Livezey and Zusi (2007a:20).

41. Frontal, suture with lacrimal: facing laterally (0); facing ventrally and not obliterated in adults (1); facing ventrally and obliterated in adults (2). Bourdon et al. (2005:9). See also Livezey and Zusi (2007a:564).

42. Frontal, convexity of dorsal surface of skull between lacrimals: relatively flat to concave (0); strongly convex (1). Bourdon et al. (2005:10). See also Livezey and Zusi (2007a:14).

43. Frontal, relative width of frontal in interorbital region: widened mediolaterally in area of contact with lacrimal (0); constant, not enlarged mediolaterally in area of contact with lacrimal (1). Bourdon et al. (2005:11).

44. Frontal, fossa for nasal gland situated within roof of orbit, in rostromedial position: absent (0); present (1). Bourdon et al. (2005:39).

45. Frontal, extent of excavation on ventral side of frontal for nasal gland: small, confined to the anterior portion of the frontal (0); large and expansive, extending to the posterior margin of the orbit (1). Siegel-Causey (1988:12, 13).

46. Frontal, prominent angulus postocularis: absent (0); present (1). Livezey and Zusi, 2007a:175).

47. Processus postorbitalis, length and orientation: long and ventrally oriented (0); short and ventrolaterally oriented (1). Warheit (1990:Skl 6).

48. Processus postorbitalis, shape of distal end: single and unforked (0); single but with a smaller secondary branch (1); distinctly forked with two branches of equal length (2). Warheit (1990:Skl7).

49. Processus postorbitalis, ventral surface: smooth or slightly concave, with no depression of pneumatopores (0); large fossa present (1). Warheit (1990:Skl8).

50. Squamosal, relative length of rostral border of squamosal that joins zygomatic process and caudal wall of orbit: not elongated (0); elongate and thin, with constant thickness throughout (1). Bourdon et al. (2005:14).

51. Squamosal, zygomatic process extending laterally as a dorsally convex horizontal platform: absent (0); present (1). Cracraft (1985:48); Bourdon et al. (2005:16).

52. Squamosal, crista postzygomaticas, shape of ventral margin in lateral aspect: linear, or slightly concave ventrally (0); strongly concave ventrally, creating upside-down U-shape in lateral aspect and broadly exposing the otic head of the quadrate (1). New Character.

53. Squamosal, ventrally prominent triangular flange extending from crista postzygomatica: absent (0); present (1). New character, though see also Livezey and Zusi (2007a:143).

54. Squamosal/Prootic, pila otica elongated, strongly protruding caudoventrolaterally, so that cotyla quadratica otici faces laterally: absent (0); present (1). Bourdon et al. (2005:30).

55. Laterosphenoid/prootic, fonticulus laterospheno-proötica: absent (0); present (1). Livezey and Zusi (2007a:101).

56. Basioccipital, glossopharyngeal nerve (IX): enclosed in a foramen or notch in the metotic process: (0); lacks a distinct foramen (1). Bourdon et al. (2005:23); Livezey and Zusi (2007a:62).

57. Basioccipital, metotic process, foramen or notch for passage of arteria ophthalmica externa near lateral edge: present (0); absent (1). Bourdon et al. (2005:24).

58. Basioccipital, area of lamina parasphenoidalis ventral to occipital condyle and medial to basal tubera: apneumatic (0); two large, symmetrical pneumatopores present (1). Warheit (1990:Skl1).

59. Basioccipital, tuberculi basilares high and compressed anteroposteriorly, with lamina parasphenoidalis strongly sloping towards their tip: absent (0); present (1). Bourdon et al. (2005:26).

60. Supraoccipital, vena occipitalis externa: piercing occipital plate to exit brain cavity (0); exiting brain cavity through foramen magnum (1). Bourdon et al. (2005:78); Livezey and Zusi (2007a:79).

61. Supraoccipital, fonticulus occipitalis: absent (0); present (1). Livezey and Zusi (2007a:78).

62. Basipterygoid processes, relative development: well-developed, contribute to articulation with ossa pterygoidea (0); vestigial or absent (1). Mayr (2005a:13); Livezey and Zusi (2007a:108).

63. Parasphenoid, rostrum parasphenoidale extremely thin mediolaterally, particularly at its caudel end: no (0); yes (1). Bourdon et al. (2005:37); Livezey and Zusi (2007a:106).

64. Parasphenoid, lamina parasphenoidalis (‘basitemporal plate’) essentially flat and rostrolaterally bordered by marked osseous walls, tubercula basilaria well-developed: no (0); yes (1). Mayr (2005a:15). See also Livezey and Zusi (2007a:117, 123), Bourdon et al. (2005:25), and Warheit (1990:SKL3).

65. Parasphenoid, orientation of lamina parasphenoidalis at its caudal end: planar, flat, facing ventrally (0); bent, convex, facing ventrocaudally (1). Warheit (1990:Skl2).

66. Paroccipital processes, distal tips protrude strongly caudally: no (0); yes (1). Cracraft (1985); Mayr (2005a:14), Bourdon et al. (2005:21); and Livezey and Zusi (2007a:132).

67. Dorsal tympanic recess, rostroventral position relative to quadrate cotyles: caudal to intermediate between cotyles (0); main portion of recess situated rostral/rostromedial of cotyles (1). Mayr (2005a:16). See also Bourdon et al. (2005:31) and Livezey and Zusi (2007a:223).

68. Dorsal tympanic recess, greatly enlarged, much longer than large, extending rostral to and between cotylae quadratica in a figure-8 shape: absent (0); present (1). Bourdon et al. (2005:32).

69. Dorsal tympanic recess, subdivision into two primary openings, a larger opening rostromedial to the quadrate cotyles and outside of the quadrate articular facies, and a smaller opening caudolateral to it, but within the quadrate articular facies: absent (0); present (1). New Character.

70. Dorsal tympanic recess, craniocaudally oriented bony strut separating two subdivided openings of dorsal tympanic recess (when present, taxa lacking this feature coded as “–“ inapplicable): present (0); absent (1). New Character.

71. Rostral tympanic recess, relative development of posterolateral flange of ala parasphenoidale: large, posterolaterally expanded flange (0); extremely reduced or absent (1). Cracraft (1985:14). See also Bourdon et al. (2005:28), and Livezey and Zusi (2007a:229).

72. Foramen nervi maxillomandibularis location relative to entrance of recessus tympanicus rostralis: rostral (0); caudal (1). Bourdon et al. (2005:29); Livezey and Zusi (2007a:46).

73. Nervi optici, oculomotorii, trochlearis, and ophthamilci: not confluent or only partially so (0); entering orbit through common opening (1). Bourdon et al. (2005:36). See also Livezey and Zusi (2007a:248) for a similar character.

74. Foramen nervorum olfactorii: present, n. olfactorii passing at least in part lateral to paries medialis orbitae (0); absent, n. olfactorii passing internally within the interorbital region of the skull, medial to paries medialis orbitae and paries dorsalis orbitae, and ventral to os frontale (1). Livezey and Zusi (2007a:41).

75. Cranium, strongly dorsoventrally compressed, producing nearly flat dorsal surface to cranium that is much wider mediolaterally than deep dorsoventrally: absent (0); present (1). Siegel-Causey (1988:10). See also Livezey and Zusi (2007a:6).

76. Cranium, fossae temporales marked and extending to midline of cranium: no (0); yes (1). Mayr (2005a:17). See also Cracraft (1985:47); Bourdon et al. (2005:13); Livezey and Zusi (2007a:16); Warheit (1990:Skl11).

77. Cranium, prominentia cerebellaris, shape of ventral surface: relatively non-distinct rim (0); slightly anteroposteriorly expanded with flat to slightly concave ventral surface (1). New Character.

78. Cranium, prominentia cerebellaris, marked, abrupt caudal extension–extending caudad to crista (linea) nuchalis transversa–and conformation as smooth, rounded, subglobular bulla lacking distinguishable linea aut carina medialis, status: absent (0); present (1). Livezey and Zusi (2007a:31). See also Chu (1998:3).

79. Cranium, area of m. protractor pterygoideus origin (or homologous area) lateral to foramen opticum: unmarked or weakly raised ridge (0); strong cranially expanded flange (1). New Character.

80. Cranium, temporal region: lacking well-developed zygomatic process (0); with well-developed zygomatic process, large fossa temporalis mainly occupied by m. adductor madibulae externus pars coronoidea (1); as in state 1, and zygomatic process continuous with high crest (crista temporalis ventralis) separating m. adductor mandibulae externus pars coronoidea rostrally and m. adductor mandibulae externus pars articularis caudally (2). Bourdon (2005:66). See also Livezey and Zusi (2007a:147).

81. Cranium, Crista temporalis ventralis (when present), shape in dorsal aspect: slightly concave anteriorly, creating a U-shape in dorsal view (0); relatively straight, and flat anteriorly, resulting in a V-shape in dorsal view (1). New Character.

82. Cranium, caudally prominent, rounded, ventrally concave lamina medialis nuchalis (sensu Livezey and Zusi, 2007a:34) on midline of crista nuchalis transversa: absent (0); present (1). Livezey and Zusi (2007a:34).

83. Cranium, elongate, concave, triangular lamina bordered by cristae (i) nuchalis transversae, (ii) nuchalis lateralis, (iii) otica dorsalis, and (iv) post zygomatica, status: absent (0); present (1). Livezey and Zusi (2007a:35).

84. Cranium, broad, concave, triangular lamina bordered by cristae (i) nuchalis lateralis, (ii) otica dorsalis, and (iii) m. depressor mandibulae: absent (0) present (1). Livezey and Zusi (2007a:36). See character 83 above for a similar, but topologically distinct lamina.

85. Cranium, definition of dorsal boundary (at junction of crista nuchalis transvera and crista musculi depressor mandibulae, when present) for m. depressor mandibulae: fossa faces primarily laterally, not well delineated at dorsal margin (0); fossa faces laterally and slightly dorsally, dorsal margin well demarcated by two sharp crests forming the apex of a triangle (1). Siegel-Causey (1988:21).

86. Cranium, second postorbital (temporal) process (sensu Siegel-Causey, 1988), status: absent or indistinct (0); present, prominent and well developed as a triangular eminence (1). Siegel-Causey (1988:6).

87. Number of scleral ossicles: 14 or more (0); 12-13 (1); 10 (2). Warheit et al. (1989). See also Mayr (2005a:21), and Livezey and Zusi (2007a:716).

88. Styliform process at caudal end of cranium: absent (0); present (1). Mayr (2005a:22).

89. Mandible, dorsal surface of mandibular rami mediolaterally wide with relatively planar dorsal surface, marked by a shallow longitudinal sulcus and low medial and lateral ridges: no (0); yes (1) (Mayr, 2002).

90. Mandible, relative rostrocaudal length of pars symphysialis: long (0); short (1). Mayr (2002); Livezey and Zusi (2007a:676).

91. Mandible, surangular, area at posteromedial end of attachment of M. adductus mandibulae externus profundus: indistinct or lacking tuberosity (0); presence of a single robust, knob-like tuberosity (1); presence of a large, bipartite flange (2). Siegel-Causey (1988:41). See also Owre (1967:Fig. 54D).

92. Mandible, articular, relative size of the dorsally facing pneumatic foramen located posteromedial to the quadrate cotyles: large (0); very small and pinhole sized (1). New Character, though see Livezey and Zusi (2007a:627).

93. Mandible, angular, dorsal extension of lateral edge of retroarticular process: relatively unmarked from surrounding border (0); distinct dorsally elongated tab-like process (1). Livezey and Zusi (2007a:620).

94. Mandible, relative size of fossa aditus: large (0); small, fossa typically no larger than associated neurovascular canal (1). Siegel-Causey (1988:46); see also Livezey and Zusi (2007a:689).

95. Mandible, orientation of tuberculum intercotylare: relatively low to dorsally prominent mound-shaped process (0); distinct caudodorsal curl, forming a weak “lip” overlapping the cranial face of the medial quadrate condyle (1). Livezey and Zusi (2007a:710).

96. Atlas, morphology of dorsal rim of atlantal body: dorsal rim incomplete, with a broad gap between paired transverse ligament tuberosities (0); dorsal rim complete, resulting in peforate atlantal body (1). Livezey and Zusi (2007a:771).

97. Axis, ansa costotransversaria: present (0); absent (1). Livezey and Zusi (2007a:774).

98. Axis, pneumatic recesses in lateral body of axis: present (0); absent (1). Livezey and Zusi (2007a:782).

99. Axis, broad contralateral communication of lateral pneumatic recesses (when present): absent (0); present (1). New Character.

100. Axis, development of prezygapophyses: present and distinct (0); extremely rudimentary or obsolote (1). Livezey and Zusi (2007a:796).

101. Number of cervical vertebrae: 13 or 14 (0); 15 or 16 (1); 17 or more (2). Modified from Livezey and Zusi (2007a:798).

102. Osseous bridge from processus transversus to processus articularis caudalis on third cervical vertebra: absent (0); present (1). Mayr and Clarke (2003:Fig 6D). See also Mayr (2005a:24); Livezey and Zusi (2007a:806).

103. Cervical vertebrae 8-11 with processus carotici ankylozed along the midline, forming an osseous canal: no (0); yes (1). Mayr (2005a:25).

104. Mid-Posterior cervicals, bifurcation of neural spine: absent (0); present (1). Livezey and Zusi (2007a:831).

105. Caudalmost thoracic vertebrae opisthocoelous: no (0); yes (1). Mayr (2005a:26).

106. Pre-sacral vertebrae, pneumatic recesses on lateral surface of vertebral body: present (0); absent (1). Modified from Livezey and Zusi (2007a:843).

107. Limb bones heavily pachyostotic: absent (0); present (1). Mayr (2005a:50); Livezey and Zusi (2007a:5).

108. Sternum, shape and relative craniocaudal length to mediolateral width of dorsal surface of sternal body: rectangular, sternal body longer than wide (0); square-shaped, sternal body wider than long (1); elongate rectangular, sternal body more than twice as long than wide (2). Modified from Livezey and Zusi (2007a: 1099, 1100, 1101).

109. Sternum, postion of caudal end of sternal carinae relative to caudal end of sternal body: carinae extends caudally to, or near to, the caudal end of the sternum (0), caudal end to carinae terminates distinctly short of the caudal end of the sternum (1). Livezey and Zusi (2007a:1103, 1107, 1205).

110. Dorsal surface of sternum: relatively smooth and unmarked (0); bearing numerous pneumatic foramina along midline and lateral margins (1). Mayr (2005a:34); Livezey and Zusi (2007a:1108, 1110); Mayr and Clarke (2003:72).

111. Sternum, transverse low ridge-like striations associated with pneumatic openings on midline of dorsal surface of partes hepatica portion of sternum: absent (0); present (1). Livezey and Zusi (2007a:1113).

112. Sternum, degree of pneumaticity of incisurae intercostales: absent (0); present, few number of small foramina (1); present, heavily pneumatized with numerous foramina (2). Modified from Livezey and Zusi (2007a:1115).

113. Sternum, relative concavity of anteriormost pilae costalis: laterally concave in dorsal or ventral aspect (0); straight, with the anteriormost pila costalis situated in nearly the same plane as the preceeding pilae costalis (1). Modified from Livezey and Zusi (2007a:1117).

114. Number of costal facets on sternum: four (0); five (1); six (2); seven (3). Livezey and Zusi (2007a:1119).

115. Sternum, pronounced, ventrally deflected facet on lateral portion of labrum externum sternae: absent (0); present (1). New Character.

116. Sternum, angle of lateral portion of ventral lip (labrum externum sternae) of coracoid sulcus to midline of sternum: angle greater than 15 degrees, less than 60 degrees (0); angle extremely low, ventral lip and midline nearly parallel to each other (1); angle extremely high, approaching 90 degrees (2). Modified from Lee et al. (1997), Livezey and Zusi (2007a:1128). See also Warheit (1990:STN3).

117. Sternum, pneumaticity of coracoid sulcus and dorsal lip (labrum internum sternae) of the coracoid sulcus: absent (0); present (1). Livezey and Zusi (2007a:1137).

118. Sternum, length of craniolateral process: relatively short and block-like to rectangular (0); extremely elongate, sub-triangular to acuminate (1). Modified from Livezey and Zusi (2007a:1141).

119. Sternum, angle of long axis of craniolateral process relative to midline of sternum: perpendicular, ~90 degrees (0); diagonal, ~45 degrees (1), parallel, ~ 0 degrees (2). Livezey and Zusi (2007a:1142).

120. Sternum, sulcus sellaris medialis (sensu Livezey and Zusi, 2007a:1146) on cranial margin of ventral face of sternum, between coracoid articulations: absent (0); present (1). Livezey and Zusi (2007a:1146).

121. Sternum, relative distribution and position of costal processes: loosely spaced, on lateral margin of sternum and sometimes extending onto base of craniolateral process of sternum (0); tightly packed, majority of costal processes on craniolateral process of sternum (1). Modified from Livezey and Zusi (2007a:1147).

122. Sternum, spina externa rostri: absent (0); present (1). Modified from Livezey and Zusi (2007a:1157). See also Warheit (1990:STN1), Siegel-Causey 1988:96; = “ventral manubrial spine”).

123. Sternum, mediolateral development of craniodorsal tip of spina externa rostri: thin crest to weakly expanded (0); broadly mediolaterally expanded, robust, upside-down triangle in cranial aspect (1). New Character.

124. Sternum, pneumatic openings ventral to spina externa rostri (or corresponding location in taxa lacking spina externa rostri): absent (0); present (1). New Character. See also Livezey and Zusi (2007a:1214, 1215).

125. Sternum, fenestra infraspinousa carinae enclosed by cranial lamina of bone connecting spina externa rostri (when present) to crista medialis carinae: absent (0); present (1). Modified from Livezey and Zusi (2007a:1164, 1215).

126. Sternum, relationship of medial ends of coracoid sulci to each other: separate or contact slightly on the midline (0); overlap medially, such that the medialmost end of the left coracoid sulcus lies dorsal to the medialmost end of the right coracoid sulcus in anterior aspect (1). New Character. See also Warheit (1990:STN4), Livezey and Zusi (2007a:1331).

127. Sternum, length of costal margin relative to length of sternum along dorsal midline: less than 1/3 (0); between 1/3 and 3/4 (1); greater than 3/4 (2). Modified from Livezey and Zusi (2007a:1165).

128. Sternum, laterally flaring ala of sternal lateral margin directly posterior to posterior end of costal margin: absent (0); present (1). Modified from Livezey and Zusi (2007a:1166).

129. Sternum, prominent jugum subcostalis and sulcus medioventralis costorum extending craniocaudally along majority of lateral edge of sternum: absent (0); present (1). Livezey and Zusi (2007a:1173).

130. Sternum, incisura caudolateralis on caudal margin of sternum: present (1); absent (0). Livezey and Zusi (2007a:1182).

131. Sternum, trabecula intermedia, between trabecula caudolateralis and trabecula medialis: absent (0); present (1). Livezey and Zusi (2007a:1186).

132. Trabecula mediana on caudal margin of sternum very short, reaching much less far distally than trabeculae laterales: no (0); yes (1). Mayr (2005a:35); Livezey and Zusi (2007a:1192).

133. Sternum, relative convexity of ventral carinal margin in lateral aspect: moderately convex (0); nearly straight (1); extremely convex, approaching semicircular profile (2). Modified from Livezey and Zusi (2007a:1195). See also Warheit (1990:STN2).

134. Sternum, apex carinae of sternum pointed and projecting far rostrally to coracoid sulci: no (0); yes (1). Mayr (2005a:33); Livezey and Zusi (2007a:1198).

135. Sternum, area of muscle attachment for m. supracoracoideus deliminated by lineae intermuscularis ventromedialis, and lineae intermuscularis dorsolateralis: large, ventral intermuscular line close to ventral margin of sternal keel, and doroslateral intermuscular line extends to near the caudal end of sternal keel (0); truncated caudally, subtriangular depression, doroslateral intermuscular line ends well anterior to caudal end of sternal keel (1). Livezey and Zusi (2007a:1204).

136. Sternum, mediolateral width of ventral margin of sternal keel relative to width just dorsal to ventral margin: approximately equal (0); expanded and distinctly thicker (1). Livezey and Zusi (2007a:1216).

137. Sternum, postion of articular facies for furcula relative to apex carina: distal, approximately at apex carina (0); proximal, furcula articulates caudal to apex carina (1). Livezey and Zusi (2007a:1319).

138. Claviculae, relative development of sternal articular facet: articular facet for sternum not well-developed (0); robust, well-developed articular facet for the apex carinae of the carina sterni (1); fused with the apex carinae of the carina sterni (2). Mayr (2005a:28). See also Livezey and Zusi (2007a:1318); Bourdon et al. (2005:44).

139. Claviculae, craniocaudal breadth of extremitas omalis claviculae (epicleideum) relative to ventral portion of clavicle: subequal or slightly broader (0); significantly broader (1). Livezey and Zusi (2007a:1222).

140. Claviculae, curvature of base of processus omalis claviculae: grades smoothly into the body of the clavicle proximal to the coracoid articulation (0); processus omalis claviculae is offset caudodorsally from the curvature of the clavicular body by a distinct ‘kink’ at is base (1). Livezey and Zusi (2007a:1223).

141. Claviculae, relative craniocaudal elongation of processus omalis claviculae: short, articular faces for the coracoid and acromion process of the scapula not widely separated (0); elongate, articular facies widely separated (1). Livezey and Zusi (2007a:1224).

142. Claviculae, ventral incisure or sulcus between ventral edge of processus omalis claviculae and facies articularis acrocoracoidea: absent (0); present (1). Livezey and Zusi (2007a:1225).

143. Claviculae, extremitas omalis with strongly developed, laterally protruding facies articularis acrocoracoidea that articulates with a distinct ovoid facies articularis clavicularis of the coracoid: no (0); yes (1). Mayr (2005a:27). See also Mayr and Clarke (2003:62); Livezey and Zusi (2007a:1226); Bourdon et al. (2005:45).

144. Claviculae, fenesta (fenestra subacrocoracoidea claviculae) created by fusion of anterodorsal portion of processus omalis claviculae to coracoid: absent (0); present (1). Livezey and Zusi (2007a:1225, 1335).

145. Claviculae, elongate ventral sulcus on medial side of ventral edge of processus omalis claviculae: absent (0); present (1). New Character.

146. Claviculae, pneumaticity on ventral portion of processus omalis claviculae: absent (0); present (1). Modified from Livezey and Zusi (2007a:1227). See also Warheit (1990:FUR1).

147. Claviculae, craniocaudal curvature (in lateral aspect) of body of clavicle, exclusive of extremitas omalis claviculae: weakly to moderately convex anteriorly (0); strongly curved and convex, approaching subcircular form (1). Livezey and Zusi (2007a:1233).

148. Claviculae, mediolateral elongate sulcus (cisterna symphysialis) on dorsal portion of symphysialis claviculae: absent (0); present (1). Livezey and Zusi (2007a:1239).

149. Claviculae, orientation of medial surfaces of clavicles and synostosis interclavicularis: medial sufaces parallel and facing each other, synostosis interclavicularis caudodorsally directed (0); medial surfaces not parallel, directed craniomedially, synostosis interclavicularis dorsally directed (1). Warheit (1990:FUR2).

150. Claviculae, anterior separation of sternal articular facet, creating a pair of medially opposing facets anteriorly: absent (0); present (1). Livezey and Zusi (2007a:1241).

151. Claviculae, depressio symphysialis furculae: absent (0); present (1). Livezey and Zusi (2007a:1242).

152. Claviculae, shape of sternal articular facet in ventral (articular) aspect: simple spur or indescript facet (0); robust oval to upside-down heart shape (1); triangular, with apex elongated posteriorly and dorsally to create a craniocaudally elongate facet (2). New Character.

153. Claviculae, small, craniocaudally elongate tuberosity just lateral to the base of processus omalis claviculae, and dorsomedial to the coracoid articular facet: absent (0); present (1). New Character.

154. Claviculae, abrupt posterodorsal ‘kink’ in scapus claviculae near its connection with extremitas omalis sternalis, accompanied by anterolateral tuberosity and thickening of body of clavicle: absent (0); present (1). New Character.

155. Scapula, relative cranial extension of acromion: short, does not extend cranial to articular facies for the coracoid (0); elongate, extends well past articular facies for the coracoid (1). Livezey and Zusi (2007a:1245).

156. Scapula, shape of acromial process: blunt to rectangular process (0); extremely compressed dorsoventrally, elongate, finger-like morphology (1). New Character.

157. Scapula, pneumaticity associated with cranial border of scapula at base of acromion: absent (0); present (1). Livezey and Zusi (2007a:1249).

158. Scapula, area of origin for m. deltoideus major on the lateral face of the cranial end of the scapula, between the acromion process and humeral articulation: relatively unmarked or weakly convex (0); distinct concave fossa (1). Livezey and Zusi (2007a:1252).

159. Scapula, convexity of humeral articular facet of scapula: suplanar to concave fossa (0); convex coytle (1). Livezey and Zusi (2007a:1254).

160. Scapula, robust, mound-shaped tuberosity present on the lateral surface of the scapula: absent (0); present (1). New Character, but see Livezey and Zusi (2007a:1256) for a different interpretation.

161. Scapula, relative craniocaudal position of tuberosity (or unmarked area of insertion) for m. proscapulohumeralis: near or slightly cranial to midpoint of scapula (0); cranially located, closer to humeral articular facet than to midpoint of scapula (1). New Character.

162. Scapula, tuberosity associated with origin of m. subscapularis on ventromedial edge of cranial half of scapula: relatively unmarked or short linea or crest (0); craniocadually elongate tab-like crest, well-produced ventrally (1). New Character.

163. Scapula, tuberosity associated with origin of m. scapulotricipitis (= m. scapular head of triceps) on lateral surface of scapula, immediately caudodorsal to caudodorsal corner of humeral articular facet: unmarked or faint tuberosity (0); well-develped tuberosity (1). Livezey and Zusi (2007a:1259).

164. Scapula, main body of scapula expanded as a thin, sheet-like, blade: absent (0); present (1). Mayr (2005a:31).

165. Scapula, shape of caudal end of scapula: acuminate or weakly dorsoventrally expanded relative to scapular body (0); expanded dorsoventrally and spatulate in lateral or medial aspect (1). Livezey and Zusi (2007a:1264).

166. Scapula, degree of excavation on lateral surface of scapula at craniodorsal corner of origin of m. deltoideus major: relatively unmarked or weakly concave (0); well-developed concave fossa with clear margins (1). New Character.

167. Coracoid, relative curvature of cranial end of acrocoracoid process, with resepect to long axis of coracoid: little to slight lateral curvature (0); moderate medioventral curvature (1); extreme ventral curvature creating hamulate cranial end of acrocoracoid process (2). Livezey and Zusi (2007a:1268).

168. Coracoid, pneumaticity on caudal portion of acrocoracoid process, between humeral and clavicular articular facies: absent (0); present (1). Livezey and Zusi (2007a:1274, 1275).

169. Coracoid, development of sulcus associated with cranial border of impressio ligamenti acrocoracohumeralis: absent or weak (0); strong sulcus present (1). Livezey and Zusi (2007a:1276).

170. Coracoid, development of scapular articular facies: planar to weakly convex or concave facet (0); well-developed cup-shaped cotyle (1). Livezey and Zusi (2007a:1281).

171. Coracoid, shape of humeral articular facet: circular or weakly ovoid (0); dorsoventrally narrow and craniocaudally elongated (1). New Character.

172. Coracoid, facies articularis humeralis strongly protruding laterally at its anterior end, overhanging lateral border of processus acrocoracoideus: no (0); yes (1). Bourdon (2005:106); Warheit (1990:COR5).

173. Coracoid, development of procoracoid process: rudimentary or weakly developed (0); prominent, extending craniodorsally from base (1). Livezey and Zusi (2007a:1283).

174. Coracoid, relative orientation of apical tip of procoracoid process: extends primarily medially (0); extends significantly cranially as well as medially, approaching acrocoracoid process (1). Livezey and Zusi (2007a:1289).

175. Coracoid, shape of procoracoid process: low and rounded (0); spatulate (1); acuminate (2). New Character.

176. Coracoid, relative convexity of caudal portion of triosseal canal: flat to weakly concave (0); distinctly convex and swollen (1). New Character.

177. Coracoid, relative craniocaudal shape of facies articularis clavicularis: facet circular or nearly so in shape (0); facet oblong in shape, significantly longer craniocaudally than wide dorsoventrally (1). Warheit (1990:COR11).

178. Coracoid, relative orientation of facies articularis clavicularis: faces cranioventrally and medially (0); caudal (sternal) end of facet is strongly everted, enhancing the cranial and medial components to its orientation (1). New Character.

179. Coracoid, craniocaudally elongate sulcus on ventral surface of coracoid shaft, just caudal to furcular articular facies: absent (0); present (1). New Character.

180. Coraoid, development of pit for biciptial attachment (= impressio m. biceps brachii) lateral to clavicular articular facies: indistinct or weakly excavated (0); strongly excavated pit (1). Warheit (1990:COR6), and Van Tets (1988).

181. Coracoid, relative craniocaudal position of impression/pit for m. biceps brachii on acrocoracoid process: cranially, at or slightly above the midpoint of the facies articularis clavicularis (0); caudally, below the midpoint of the facies articularis clavicularis (1); significantly cranially, situated well above the facies articularis clavicularis (2). Warheit (1990:COR7).

182. Coracoid, foramen nervi supracoracoidei: absent (0); present (1). Mayr (2005a:29); Livezey and Zusi (2007a:1286).

183. Coracoid, tuberculum ancorae ligamentosa on medial edge of sternal end of coracoid: absent (0); present (1). Livezey and Zusi (2007a:1302).

184. Coracoid, extremitas sternalis, processus lateralis greatly elongated: no (0); yes (1). Mayr (2005a:30).

185. Coracoid, relative concavity of lateral border of lateral process: straight or weakly convex (0); distinctly concave or ‘notched’ (1). Livezey and Zusi (2007a:1308).

186. Coracoid, angle of the lateral margin of the lateral process relative to the long axis of the sternal articular facet: approximately 90 degrees (0); significantly obtuse (1). Warheit (1990:COR3).

187. Coracoid, strong buttress present just cranial to labrum internum of sternal articular facet: absent (0); present (1). Livezey and Zusi (2007a:1313).

188. Coracoid, craniocaudal postion of labrum internum relative to labrum externum (best viewed in medial to craniomedial aspect): craniocaudal positions of labri relatively equal (0); labrum internum significantly cranial in position, resulting in a caudodorsally inclined sternal articular facet (1). Livezey and Zusi (2007a:1314).

189. Coracoid, mediolateral position of labrum externum relative to labrum internum along sternal articular facet: labrum externum located in approximately the same mediolateral position or medially to labrum internum (0); labrum externum located laterally to labrum internum (1). New Character.

190. Coracoid, relative mediolateral width of labrum externum relative to dorsoventral height: wide and narrow (0); mediolaterally thin and dorsoventrally tall, forming a semilunate to triangular facet (1). Warheit (1990:COR1).

191. Coracoid, pneumatic foramen associated with sternal articular facies, just lateral to lateral edge of labrum internum: absent (0); present (1). Livezey and Zusi (2007a:1317).

192. Coracoid, intersection of anterior intermuscular line with labrum externum: intermuscular line intersects lateral portion of labrum externum (0); intermuscular line intersects labrum externum more medially, near midpoint of labrum externum (1). Siegel-Causey (1988:56).

193. Coracoid, relative positions of sternal (caudal) portion of anterior intermuscular line and craniomedial edge of sternocoraocoideus muscle scar on anterior surface of coracoid: two lines meet sternally near the sternal articular facet, leaving a small triangular gap of unfinished bone between them (0); two lines meet much more cranially, at or near the medial inflection of the anterior intermuscular line, no triangular gap present (1). Warheit (1990:COR2).

194. Coracoid, prominent tubercle located at intersection of anterior intermuscular line and labrum externum: absent (0); present (1). New Character (see Van Tets et al. 1988).

195. Coracoid, small triangular articular facies on caudodorsal corner of medial process of coracoid for articulation with opposing coracoid: absent (0); present (1). New Character. See also Livezey and Zusi (2007a:1331).

196. Coracoid, mediolateral length of the sternal articular facet relative to the mediolateral length of the caudal edge of the coracoid from angulus medialis to the lateral edge of the muscle scar for m. sternocoracoideus: much greater than 50% (0); close to 50% (1). Warheit (1990:COR4).

197. Coracoid, relative posterior convexity of the edge of bone between the crista articularis sternalis and the lateral process of the coracoid, viewed in ventral or dorsal aspect: straight, or weakly concave (0); strongly concave, forming a curvilinear arc between the sternal articular crest and the lateral process of the coracoid (1). New Character.

198. Humerus, proximal end of humerus with a deep, rounded head and ventrally directed caput humeri: no (0); yes (1). Mayr (2005a:36).

199. Humerus, well-developed caudolaterally facing tuberosity distal to tuberculum dorsale on proximal humerus: absent (0); present (1). New character.

200. Sulcus transversus on anterior face of proximal end of humerus very deep, long, and rectangular: no (0); yes (1). Mayr (2005a:38). See also Warheit (1990:HUM8), and Clarke (2004:111).

201. Humerus, tuberculum m. pectoralis superficialis, pars deep (see Owre, 1967:15, Fig. 13) depth: anterior surface of humeral shaft medial and distal to tuberculum relatively smooth, without depression (0); medial and distal edge of tuberculum slightly raised, with groove-like depression along its edges on the humeral shaft (1); deep groove medial and distal to tuberculum, with distal portion of tuberculum hypertrophied as a round swelling (2). Warheit (1990: HUM6), see also Livezey and Zusi (2007a:1400).

202. Humerus, impressio m. coracobrachialis cranialis: flat to shallow, oval muscle scar, not distinctly depressed (0); deeply excavated oval muscle scar (1); deeply excavated proximally, with distal end rugose, with well-rimmed distal edge, and raised anteriorly and proximally as a distinct tuberosity (2). Warheit (1990:HUM7).

203. Humerus, proximomedial (proximoventral) border of fossa m. coracobrachialis at juncture with intumescentia: low and indistinct (0); raised as a sharp proximodistally oriented ridge (1). Bourdon (2005:113).

204. Humerus, anterior surface of crista bicipitalis (= “intumescentia”): inflated and bulbous (0); weakly convex or planar (1). Livezey and Zusi (2007a:1405).

205. Humerus, distinct proximodistally elongate, rugose tuberosity present distal to bicipital crest, and separated from it by a shallow cleft: absent (0); present (1). New character.

206. Humerus, relative development and shape of deltopectoral crest: slightly protruding, low and rounded (0); strongly protruding and triangular (1); extremely reduced (2). Mayr (2005a:39). See also Livezey and Zusi (2007a:1374).

207. Humerus, ala subtubercularis (sensu Livezey and Zusi 2007a:p224) on proximal end of deltopectoral crest, just anterior and distal to dorsal tubercle: absent (0); present (1). Bourdon (2005:80); Livezey and Zusi (2007a:1391).

208. Humerus, margin of deltopectoral crest runs roughly parallel to shaft of humerus: no (0); yes (1). Livezey and Zusi (2007a:1380); Bourdon (2005:113).

209. Insertion for m. latissimi dorsi (mainly pars caudalis) on posterior side of humerus: scar situated along midline (0); scar displaced laterally (dorsally) toward deltopectoral crest (1). Warheit (1990:HUM4; HUM5) and Livezey and Zusi (2007a:1441).

210. Humerus, shape of shaft cross-section: elliptical and uncompressed (0); subelliptical with anteroposterior (craniocaudal) compression (1); extremely anteroposteriorly compressed (2). Livezey and Zusi (2007a:1439).

211. Humerus, sulcus m. scapulotricipitis: dorsal (lateral) and ventral (medial) ridges relatively equally developed (0); ventral ridge much more well-developed in caudal and proximal extent (1). Warheit (1990:HUM12). See also Livezey and Zusi (2007a:1488).

212. Humerus, shape of process flexorius (= “entepicondyle”) in medial (ventral) aspect: low and rounded, crescent-shaped (0); quadrangular and tab-shaped, cranial and caudal borders do not grade smoothly proximally into distal humerus (1). New character.

213. Humerus, distal extent of process flexorius (= “entepicondyle”): low, does not extend to level of ventral condyle (0); extends to or slightly beyond level of ventral condyle (1); extends caudally and distally significantly beyond level of ventral condyle (2). Livezey and Zusi (2007a:1476).

214. Humerus, conspicuous, proximodistally elongate triangular inflation of distal humerus caudal and proximal to process flexorius: absent (0); present (1). Livezey and Zusi (2007a:1477).

215. Humerus, depth of fossa m. brachialis on anterior surface of distal humerus: shallow or weakly excavated (0); deeply excavated and pit-shaped (1). Livezey and Zusi (2007a:1456). See also Warheit (1900:HUM16).

216. Humerus, orientation of long-axis of fossa m. brachialis on anterior surface of distal humerus: extends proximolateral to distomedial (0); proximodistal (1).

217. Humerus, distinct ridge extending significantly proximally up humeral shaft from proximal end of tuberculum supracondylare ventrale: absent (0); present (1). Siegel-Causey (1988:83).

218. Humerus, fossa olecrani shape in distal aspect: absent or weakly developed (0); shallow, triangular-shaped (1); deeply excavated and bowl-shaped (2); extremely deep, well-rimmed, circular fossa (3). Warheit (1990: HUM1); Livezey and Zusi (2007a:1482).

219. Humerus, fossa olecrani pneumaticity: absent or extremely small pneumatic foramina scattered on surface (0); pneumatic with one or more foramina on the lateral wall of the olecranon fossa, undercutting the medial edge of sulcus tendinis m. scapulotricipitalis (1); extensive pneumaticity on the proximal wall of the olecranon fossa (2). Warheit (1990: HUM3), Livezey and Zusi (2007a:1485).

220. Distal end of humerus strongly flattened and ventrally protruding, sulci scapulotricipitalis et humerotricipitalis forming two deep furrows and shifted towards ventral margin of bone: no (0); yes (1). Mayr (2005a:37).

221. Humerus, shape of tuberculum supracondylare ventrale in medial (ventral aspect): relatively flat or planar (0); convex dorsally, particularly on the distal half of the tuberculum (1); distal half of tuberculum distinctly concave, giving the tuberculum a triangular, ‘pointed’ appearance in medial (ventral) aspect (2). Warheit (1990:HUM17).

222. Humerus, orientation of tuberculum supracondylare ventrale in anterior aspect: faces primarily anteriorly (0); canted laterally (dorsally) and faces anterolaterally (1); canted medially (ventrally) and faces anteriormedially (2). Warheit (1990:HUM18).

223. Humerus, relative location of muscle scar for insertion of M. pronator superficialis (= “m. pronator brevis”): posterior to tuberculum supracondylare ventrale (0); posterior and distal to tuberculum supracondylare ventrale and developed as a small tubercule (1); only slightly posterior, and proximal to tuberculum supracondylare ventrale (2). New Character.

224. Humerus, proximal extremity of condylus dorsalis: slight medial (ventral) to proximomedial curve (0); strongly medially curved at a right angle to the distal base of condylus dorsalis, giving the condyle a ‘hook’-like appearance in anterior aspect (1). Bourdon (2005:118).

225. Humerus, pneumaticity associated with distal humerus, proximal to distal condyles and distal to fossa m. brachialis: absent (0); present (1). Livezey and Zusi (2007a:1459).

226. Humerus, development of epicondylus dorsalis (= “ectepicondyle”): weak and relatively flat or planar laterally (dorsally) (0); well-developed and convex laterally, forming a rounded, crescent-shaped tubercle in anterior aspect (1). Livezey and Zusi (2007a:1461).

227. Humerus, development of processus supracondylaris dorsalis: absent or weakly developed (0); large and strongly protruding (1). Mayr (2005a:40); Livezey and Zusi (2007a:1467).

228. Humerus, position of processus supracondylaris dorsalis relative to epicondylus dorsalis: conjoined, little differentiation between processes (0); tuberculum supracondylaris dorsalis distinctly proximal to epicondylus dorsalis (1). Livezey and Zusi (2007a:1469).

229. Ulna/Radius, pronounced craniocaudal compression resulting in sublaminate shape: abesnt (0); present (1). Mayr (2005a:41); Livezey and Zusi (2007a:1516, 1544).

230. Ulna, papillae remigales caudales dorsales of ulna: absent or present only as small impressions (0); present as prominent tuberosities (1). Modified from Livezey and Zusi (2007a:1521).

231. Ulna, trochlea humeroulnaris: absent, or if present, comprising only pars ulnaris as origin of m. expansor secundariorum; m. flexor carpi ulnaris reduced to tendon (0); present, comprising partes humeralis proprius, humeralis accessoria, et ulnaris (1). Livezey and Zusi (2007a:1739).

232. Ulna, craniocaudal shape (primarily craniodcaudal width near proximal end) of impressio m. brachialis: proximodistally elongate oval-shaped (0); proximodistally elongate, slot-shaped (1). Warheit (1990:ULN1); Livezey and Zusi (2007a:1502).

233. Ulna, pneumatic foramen or foramina in proximal end of impressio m. brachialis: absent (0); present (1). Warheit (1990:ULN2); Livezey and Zusi (2007a:1503).

234. Ulna, development of sulcus radialis (= “distal radial depression”, “depressio radialis”): present, shallow (0); present, deeply incised, particularly at distal end (1); absent, homologous region cranially convex (2). Livezey and Zusi (2007a:1524); Warheit (1990:ULN3).

235. Ulna, pneumatic foramina on caudal surface of tuberculum carpale (= “carpal tuberosity”): absent (0); present (1). Warheit (1990:ULN4). See also Livezey and Zusi (2007a:1535).

236. Ulna, shape of tuberculum carpale: sloping proximal border, ventrodistal edge of tuberculum carpale relatively straight, typically at a right angle (or slightly less) relative to the long axis of the ulna (0); sloping proximal border, ventrodistal distinctly curvilinear, extending proximally from the distal base, with a tab-like tip (1); sloping proximal border, ventrodistal border distinctly concave distally, ending in a distally projected point (2); proximal and distal borders subparallel, join with ventral tip of tubercle at sharp angles giving it a quadrangular morphology (3). Warheit (1990:ULN5); Livezey and Zusi (2007a:1533, 1536).

237. Ulna, profile of olecranon process in proximal aspect: mound-shaped, with ventral curve at caudal end (0); craniocaudally elongated and dorsoventrally compressed, particularly at insertion for m. humerotriceps, resulting in a reverse “S”-shaped profile in proximal aspect (1). Bourdon (2005:82).

238. Ulna, development of sulcus tendineus (m. flexor carpi ulnaris) (= “humero-ulnar depression”): sulcus relatively flat or weakly concave, does not undercut olecranon and ventral cotyla proximocranially (0); sulcus deeply indented, undercutting olecranon and ventral cotyla proximocranially (1). Warheit (1990:ULN7). See also Livezey and Zusi (2007a:1512), Bourdon (2005:119), and Siegel-Causey (1988:86).

239. Ulna, shape of ulnar shaft: slightly to strongly cranially bowed, particularly at proximal end (0); relatively proximodistally elongated and straight, little to virtually no curvature (1). Bourdon (2005:59); Livezey and Zusi (2007a:1490, 1519).

240. Ulna, prominent, dorsoventrally compressed, sublaminated crest extending distally from craniodorsal edge of processus cotylaris dorsalis: absent (0); present (1). Livezey and Zusi (2007a:1493).

241. Ulna, well-defined sulcus intercondylaris and condylus ventralis: absent (0); present (1). Bourdon (2005:15).

242. Ulna, pointed process (= “tuberculum cristae”) at caudal edge of crista intercotylaris: absent (0); present (1). Bourdon (2005:88); Livezey and Zusi (2007a:1498).

243. Ulna, proximodorsal corner of incisura radialis undercuts cranial edge of cotyla dorsalis: no (0); yes (1). Livezey and Zusi (2007a:1505).

244. Ulna, pneumaticity associated with incisura radialis: absent, or very minute pores (0); present, large pneumatic foramina (1). Livezey and Zusi (2007a:1507, 1508).

245. Ulna, shape of impressio ligamenti collateralis ventralis: spherical to ovate (0); distinctly proximodistally elongate (1). Livezey and Zusi (2007a:1514); Bourdon (2005:65).

246. Ulna, depression for insertion of m. humerotriceps on proximodorsal edge of olecranon process: creates a concave saddle-shape (0); area flat to convex, with no marked depression (1). New Character.

247. Ulna, labrum condyli dorsalis (= “semilunate ridge”) of condylus dorsalis: grades into ulnar shaft rather smoothly at its proximal edge along articulation with os carpi ulnaris (0); extends abruptly at a near perpendicular angle at its proximal juncture with the ulnar shaft (1). Livezey and Zusi (2007a:1528).

248. Ulna, relative proximodistal postions of distal condyles: condyles subequal in distal extent (0); condylus dorsalis significantly proximal to condylus ventralis (1); condylus dorsalis extended distally to condylus ventralis (2). Livezey and Zusi (2007a:1530); Bourdon (2005:74).

249. Ulna, pneumatic foramina caudoventral to labrum condyli dorsalis and proximal to sulcus intercondylaris: absent (0); present (1). New character.

250. Ulna, pronounced saddle-shaped depression on ventral side of condylus ventralis, cranial to sulcus intercondylaris and caudal to tuberculum carpale, giving the ventral condyle a bilobate appearance with a well-separated, pointed proximal “lobe”: absent (0); present (1). New character.

251. Ulna, relative development of tuberculi retinaculi: nearly indiscernible, or very small (0); robust, flange-like processes (1). New character.

252. Radius, shape of tuberculum bicipitale radii: rounded, to proximodistally elongate tuberosity (0); rounded tuberosity proximally, overhanging oval-shaped depression or fovea distally (1). Livezey and Zusi (2007a:1540). See also Bourdon (2005:33).

253. Radius, robust tubercle on proximal radius, caudodorsal to biceps tubercle: absent (0); present (1). New Character.

254. Radius, proximally concave indentation in edge of humeral cotyle created by robust tubercle on proximal radius caudodorsal to biceps tubercle: absent (0); present (1). New Character.

255. Radius, development of cranioventral tuberosity (“capital tuberosity”) on proximal end, opposite of biceps tubercle: relatively unmarked, low tuberosity (0); extremely robust and protruding cranioventrally, tab-shaped outline in craniodorsal aspect (1). New Character.

256. Radius, distinct sulcus for insertion of m. brachialis on dorsal surface of proximal radius: absent (0); present (1). Livezey and Zusi (2007a:1541).

257. Radius, margo cranialis, facies ventralis, jugum aut ala cranialis et sulcus muscularis (sensu Livezey and Zusi, 2007a), latter primarily supporting insertio m. deltoideus, pars propatagialis, caput craniale (pars longus): absent (0); present (1). Livezey and Zusi (2007a:1542).

258. Radius, shape of tuberculum aponeurosis ventralis: inconspicuous or low and rounded (0); promiment proximodistally elongate rounded ridge (1); prominent and extending caudally as a peg-like process (2); relatively robust, distally located mound-shaped tubercle (3). Livezey and Zusi (2007a:1555).

259. Radius, small, distally directed tuberosity at cranioventral border of sulcus tendineus, with small fossa located distal and slightly caudally to it: absent (0); present (1). Bourdon (2005:75).

260. Radius, curvature of cranioventral border of distal radius and juncture with expanded distal end: relatively little curvature, cranioventral tuberosity of distal end expands abruptly from radial shaft (0); pronounced cranioventrally concave curvature, cranioventral tuberosity of distal end grades smoothly from radially shaft (1). New character.

261. Os carpi ulnare flattened, with large caudal expansion: no (0); yes (1). Mayr (2005a:42). See also Livezey and Zusi (2007a:1565, 1571).

262. Os carpi ulnare, sulcus tendineus radioulnaris (sensu Livezey and Zusi, 2007a) on proximoventral surface of ventral ramus (sensu Livezey and Zusi, 2007a; = “crus longum”): absent or indistinct (0); present, open sulcus (1), present, closed proximally (2). Livezey and Zusi (2007a:1566).

263. Os carpi ulnare, pneumatic foramina in incisura metacarpalis: absent (0); present (1). Livezey and Zusi (2007a:1567).

264. Os carpi ulnare, distinct raised tuberosity (= “processus muscularis” of Livezey and Zusi, 2007a:1573, “tuberculum at insertion of ligamentum humerocarpale” of Mayr and Clarke, 2003:88), at proximal end of incisura metacarpalis: absent or indistinct (0); present, prominent mound or tuberosity (1). Livezey and Zusi, 2007a:1573.

265. Os carpi ulnare, proximal apex of facies for articulation with ulna: rounded, not deflected (0); tab-shaped proximalmost portion deflected proximoventrally at an angle almost perpendicular to rest of articular facies (1). New Character.

266. Os carpi ulnare, relative length of ventral ramus: subequal to or shorter than dorsal ramus (0); conspicuously longer than dorsal ramus (1). Livezey and Zusi (2007a:1570).

267. Os carpi ulnare, small proximal shelf-like crest overhanging caudoproximal end of sulcus tendineus radioulnaris: absent (0); present (1). New character.

268. Os carpi radiale, pneumatic foramina on distal surface: absent (0); present (1). Livezey and Zusi (2007a:1563).

269. Os carpi radiale, concavity of distal surface: subplanar to weakly concave (0); broad, well-developed concave excavation across most of distal surface (1). New character.

270. Os carpi radiale, development of pully-like groove on ventral surface that extends toward radial cotyle and sulcus tendineus of radius: weakly to moderately developed facet or groove (0); strongly developed and pulley-shaped, with high, sharp edges (1). New character.

271. Os carpi radiale, development of groove on distal and ventral suface of radiale, (located caudal to groove described in character 201): weakly defined sulcus, primarily located on ventral surface of radiale, slightly extending onto distal surface (0); extremely deep, pulley-like groove, extends all the way across distal surface of radiale toward ulnar facet (1). New character.

272. Ossa metacarpalia, degree of fusion and proximodistal extent of metacarpal I (alulare): distinguishable, extending no farther distal than symphysis intermetacarpalis proximalis (0); diminutive, synostotic with metacarpal II and proximal digit I (1); distinguishable, comparatively elongate, extending significantly distad to symphysis intermetacarpalis proximalis (2). Livezey and Zusi (2007a:1580; see also 1749, 1751).

273. Ossa metacarpalia, width of spatium intermetacarpale: wide, metacarpals II and III clearly distinguished (0); narrow, metacarpals II and III nearly fused (1). Livezey and Zusi (2007a:1581).

274. Ossa metacarpalia, convexity of proximal end of caudal surface of metacarpal III shaft: slightly convex caudally (0); flat to weakly concave caudally (1); concave caudally, creating well-defined sulcus on proximal end of metacarpal III shaft (2). Livezey and Zusi (2007a:1590).

275. Ossa metacarpalia, relative distal extent of metacarpals II and III: metacarpal II equal to or longer than metacarpal III (0); metacarpal II shorter than metacarpal III (1). Livezey and Zusi (2007a:1591).

276. Ossa metacarpalia, proximodistal extent of sulcus tendineus (= sulci tendinorum mm. extensor longus digiti majoris and extensor digitorum communis) on dorsal surface of metacarpal II: sulcus absent (0), sulcus limited to distal half of metacarpal II (1), sulcus extending across length of metacarpal II (2). Livezey and Zusi (2007a:1603).

277. Ossa metacarpalia, relative length of os metacarpale alulare to digitus alularis: os metacarpale alulare smaller (0); os metacarpale alulare and digitus alularis approximately equal (1). Livezey and Zusi (2007a:1619).

278. Ossa metacarpalia, relative caudal extension of ventral and dorsal rims of trochlea carpalis at caudal border of trochlea: ventral rim extends slightly further caudally than ventral rim (0); ventral rim extends conspicuously further caudally than dorsal rim (1). New character. See Livezey and Zusi (2007a:1626) for a similar, but distinct character.

279. Ossa metacarpalia, pneumatic foramen in fossa infratrochlearis ventralis of ventral face of proximal carpometacarpus: absent (0); present (1). Livezey and Zusi (2007a:1640); Warheit (1990:CMC4).

280. Ossa metacarpalia, development of fovea carpalis caudalis: shallow to moderately deep depression (0); markedly deep, ovate depression (1). Livezey and Zusi (2007a:1644); Warheit (1990:CMC2,3).

281. Ossa metacarpalia, pneumatic foramina in fovea carpalis caudalis: absent (0); present (1). Livezey and Zusi (2007a:1646); Warheit (1990:CMC1).

282. Ossa metacarpalia, development of fovea carpalis cranialis: indistinct to slightly excavated (0); deep and well-excavated, convace ventrally concave fossa (1). Livezey and Zuis (2007a:1647); Siegel-Causey (1988:90); Warheit (1990:CMC6).

283. Ossa metacarpalia, pneumatic foramina in fovea carpalis cranialis: absent (0); present (1). Livezey and Zusi (2007a:1648); Warheit (1990:CMC5).

284. Ossa metacarpalia, os metacarpale minus (III), facies, ventralis, pars proximalis, eminentia retinaculum flexorium of aponeurosis ventralis (sensu Livezey and Zusi, 2007a): absent, or weak impression or eminence (0); present as distinct tuberculum or crest (1). Livezey and Zusi (2007a:1652).

285. Ossa metacarpalia, pneumatic foramina in synostosis metacarpalis distalis: absent (0); present (1). Warheit (1990:CMC10).

286. Manus, number and form of phalanges of digit I (aulula): two, with ungual phalanx vestigial (0); two, proximal phalanx robust and proximally hamulate, distal phalanx sine unguis (1); one, ungual phalanx absent (2). Livezey and Zusi (2007a:1677).

287. Manus, pneumaticity associated with manual phalanges: absent (0); present (1). Livezey and Zusi (2007a:1682, 1730).

288. Manus, neurovascular foramen caudal to caudal margin of proximal end of proximal phalanx of digit I: absent (0); present (1). Livezey and Zusi (2007a:1694).

289. Manus, degree of dorsoventral compression of shaft of proximal phalanx of digit II: absent to moderate, shaft retains robust pila cranialis (0); strongly dorsoventrally flattened, and craniocaudally expanded, lacking pila cranialis (1). Livezey and Zusi (2007a:1707).

290. Manus, development and shape of dorsal tubercle (= “tuberculum dorsalis tendinis”) on proximal articular face of the proximal phalanx of digit II: absent to weakly developed, rectangular and tab-shaped (0); prominent, extending relatively further caudodorsally, and sphere-shaped (1). Livezey and Zusi (2007a:1711).

291. Manus, development and shape of caudodistal tubercle on distal end of II-1: tapers to a point, not extending distally far past distal articular end of II-1 (0); keel-like, extending signifcantly distally or caudodistally from caudal margin of II-1 (1). Livezey and Zusi (2007a:1722).

292. Manus, craniocaudal curvature of distal portion of caudal margin of II-1: caudally convex, smoothly curved with rest of caudal margin to weakly concave (0); deep, and proximodistally elongate caudal concavity proximal to the caudodistal tubercle (1). New character.

293. Manus, proximodistally elongate fenestra on the distal third of the blade of II-1: absent (0); present (1). New Character. See Olson (1977:24).

294. Manus, unguis digit majoris: present (0); absent (1). Livezey and Zusi (2007a:1725).

295. Manus, development of tuberosity on caudal border of III-1: absent to weakly developed (0); robust, and caudally expanded, with hook-like tip (1). Livezey and Zusi (2007a: 1729).

296. Pelvis, extreme bilateral compression of ossa coxae resulting in acetabulum positioned immediately lateral or lateroventral to columna synsacralis: absent (0); present (1). Livezey and Zusi (2007a:1766, 1860, 1924).

297. Pelvis, pneumaticity associated with caudomedial (contra-articular) face of the antitrochanter: absent (0); present (1). Livezey and Zusi (2007a:1777).

298. Pelvis, strong ridge (= jugum supra-antitrochantericus of Livezey and Zusi, 2007a:1780) running between crista dorsolateralis ilii and antitrochanter on dorsal surface of ilium: absent (0); present (1). Livezey and Zusi (2007a:1780).

299. Pelvis, relative concavity of concavitas infracristalis: moderately concave (0); shallow to planar, concavitas near obsolete (1). Livezey and Zusi (2007a:1782).

300. Pelvis, craniocaudal lenth of foramen ilioischiadicum relative to total length of foramen plus concavitas infracristalis: foramen between 1/3 and 1/2 of total length (0); foramen elongate, longer than 1/2 of total length (1); foramen abbreviate, shorter than 1/3 of total length (2). Livezey and Zusi (2007a:1790).

301. Pelvis, fossa renalis on ventral surface of pelvis: present (0); absent (1). Livezey and Zusi (2007a:1793).

302. Pelvis, portioning of fossa renalis into pars ischiadica (cranially) and pars pudenda (caudally) by laterally and ventrally extensive costal processes of the synsacral vertebrae and crista iliaca intermedia: present (0); absent (1). Livezey and Zusi (2007a:1794).

303. Pelvis, number of costal processes of synsacral vertebrae that participate in division of fossa renalis (when division is present): multiple (at least 3), with anteriormost costal process being the most ventral, and more posterior processes being less ventrally inclined along a smooth, planar transition (0); a single (or two narrowly spaced), well-ventrally placed costal process divides fossa renalis (1). New Character.

304. Pelvis, broad fossa or foramina and pneumaticity associated with the cranial portion of fossa renalis, pars pudenda (or homologous region): absent (0); present (1). Livezey and Zusi (2007a:1798).

305. Pelvis, fossa renalis, recessus caudalis fossae (= recessus iliacus of Baumel, 1979): absent (0); present (1). Livezey and Zusi (2007a:1799).

306. Pelvis, small rimmed and pneumatic recess (“fossa postrenalis” of Livezey and Zusi, 2007a:1803) on ventromedial face of postacetabular blade of ilium, caudal to pila renalis (when present): absent (0): present (1). Livezey and Zusi (2007a:1803).

307. Pelvis, mediolateral width across processes margines caudalis relative to mediolateral with of caudal end of synsacrum: moderate, between 1.0 and 2.0 (0); wide, greater than 2.0 (1). Livezey and Zusi (2007a:1805).

308. Pelvis, tuberculum preacetabulare: present (0); absent (1). Mayr and Clarke (2003:93; Fig. 8); Livezey and Zusi (2007a:1810).

309. Pelvis, spacing of crista iliacae dorsales on preacetabular portion of ilia: closely appressed or fused across the midline (0); separated, exposing fenestrae intertransverariae between crista iliacae dorsales (1). Livezey and Zusi (2007a:1814, 1888, 1949).

310. Pelvis, cranial margin of facies dorsalis of preacetabular ilia multilobate, wih semilaminate subdivisions defining a composite ala near perpendicular to crista spinosa synsacri: absent (0); present (1). Livezey and Zusi (2007a:1820).

311. Pelvis, pneumaticity associated with lateral face of the preacetabular ilium, immediately cranial to the acetabulum: absent (0); present (1). Livezey and Zusi (2007a:1821).

312. Pelvis, orientation of preacetabular process of ilium relative to transverse plane of synsacrum: oblique, dorsal margin of ilium located medial and dorsal to lateral margin. (0); subhorizontal, dorsal margin of ilium located medial, and only slightly dorsal to lateral margin (1). Livezey and Zusi (2007a:1823).

313. Pelvis, curvature of the cranial edge of the preacetabular process of the ilium: convex cranially (0); convex cranially with a single shallow and broad concave indentation in the middle (1). Livezey and Zusi (2007a:1825).

314. Pelvis, extreme lateral expansion of cranial end of preacetabular process of ilium, coupled with reduction or “waisting” of preacetabular process in region just cranial to acetabulum: absent (0); present (1). Livezey and Zusi (2007a:1828).

315. Pelvis, bilaterally symmetrical cranial spines (= “spina supra-transversalis veterbrae of Livezey and Zusi, 2007a:1832) emerging from cranial border of preacetabular process of ilium, dorsal to synsacral transverse processes: absent (0); present (1). Livezey and Zusi (2007a:1832).

316. Pelvis, interacetabular width relative to synsacral length: greater than 1/2 (0); between 1/2 to 1/3 (1); approximately 1/4 to 1/5 (2); approximately 1/6 (3). Livezey and Zusi (2007a:1845).

317. Pelvis, large, circular fenestrae present lateral to (and paired with) medial fenestrae intertransversae synsacrales: absent (0); present (1). Livezey and Zusi (2007a:1851).

318. Pelvis, postion of vertex craniolateralis ilii relative to antitrochanter: caudodorsal (0); dorsal (1). Livezey and Zusi (2007a:1864).

319. Pelvis, morphology of vertex craniolateralis on crista iliaca dorsolateralis: low, rounded corner (0); raised tuberculum at vertex (1); vertex everted laterally and raised dorsally as tab-shaped, ventrally concave crest (2). Livezey and Zusi (2007a:1862, 1865, 1884).

320. Pelvis, caudal extension of spina dorsolateralis ilii relative to processus terminalis ischii: subequal or former slightly shorter than latter (0); former substantially shorter than latter (1); former greater than latter (2). Livezey and Zusi (2007a:1868, 1892, 1914).

321. Pelvis, orientation of caudal extension of spina dorsolateralis ilii: extends straight caudally, or nearly so (0); significant ventral curvature (1) extends caudodorsally at an angle near 45 degrees (2). Livezey and Zusi (2007a:1869, 1872).

322. Pelvis, mediolateral position of spina dorsolateralis ilii relative to processus terminalis ischii in dorsal perspective: spine located distinctly medially to terminal process of ischium in dorsal aspect, such that postacetabular portion of ilium faces slightly dorsolaterally (0); spine located immediately dorsal to terminal process of ischium or nearly so, such that both processes line up in dorsal aspect, and postacetabular portion of ilium faces nearly strictly laterally (1). Livezey and Zusi (2007a:1870).

323. Pelvis, lateral crest on spina dorsolateralis ilii (= “cristula lateralis spinae ilii” of Livezey and Zusi, 2007a:1874): absent (0); present (1). Livezey and Zusi (2007a:1874).

324. Pelvis, craniocaudal length of preacetabular process of ilium relative to postacetabular process: approximately subequal (0); preacetabular process significantly shorter (1); preacetabular process significantly longer. Livezey and Zusi (2007a:1890).

325. Ischium, orientation of caudal end of ischium: extends caudally or caudoventrally (0); significant ventral curvature, beginning near midpoint and particularly at terminal end (1). Livezey and Zusi (2007a:1899, 1904, 1923).

326. Ischium, caudal extent of pubis relative to ischium: subequal (0); pubis significantly longer than ischium (1). Livezey and Zusi (2007a:1901, 1915).

327. Ischium, abrupt narrowing of ischial shaft past midpoint, but well before terminus of shaft: absent (0); present (1). Livezey and Zusi (2007a:1907).

328. Pubis, relative convexity of dorsal margin of pubic shaft in lateral aspect: straight to concave (0); distinctly convex (1). Livezey and Zusi (2007a:1928).

329. Pubis, distinct dorsally projecting tuberculum associated with foramen obturatum: absent (0); present (1). Livezey and Zusi (2007a:1930).

330. Pubis, middle of pubic shaft distinctly reduced in diameter relative to proximal and distal portions: absent (0); present (1). Livezey and Zusi (2007a:1932).

331. Pubis, relative dorsoventral expansion of terminal end of pubis (apex pubis): little or no expansion (0); distinct expansion producing spatulate terminal end (1). Livezey and Zusi (2007a:1940).

332. Pubis, distinct ventral “kink” in pubic shaft near terminal end, resulting in an oblique angle formed between the caudoventrally directed apex pubis and the body of the pubis in lateral aspect: absent (0); present (1). Livezey and Zusi (2007a:1945).

333. Pubis, extensive fusion between distal pubis and distal ischium: absent (0); present (1). Livezey and Zusi (2007a:1952, 1956).

334. Femur, orientation of femoral head with respect to proximodistal axis of femur: 90º or nearly so (0); significantly greater than 90º (1). Livezey and Zusi (2007a:1968).

335. Femur, orientation of fovea ligamenti capitis on femoral head: fovea faces proximomedially (0); fovea directed almost strictly proximally (1). Warheit (1990:FEM5).

336. Femur, relative craniocaudal compression of trochanter femoris: little to none, trochanter distinctly broader craniocaudally than caput femoris (0); prominent, craniocaudal breadth of trochanter subequal to that of caput femoris (1). Livezey and Zusi (2007a:1967).

337. Femur, large, circular pneumatopore on craniomedial side of trochanter femoris: absent (0); present (1). Livezey and Zusi (2007a:1976); Warheit (1990:FEM4); Mayr and Clarke (2003:98).

338. Femur, lateral surface of trochanter femoris deeply excavated: absent (0); present (1). New Character.

339. Femur, relative proximodistal position of insertion scar for m. obturator medialis on the lateral margin of trochanter femoris: distal, does not extend onto the proximal edge of trochanter femoris (0); proximal, extends slightly onto the proximal edge of trochanter femoris, depressing the lateral margin slightly medially, and giving the lateral margin of trochanter femoris an irregular outline in proximal aspect (1). Warheit (1990:FEM2).

340. Femur, laterally everted, tab-like tuberosity associated with insertion scar of m. iliotrochantericus medialis: absent (0); present (1). New Character, though see Siegel-Causey (1988:113, 119).

341. Femur, development of caudal margin of facies articularis antitrochanterica: low, linear caudal edge (0); robust edge, distinctly everted caudally and extending beyond facies caudalis (1). Livezey and Zusi (2007a:1975).

342. Femur, relative proximal prominence of crista trochantericus: high, extends past proximal extent of femoral head (0); low, subequal to or does not extend past proximal extent of femoral head (1). Livezey and Zusi (2007a:1978, 1997).

343. Femur, cranial convexity of femoral shaft in lateral aspect: straight, or very weakly convex (0); significantly bowed and convex cranially (1). Livezey and Zusi (2007a:2000).

344. Proximodistally elongate muscle scar for m. flexor ischiofemoralis, connecting or approaching distally the tuberosity for insertion of m. caudofemoralis: absent (0); present (1). New character.

345. Femur, proximodistal orientation of linear, distal portion of scar for m. ischiofemoralis insertion on lateral/posterior femoral shaft: scar extends straight, or nearly straight distally, terminating on the posterolateral to slightly posterior side of the femoral shaft (0); scar curves posteriorly moving distally, and terminates on the posterior side of the femoral shaft (1). New character.

346. Femur, relative craniocaudal position of insertion scar for m. ischiofemoralis on the lateral surface of the femur: caudally, at or near the caudolateral edge of the femoral shaft (0); distinctly cranially, at, or cranial to, the middle of the lateral face of the femoral shaft (1). Warheit (1990:FEM3).

347. Femur, relative concavity of medial edge of femoral shaft in caudal aspect: medial edge straight (0); medially edge distinctly concave in caudal aspect (1). Warheit (1990:FEM6).

348. Femur, development of trochlea fibularis on lateral condyle of distal femur: well-defined (0); poorly-defined, and nearly absent (1). New Character.

349. Femur, mediolateral breadth of trochlea fibularis on lateral condyle of distal femur: moderate, subequal to breadth of medial condyle (0); extremely wide, broader than width of medial condyle (1). Livezey and Zusi (2007a:2017); Warheit (1990:FEM1).

350. Femur, orientation of lateral edge of trochlea fibularis in caudodistal aspect: edge relatively straight (0); edge has distinct medial inflection at its craniodistal end, just caudolateral to fovea tendineus m. tibialis cranialis, caused by caudal extension of faint muscle scar on distalmost end of lateral surface of lateral condyle (1). New Character.

351. Femur, proximal ridge and associated distomedial fossa of medial prominence of external condyle: ridge absent or low and weakly developed, fossa not deeply excavated (0); ridge very robust, fossa large and deeply excavated (1). Siegel-Causey (1988:124); Livezey and Zusi (2007a:2023).

352. Femur, large ovate accessory subfossa (senus Livezey and Zusi, 2007a:2039) in sulcus patellaris: absent (0); present (1). Livezey and Zusi (2007a:2039).

353. Femur, development of medial and lateral edges (the cranioproximal portions of the medial and lateral condyles, respectively) of sulcus patellaris: rounded, but robust, producing well-defined sulcus (0); extremely sharp and ridge-like, producing pulley-like sulcus (1); low and weakly developed, sulcus not strongly differentiated (2). New Character (though see Livezey and Zusi, 2007a:2041).

354. Femur, well-developed, proximodistally elongate crest/tubercle on the craniolateral edge of the distal femoral shaft, located slightly proximal to the proximal extent of the lateral edge of sulcus patellaris: absent (0); present (1). New Character.

355. Patella, ossified patella: absent (0); present (1). Livezey and Zusi (2007a:2051).

356. Patella, association with ligamentum patellae or tendo patellae ossificans: absent, essentially independent or only weakly constrained (0); present, associated with robust ligamentum patellae (1); present, co-ossified with robust tendo patellae ossificans (2). Livezey and Zusi (2007a:2053).

357. Patella, morphology of cranial face: nondescript or weakly demarcated (0); bilobate, distinct tumulus or jugum (1); cristate, marked dorsoventrally oriented crista partitioning approximately equal lateral and medial depressions (2); cuneate, prominent, elongate, broadly based eminentia aligned with major axis of tibiotarsus (3). Livezey and Zusi (2007a:2054).

358. Patella, marked sulcus for tendon of musculus ambiens: absent (0); present (1). Mayr (2005a:43); Livezey and Zusi (2007:2055).

359. Tibiotarsus, relative development of intercondylar tubercle on proximal articular facet of tibiotarsus: low and weakly developed (0), proximally high, mound-like and robust (1). Livezey and Zusi (2007a:2059); Warheit (1990:TTR3).

360. Tibiotarsus, relative proximal elongation of cristae cnemiales cranialis: low to slightly extended proximally (0); greatly enlarged and extending far proximally (1). Mayr (2005a:44); Livezey and Zusi (2007a:2071).

361. Tibiotarsus, proximodistal extent of cranial ridge of crista cnemialis cranialis: moderate, distal end of ridge is proximal or slightly distal to proximal end of crista fibularis (0); extensive, ridge extends distally across tibiotarsus well distal to midpoint of crista fibularis (1). Modified from Livezey and Zusi (2007a:2085).

362. Tibiotarsus, development of cranial cnemial crest: crest prominent and well-developed (0); crest rudimentary, reduced to rounded truncated jugum (1). Livezey and Zusi (2007a:2088, 2098).

363. Tibiotarsus, orientation of the distal edge of the cranial cnemial crest: extends straight, or nearly straight distally (0); extends distally with a distinct medial inflection, such that the distal end of the crest contacts impressio ligamentum collateralis medialis (1). Warheit (1990:TTR1).

364. Tibiotarsus, relative development of tuberosity for insertion of m. femorotibialis internus, pars distalis on the medial side of the tibiotarsus, at a level near the distal terminus of crista cnemialis cranialis and the proximal terminus of crista fibularis: tuberosity absent or small mound-shaped tubercle (0); tuberosity well-developed and proximodistally elongate (1). Livezey and Zusi (2007a:2092).

365. Tibiotarsus, distinct distal notch in craniodistal tip of cranial cnemial crest, resulting in a hook-like appearance in medial aspect: absent (0); present (1). Warheit (1990:TTR10).

366. Tibiotarsus, relative curvature of cranial edge of crista cnemialis cranialis: fairly linear or convex (0); strongly convex and extended cranially at midpoint, giving the edge a triangular appearance in medial aspect (1). New Character.

367. Dorsal margin of the crista cnemialis lateralis in anterior aspect: essentially concave throughout (0); sigmoidal, convex laterally and concave medially (1); linear and variably sloping throughout (2); essentially convex throughout (3). Livezey and Zusi (2007a:2078).

368. Tibiotarsus, margin of crista cnemialis lateralis oriented cranially, such that the cranial edges of crista cnemialis lateralis and cranialis are nearly parallel in proximal aspect: absent (0); present (1). Livezey and Zusi (2007a:2097).

369. Well-developed triangular fossa on lateral face of base of crista cnemialis lateralis: absent (0); present (1). Livezey and Zusi (2007a:2104).

370. Tibiotarsus, relative development of facet on crista cnemialis lateralis for origin of m. fibularus longus: moderately broadened (0); extremely robust and broadened into a large facet (1). Livezey and Zusi (2007a:2107).

371. Tibiotarsus, distinct U-shaped notch between crista patellaris and proximalmost portion of crista cnemialis lateralis: absent (0); present (1). New Character.

372. Tibiotarsus, development of plantaris fossa on caudomedial border of proximal tibiotarsus, just distal to rim of medial articular facies: absent or weakly excavated (0); deep, well-excavated fossa (1). Siegel-Causey (1988:131).

373. Tibiotarsus, pneumaticity associated with fossa flexoria on caudal surface of proximal portion of tibiotarsus: absent (0); present (1). Livezey and Zusi (2007a:2115).

374. Tibiotarsus, proximodistal length of foramen interosseum distale relative to foramen interosseum proximale: subequal or foramen interosseum distale slightly longer (0); foramen interosseum distale significantly longer (1); foramen interosseum distale significantly shorter, essentially occluded by its proximity to tibiotarsus (2). Livezey and Zusi (2007a:2129, 2130).

375. Tibiotarsus, proximal half of craniomedial border of tibiotarsus raised in a sharp medial ridge: absent (0); present (1). New Character.

376. Tibiotarsus, morphology of tuberosity for attachment of proximomedial portion of retinaculum mm. extensorum: proximodistally elongate, raised crest (0); oval to circular scarred impression (1); tuberosity absent (2). Livezey and Zusi (2007a:2133).

377. Tibiotarsus, morphology of distolateral attachment site for retinaculi mm. extensorum: relatively indistinct scar or raised tuberculum (0); deep, circular pit (1). Livezey and Zusi (2007a:2136).

378. Tibiotarsus, relative mediolateral position of distal portion of sulcus extensoris on cranial face of distal tibiotarsus: sulcus aligned in middle of cranial face (0); sulcus distinctly lateral (1); sulcus distinctly medial (2). Livezey and Zusi (2007a:2135).

379. Tibiotarsus, tuberculum ligamenti tibiometatarsale intercondylare (sensu Livezey and Zusi, 2007a:2137): absent or indiscernible (0); present (1). Livezey and Zusi (2007a:2137).

380. Tibiotarsus, relative development of tuberculum retinaculi m. fibularis: absent or faint ridge (0); present as laterally prominent tuberculum or scarred ridge (1). Livezey and Zusi (2007a:2138).

381. Tibiotarsus, relative distal extent of condyles: subequal (0); distal end bent medially, and condylus medialis protruding slightly further distally than condylus lateralis (1); condylus medialis protruding significantly further distally, giving the edge of the distal tibiotarsus a near “L”-shaped outline in cranial or caudal aspect (2). Mayr (2005a:45); Livezey and Zusi (2007a:2145).

382. Tibiotarsus, lateral rotation of distal tibiotarsus and condyles relative to proximal tibiotarsus: absent (0); present (1). Livzey and Zusi (2007a:2149).

383. Tibiotarsus, orientation of cranial half of lateral condyle: craniocaudally directed, in the same plane as caudal half (0); distinct lateral bend, craniolateral-caudally oriented (1). Warheit (1990:TTR4).

384. Tibiotarsus, morphology of rim of medial condyle of tibiotarsus: circular to suborbiculate (0); subrectangular and significantly elongate craniocaudally at its distal end, with rounded craniodistal and caudodistal vertices (1). Livezey and Zusi (2007a:2151).

385. Tibiotarsus, relative development of medial epicondylar depression: absent or weak (0); deep, well-defined sulcus, particularly caudodistal to medial epicondyle (1). Livezey and Zusi (2007a:2154).

386. Tibiotarsus, distinct notch in middle of distal rim of medial condyle: absent (0); present (1). Mayr (2005a:56). See also Livezey and Zusi (2007a:2155).

387. Tibiotarsus, weak, raised ridge extending across craniocaudal midline of incisura intercondylaris: absent (0); present (1). Livezey and Zusi (2007a:2162).

388. Tibiotarsus, relative mediolateral position of impressio ligamenti intercondylaris on the cranial face of incisura interconydlaris: central, essentially encompassed within area intercondylaris (0); significantly medial, resulting in a distinct excavation in the lateral side of the medial condyle (1). Livezey and Zusi (2007a:2168).

389. Tibiotarsus, relative development of sulcus m. fibularis brevis: indistinct or very weakly developed (0); sulcus distinct and moderately developed (1). Livezey and Zusi (2007a:2169).

390. Tibiotarsus, relative development of trochlea cartilaginis tibialis: margins and sulcus moderately developed (0); margins well-developed and sulcus deep (1). Livezey and Zusi (2007a:2153, 2170, 2173).

391. Tibiotarsus, symmetry of medial and lateral margins of proximal end of trochlea cartilaginis tibialis: relatively symmetrical (0); markedly asymmetric at proximal end, with distinct lateral kink, or displacement, of proximal end of medial margin (1). Livezey and Zusi (2007a:2172).

392. Tibiotarsus, lateral crest of trochlea cartilaginis tibialis extends distinctly greater caudally than medial crest: absent (0); present (1). Livezey and Zusi (2007a:2174).

393. Tibiotarsus, abrupt transverse disjunct between proximal border of trochlea cartiliaginis tibialis and caudal border of distal tibiotarsus shaft: absent (0); present (1). New Character, though see also Warheit (1990:TTR7).

394. Tibiotarsus, medial ridge of trochlea cartilaginis tibialis hypertrophied, robust, and mound-like: absent (0); present (1). New Character, though see also Livezey and Zusi (2007a:2170).

395. Tibiotarsus, orientation of canalis extensorius: proximodistal, with weak oblique distomedial departure (0); distinctly oblique proximolateral to distomedial orientation (1). Livezey and Zusi (2007a:2178, 2181).

396. Tibiotarsus, angle of pons supratendineus relative to transverse plane of tibiotarsus: low, between 0º and 45º (0); extremely high, greater than 45º (1). Warheit (1990:TTR8).

397. Fibula, relative concavity of proximal articular surface: convex (0); sharply linear (1); distinctly concave (2). Livezey and Zusi (2007a:2186).

398. Fibula, distal extent relative to tibiotarsus: fibular spine extends at least one-half, but less than three-fourths of tibiotarsus (0); fibular spine extends at least three-fourths, but not past proximal tarsals of tibiotarsus (1). Livezey and Zusi (2007a:2191).

399. Fibula, marked caudal offset of fibular shaft relative to long axis of tibiotarsus proximal to m. iliofibularis tubercule, in some cases resulting in a narrow, proximodistally elongate fissuriform foramen between tibiotarsus and fibula in lateral aspect: absent (0); present (1). Livezey and Zusi (2007a:2195).

400. Fibula, deeply concave, proximodistally elongate-oval to upside-down triangular fossa on lateral surface of proximal fibula, just below fibular head: absent (0); present (1). New Character.

401. Fibula, distinct caudodistally trending sulcus on craniolateral border of fibula: absent (0); present (1). New Character.

402. Fibula, strong, distally convex “hood” formed by overhanging caudal edge of fibular caput: absent (0); present (1). New Character.

403. Fibula, caudal border of caput fibula in lateral aspect: squared-off or gently rounded (0); slightly caudally extended as a sharp, triangular point (1). New Character.

404. Tarsometatarsus, proximodistal length of tarsometatarsus relative to the femur: moderate, tarsometatarsus subequal or slightly longer or shorter than femur (0) short, tarsometatarsus less than 1/2 of the length of the femur (1); long, tarsometatarsus distinctly longer than femur (2). Mayr (2005a:46); (Livezey and Zusi, 2007a:1963).

405. Tarsometatarsus, development of metatarsal I and first digit: metatarsal I robust, retaining proximal and ungual phalanges (0); metatarsal I vestigial, with vestigial or absent phalanx (1). Livezey and Zusi (2007a:2221).

406. Tarsometatarsus, development of extensor pits on metatarsal I: absent or weak (0): well-developed (1). Livezey and Zusi (2007a:2229).

407. Tarsometatarsus, articulation of metatarsal I with metatarsal II: ligamentous articulation (0); metatarsal I ossified to metatarsal II (1). Livezey and Zusi (2007a:2445).

408. Tarsometatarsus, development and orientation of eminentia intercondylaris (= “intercotylar prominence”): proximally high and well-developed (0); short, with a distinct spherical proximodorsal projection (1); short, and rounded, weakly developed with no dorsal component (2). Warheit (1990:TMT2).

409. Tarsometatarsus, relative mediolateral position of eminentia intercondylaris on tarsometatarsus: at or near midline of tarsometatarsus (0); distinctly lateral to midline of tarsometatarsus (1). New Character.

410. Tarsometatarsus, relative proximodistal position of tubercle for insertion of m. tibialis cranialis on the dorsal face of metatarsals II and III: proximal, near the end of the tarsometatarsus (0); at or just proximal of the midpoint of the tarsometatarsus (1). Livezey and Zusi (2007a:2236); Warheit (1990:TMT10).

411. Tarsometatarsus, well-developed, proximally elevated medial rim to medial cotyle, tapering proximally to a weak point: absent (0); present (1). Livezey and Zusi (2007a:2249).

412. Tarsometatarsus, relative proximal extents of cotyles: subequal (0); medial cotyle proximal to lateral cotyle (1); medial coytle distal to lateral cotyle (2). Livezey and Zusi (2007a:2250; see also 2248).

413. Tarsometatarsus, relative sizes of articular facets of proximal cotyles: subequal (0); medial cotyle distinctly more expansive than lateral cotyle (1). Livezey and Zusi (2007a:2251); Warheit (1990:TMT3).

414. Tarsometatarsus, relative development of dorsal rim of lateral cotyle: rim present (0); rim extremely reduced or absent (1). Livezey and Zusi (2007a:2253); Warheit (1990:TMT3).

415. Tarsometatarsus, relative development of tuberositas ligamentum collateralis lateralis: indistinct to weakly developed tuberosity or fovea (0); substantially developed, rounded tubercle on the dorsolateral border of the proximal tarsometatarsus, just below the cotylar rim (1). Livezey and Zusi (2007a:2255).

416. Tarsometatarsus, size of foramina/fenestrae intertarsometatarsalia lateralis and medialis on the dorsal face of the proximal end of the tarsometatarsus: minute or fairly small (0); very large (1). Livezey and Zusi (2007a:2293).

417. Tarsometatarsus, extensive pneumaticity associated with fossa infracotylaris dorsalis on proximal tarsometatarsus: absent (0); present (1). Modified from Livezey and Zusi (2007a:2265).

418. Tarsometatarsus, broad depression on proximal tarsometatarsus (= “fossa infracotylaris plantaris”) between cotyles and hypotarsus: absent (0); present (1). Livezey and Zusi (2007a:2267).

419. Tarsometatarsus, shape of crista medialis hypotarsi: elongate, typically twice as long proximodistally as wide mediolaterally (0); wide, typically as wide mediolaterally as long proximodistally or only slightly less so (1). New Character (though see also Livezey and Zusi, 2007a:2271).

420. Tarsometatarsus, plantar extent of crista lateralis hypotarsi relative to adjacent surfaces of hypotarsus: weak, crista is absent or does not extend far plantar (0); moderate to strong, crista typically extends as far plantar as crista medialis lateralis hypotarsi (1). Livezey and Zusi (2007a:2272).

421. Tarsometatarsus, plantar length of crista medialis hypotarsi relative to dorsoplantar width of proximal articular surface of tarsometatarsus: weak to moderate, crista is at most subequal to dorsoplantar width of proximal tarsometatarsus (0); substantial, crista is significantly longer than dorsoplantar width of proximal tarsometatarsus (1). Livezey and Zusi (2007a:2276).

422. Tarsometatarsus, crista medialis hypotarsi bifurcate at plantar end: no (0); yes (1). New Character.

423. Tarsometatarsus, development of “tendinal passage 1” of Strauch (1978), or “anteromedial canal” of Stallcup (1954:Fig. 6), or canalis tendinis insertii m. flexor digitorum longus (Vanden Berge and Storer, 1995:Fig. 8): absent, indiscernible, or shallow sulcus (0); present, deep, majority-delimited sulcus or bony canal (1). Livezey and Zusi (2007a:2278). See also Mayr (2005a:47).

424. Tarsometatarsus, pneumaticity of fossa parahypotarsalis medialis around and proximal to medial plantar proximal vascular foramina: absent (0); present (1). New Character.

425. Tarsometatarsus, pneumatic foramen in fossa parahypotarsalis lateralis, proximal and lateral to lateral plantar proximal vascular foramina, and corresponding pneumatic depression on proximal tarsometatarsus, between lateral cotyle and lateral calcaneal ridge: absent (0); present (1). New Character.

426. Tarsometatarsus, development of “tendinal passage 3” of Strauch (1978), or “anterolateral canal” of Stallcup (1954:Fig.6), or canalis tendinis insertii m. flexor hallucis longus of Vanden Berge and Storer (1995:Fig. 8): absent, indiscernible, or shallow sulcus (0); present, deep, majority-delimited sulcus or bony canal (1). Livezey and Zusi (2007a:2280).

427. Tarsometatarsus, mediolateral position of crista medialis hypotarsi relative to medial proximal cotyle of tarsometatarsus: crista located at midline or slightly lateral to midline through medial cotyle (0); crista located medial to midline of medial cotyle (1). New Character.

428. Tarsometatarsus, marked mediolateral compression of tarsometatarsal shaft such that dorsoplantar depth exceeds mediolateral width: absent (0); present (1). Livezey and Zusi (2007a:2287).

429. Tarsometatarsus, shape of medial margin of tarsometatarsal shaft at its proximal extent: rounded, similarly conformed as rest of shaft (0); compressed dorsoplantarly, relatively thin and/or crest-like (1). Livezey and Zusi (2007a:2288).

430. Tarsometatarsus, concavity of lateral margin of distal tarsometatarsal shaft in dorsal perspective: concave, distally curving smoothly to lateral face of trochlea of digit IV, resulting in symmetrical (or nearly so) medial and lateral borders of distal tarsometatarsal shaft (0); sublinear, trochlea IV splays laterally only slightly, resulting in asymmetry with medial border (1); linear, trochlea IV extends almost straight distally at the distal end of the tarsometatarsus (2). Livezey and Zusi (2007a:2289); Warheit (1990:TMT15).

431. Tarsometatarsus, well-developed scar for the origin of m. extensor hallucis longus on dorsomedial border of tarsometatarsal shaft, extending obliquely distoplantad from fossa infracotylaris dorsalis toward articulation with metatarsal I: absent or indistinct (0); present and prominent (1). Livezey and Zusi (2007a:2291).

432. Tarsometatarsus, relative dorsal prominence of medial and lateral margins of tarsometatarsal shaft, and relative torsion of shaft: margins essentially equally prominent, or proximal end of lateral margin slightly more prominent dorsally, but shaft lacking torsion (0); lateral margin distinctly more prominent throughout most of tarsometatarsal shaft length, and distinct medial torsion of distal half of metatarsal shaft (1). Livezey and Zusi (2007a:2292).

433. Tarsometatarsus, degree of development of area of origin for m. abductor digiti IV, and also passage/sulcus for joint tendon of m. fibularis longi and m. flexor perforans et perforatus digiti III on the lateral half of the plantar face of the tarsometatarsal shaft: weakly defined, typically only discernable proximally (0); well-excavated and distinct sulcus, distally extensive (1). Livezey and Zusi (2007a:2295, 2303).

434. Tarsometatarsus, relative development of scar/fossa for metatarsal I on plantar/medial side of distal tarsometatarsus: weak or hardly discernable (0); strong circular scar or elliptical fossa (1). Livezey and Zusi (2007a:2312).

435. Tarsometatarsus, relative dorsoplantar position of fossa metatarsi I on tarsometatarsal shaft: plantar, with some minor medial component (0); primarily medial (1). Livezey and Zusi (2007a:2314).

436. Tarsometatarsus, foramen vasculare distale: present with openings between metatarsals III and IV on both the dorsal and plantar surfaces of the tarsometatarsus (0); present with opening between metatarsals III and IV on the dorsal surface between trochlea III and IV only (1); proximal portion of foramen present, but completely open distally (2); foramen completely absent (3). Livezey and Zusi (2007a:2315); Ksepka et al. (2006:163); Mayr (2005a:48).

437. Tarsometatarsus, relative development of distal end of sulcus extensorius in area of foramen vasculare distale: sulcus present but relatively shallow (0); suclus extremely deep (1). Warheit (1990:TMT5).

438. Tarsometatarsus, canalis/sulcus interosseus tendineus distalis: foramen continued distally by completely enclosed canalis tendineus distalis (0); foramen continued distally by dorsoplantarly exposed sulcus (1). Livezey and Zusi (2007a:2325).

439. Tarsometatarsus, relative development of sulcus on dorsal face of metatarsal II trochlea: extremely weak or absent (0); present and distinct, continuous across articular face (1). Livezey and Zusi (2007a:2339).

440. Tarsometatarsus, concave incisure in the medial side of the distal edge of trochlea metatarsal II: absent (0); present (1). Livezey and Zusi (2007a:2351).

441. Tarsometatarsus, medioplantar eminence on trochlea metatarsal II: absent or very weakly developed (0); present, asymmetrically developed relative to lateral side of trochlea, projecting plantarly and variably medially (1). Livezey and Zusi (2007a:2352).

442. Tarsometatarsus, concave incisure in the lateral side of the distal edge of trochlea metatarsal IV: absent (0); present (1). New Character (though see 475 above, and Livezey and Zusi, 2007a:2351).

443. Tarsometatarsus, lateral edge of basis trochleae on plantar side of metatarsal II extends slightly further proximally than medial edge, narrowing to a triangle shape: absent (0); present (1). Livezey and Zusi (2007a:2357).

444. Tarsometatarsus, distinct depression on proximal end of dorsal surface of trochlea metatarsal III: absent (0); present (1). Warheit (1990:TMT7).

445. Tarsometatarsus, relative distal extents of trochleae metatarsals: II < III > IV, and II subequal to IV (0); II < III > IV, and II much less than IV (1); II < III > IV, and II > IV (2); II > III > IV (3). Livezey and Zusi (2007a:2361); Mayr (2005a:49); Warheit (1990:TMT9).

446. Pes, relative lengths of digits III and IV: digit III longer than digit IV (0); digit IV slightly longer than digit III (1); digit IV significantly longer than digit III, often by nearly the entire distal phalanx of digit IV (2). Livezey and Zusi (2007a:2371).

447. Pes, number of phalanges on digit I: two (0); one (1); zero (2). Livezey and Zusi (2007a:2375; see also 2221); Mayr (2005a:58).

448. Pes, degree of curvature of plantar face of proximal phalanx of digit I relative to proximal phalanges II-IV: unmarked or weak (0); distinct concave curvature (1). Livezey and Zusi (2007a:2381).

449. Pes, extremely robust, semicircular tab-shaped flange projecting from medial border of proximal end of phalanx I-1: absent (0); present (1). New Character.

450. Pes, proximal articular surface of phalanx I-1 (when present): nearly flat or weak cotyle (0); strongly convex trochlea (1). New Character.

451. Pes, development of tubercle for insertion of m. flexor perforatus digiti II on medial face of proximal end of digit II-1: relatively unmarked or weakly developed (0); robust and expanded medioplantarly into a strong flange (1). Livezey and Zusi (2007a:2391).

452. Pes, shape of proximal articular facet of II-1: relatively unmarked, or weakly excavated and bowl-shaped (0); lateral half of cotyle higher proximally than medial half, with block-like process at plantar border, and medial half of cotyle excavated as a plantarly sloping fossa (1). New Character.

453. Pes, pneumaticity associated with flexor fossa of II-1: absent (0); present (1). New Character.

454. Pes, relative curvature of shaft of phalanx II-1: straight (0); significantly bowed, convex medially and concave laterally (1). New Character.

455. Pes, relative development of dorsal intercotylar process of III-1: absent or weakly expanded proximally (0); well-developed into strong triangular proximal protuberance (1). New Character.

456. Pes, two paired proximiodistally elongate low ridges present on the dorsal surface of phalanx III-1: absent (0); present (1). New Character.

457. Pes, development of tubercle for insertion of m. extensor brevis digiti III on medial face of proximal end of digit III-1: relatively unmarked or weakly developed (0); robust and expanded medioplantarly into a strong flange (1). Livezey and Zusi (2007a:2396).

458. Pes, proximodistal length of IV-1 relative to III-1: IV-1 distinctly shorter than III-1 (0); IV-1 subequal in length to III-1 (1); IV-1 distinctly longer than III-1 (2). New Character.

459. Pes, development of flexor tubercles on plantar side of proximal portion of IV-1: weakly developed, to proximally restricted knob-like tubercles (0); symmetrical ridges extending prominently proximodistally (1). Livezey and Zusi (2007a:2404).

460. Pes, relative development of distal condyles of IV-1: medial condyle subequal or slightly more robust than lateral condyle (0); medial condyle significantly more robust than lateral condyle (1). New Character.

461. Pes, proximodistal location of flexor tubercles on unguals of digits II-IV: proximal, at or near the proximal margin of the ungual (0); well distal to proximal margin, at or near midpoint of plantar face of ungual (1). Livezey and Zusi (2007a:2406).

462. Pes, neurovascular sulcus on dorsolateral face of unguals II-IV: absent (0); present (1). Livezey and Zusi (2007a:2429).

463. Pes, relative development of medial and lateral grooves on unguals (particularly ungual III): absent or weak (0); grooves strongly excavated into ungual (1). New Character.

464. Pes, strong dorsoventral compression of phalanges of pes: absent (0); present (1). New Character.

**Additional References**

Warheit KI, Good DA, de Queiroz K (1989) Variation in numbers of scleral ossicles and their phylogenetic transformations within the Pelecaniformes. Auk 106: 383-388.
