# Supplementary material for: Phylogenetic Analysis of Pelecaniformes (Aves) Based on Osteological Data: Implications for Waterbird Phylogeny and Fossil Calibration Studies
Source: PLoS One. 2010 Oct 14;5(10):e13354. doi: 10.1371/journal.pone.0013354 (PMC2954798; doi:10.1371/journal.pone.0013354)
Supplement: Appendix S3 — Morphological data matrix. (0.12 MB DOC) [file pone.0013354.s003.doc]

# Appendix S3: Morphological data matrix

Taxon by character data matrix including codings for 59 taxa across 464 morphological characters. Polymorphic entries and/or uncertainties are given as lettered codes: A = [0,1], B = [1,2], C = [0,2], D = [1,3], inapplicable data are given as “–“, and missing data are given as “?”.

_______________________________________________________________________

Character 4

## Taxa 0_

*Eudromia elegans*  000001-0000000000000000000000000010?0000

*Gallus gallus* 000000-000000010000000000000001000000000

*Gavia immer* 00-000-000000010000000000000000000000001

*Podiceps auritus* 00-000-000000011000000000000001011000000

*Pygoscelis antarctica* 1000010010000000000000000100000000000001

*Eudyptula minor* 1000010010010000000000000100000000000001

*Phoebastria nigripes* 2000010010011000110000011000001000000001

*Puffinus gravis* 2000010010011000110000011000001000000001

*Pelecanoides urinatrix* 2000010011011000000000011000000000000001

*Oceanodroma castro* 2000010011011000000000011000001000000001

*Oceanites oceanicus* 2000010011011000000000011000001000000001

*Lithoptila abdounensis* ????????0?????00????????????10?????????0

*Prophaethon shrubsolei* 0?0100-0001???0?0?00001?????1?????0??0?0

*Phaethon rubricauda* 0101000000101000000200000001101000000010

*Phaethon lepturus* 0101000000101000000200000001101000000010

*Phaethon aethereus* 0101000000101000000200000001101000000010

*Phoenicopterus chilensis* -001000000001010000000000000000110000000

*Ciconia abdimii* 0001000000001010100000000000000011000000

*Cochlearius cochlearius* 0001010000001010000010000000100110010100

*Ardea herodias* 0001010000001000000000000000000110010100

*Eudocimus albus* -0-1010000001000000010000000000110001000

*Platalea ajaja* 0001010000001000000010000000000110001000

*Scopus umbretta* 2101011000001010100010000010001010000000

*Balaeniceps rex* 1101010100001010100010000011101000000010

*Pelecanus erythrorhnychos* 1101110100101010100010100001001001000010

*Limnofregata* 2?01?1-0?0101?0?0??0????????01??000001?0

*Fregata minor* 2101011100101010000010000001011000020100

*Fregata ariel* 2101011100101010000010000001011000020100

*Fregata magnificens* 2101011100101010000010000001011000020100

*Papasula abbotti* 100101?20010??1?000011100011001000100100

*Sula sula* 1001110200101010000011100011001000100100

*Sula leucogaster* 1001110200101010000011100011001000100100

*Sula dactylatra* 1001110200101010000011100011001000100100

*Sula variegata* 1001110200101010000011100011001000100100

*Sula nebouxii* 1001110200101010000011100011001000100100

*Morus serrator* 1011010200101010001011100011001000100100

*Morus bassanus* 1011010200101010001011100011001000100100

*Morus_capensis* 1011010200101010001011100011001000100100

*Phocavis maritimus* ????????????????????????????????????????

*Plotopterum joaquinensis* ????????????????????????????????????????

*Copepteryx hexeris* ????????????????????????????????????????

*Tonsala hildegardae* ????????????????????????????????????????

*Anhinga anhinga* 0001010200100000001011100001200100100100

*Anhinga rufa* 0001010200100000001011100001200100100100

*Borvocarbo stoeffelensis* ????????????????????????????????????????

*Phalacrocorax africanus* 2001010200100000001011100001001000100100

## *Phalacrocorax pygmaeus* 2001010200100000001011100001001000100100

*Phalacrocorax melanoleucos* 2001010200100000001011100001001000100100

*Phalacrocorax auritus* 2001010200100000001011100001001000100100

*Phalacrocorax brasilianus* 2001010200100000001011100001001000100100

*Phalacrocorax gaimardi* 2001010200100001001011100001001000100100

*Phalacrocorax carbo* 2001010200100000001111100001001000100100

*Phalacrocorax penicillatus* 2001010200100001001011100001000000100100

*Phalacrocorax pelagicus* 2001011200100001001011100001000000100100

*Phalacrocorax aristotelis* 2001011200100101001011100001001000100100

_______________________________________________________________________

Character 4

## Taxa 0_

*Phalacrocorax bougainvilli* 2001011200100001001011100001001000100100

*Phalacrocorax magellanicus* 2001011200100001001011100001001000100100

*Phalacrocorax atriceps* 2001011200100001001011100001001000100100

## *Phalacrocorax albiventer* 2001011200100101001011100001001000100100_

_______________________________________________________________________

Character 8

### Taxa 0_

*Eudromia elegans* 0000-000000000000000000000000-0000000000

*Gallus gallus* 0000-000000010000000000000000-0000000000

*Gavia immer* 0000-000001010000000010001000-0000000001

*Podiceps auritus* 0000-000001000000000010000000-0000000001

*Pygoscelis antarctica* 0000-000001000000000010100100-0000000101

*Eudyptula minor* 0000-000001000000000010100100-0000000101

*Phoebastria nigripes* 0000-000000000011000010000100-0001000001

*Puffinus gravis* 0000-000001000111000000000100-0001010101

*Pelecanoides urinatrix* 0000-000001000101000000000000-0001010101

*Oceanodroma castro* 0000-000000000001000000000000-0001000101

*Oceanites oceanicus* 0000-000000000001000000000000-0001000101

*Lithoptila abdounensis* 0011?0000100010110010?01?0110-?100000001

*Prophaethon shrubsolei* 0110?0000100010110100101?0110-0110000001

*Phaethon rubricauda* 01000000010001011010010100110-0100000001

*Phaethon lepturus* 01000000010001011010010100110-0100000001

*Phaethon aethereus* 01000000010001011010010100110-0100000001

*Phoenicopterus chilensis* 0000-000000010000000110100000-0000000001

*Ciconia abdimii* 0000-000000110000000010000000-0000000001

*Cochlearius cochlearius* 0000-100000110000000011000000-0000010002

*Ardea herodias* 0000-100000110000000011000000-0000010002

*Eudocimus albus* 0000-000000110100000110000000-0000000001

*Platalea ajaja* 0000-000000110100000110000000-0000000001

*Scopus umbretta* 0000-000000110000001010000000-0000000001

*Balaeniceps rex* 0000-000000000000000010000000-0000000001

*Pelecanus erythrorhnychos* 00010000000000000001010000000-0010000002

*Limnofregata* 00???000?00??????0????000010??????00???2

*Fregata minor* 00010000000000000000010000100-0000000002

*Fregata ariel* 00010000000000000000010000100-0000000002

*Fregata magnificens* 00010000000000000000010000100-0000000002

*Papasula abbotti* 1001?000000?00000101011111100-1010000002

*Sula sula* 10010011000000000001011111100-1010011002

*Sula leucogaster* 10010011100000000A01011111100-1010011002

*Sula dactylatra* 10010011100000000001011111100-1010011002

*Sula variegata* 10010011100000000001011111100-1010011002

*Sula nebouxii* 10010011100000000001011111100-1010011002

*Morus serrator* 10010012000000000101011111100-1010011002

*Morus bassanus* 10010012000000000101011111100-1010011002

*Morus_capensis* 10010012000000000101011111100-1010011002

*Phocavis maritimus* ????????????????????????????????????????

*Plotopterum joaquinensis* ????????????????????????????????????????

*Copepteryx hexeris* ????????????????????????????????????????

*Tonsala hildegardae* ????????????????????????????????????????

*Anhinga anhinga* 10010010000100000001011101100-1010000012

*Anhinga rufa* 10010010000100000001011101100-1010000012

*Borvocarbo stoeffelensis* ?????0?????????????????????????????1????

*Phalacrocorax africanus* 20010010000000000001011101100-1010010002

*Phalacrocorax pygmaeus* 2001001000000000000101110110??1010010002_

_______________________________________________________________________

Character 8

## Taxa 0_

*Phalacrocorax melanoleucos* 2001001000000000000101110110??1010010002

*Phalacrocorax auritus* 2001001000000000000101110110101010010002

*Phalacrocorax brasilianus* 2001001000000000000101110110101010010002

*Phalacrocorax gaimardi* 2001001000000000000101110110??1010000002

*Phalacrocorax carbo* 20010010000000000001011101100-1010010002

*Phalacrocorax penicillatus* 20010010000000000001011101100-1010110002

*Phalacrocorax pelagicus* 20010010000000000001011101100-1010000002

*Phalacrocorax aristotelis* 2001001000000000000101110110101010010002

*Phalacrocorax bougainvilli* 2001001000000000000101110110111010010002

*Phalacrocorax magellanicus* 2001101000000000000101110110??1010110002

*Phalacrocorax atriceps* 2001101000000000000101110110??1010000002

## *Phalacrocorax albiventer* 2001101000000000000101110110111010000002_

_______________________________________________________________________

Character 1

2

## Taxa 0_

*Eudromia elegans* -000-00000000000000010000000000201000000

*Gallus gallus* -000-00000001000110021000000000000000020

*Gavia immer* -001000000011000110000000100000003000000

*Podiceps auritus* -001001000000000010020000100000003000010

*Pygoscelis antarctica* -001-00000010000110110001110000001020000

*Eudyptula minor* -001-000000100001101100011100000010200?0

*Phoebastria nigripes* -000-00001000000100011000000010212001001

*Puffinus gravis* -000-00001000000100011000000010012000001

*Pelecanoides urinatrix* -001-00001000000110011000100000012020100

*Oceanodroma castro* -000-00001000000110011000100000012000100

*Oceanites oceanicus* -000-00001000000110010000100000011000100

*Lithoptila abdounensis* 001000?0????????????????????????????????

*Prophaethon shrubsolei* 001000?000??0???????????0?0?????????????

*Phaethon rubricauda* -010000000000000000111000000000202000001

*Phaethon lepturus* -010000000000000000111000000000202000001

*Phaethon aethereus* -010000000000000000111000000000202000001

*Phoenicopterus chilensis* -000-00000201000000020000000010202000000

*Ciconia abdimii* 0000-00000000000000021000000011201000001

*Cochlearius cochlearius* 0010-00000000010000020110000000200000000

*Ardea herodias* 0010-00000000010000020110000010200000000

*Eudocimus albus* -000?00000001000000021110000000202000000

*Platalea ajaja* -000-00000001000000021110000000201000000

*Scopus umbretta* -000-00000000000000010000000010201000001

*Balaeniceps rex* -000-0000100001010012010000001120100A011

*Pelecanus erythrorhnychos* 001000000100000010012010?000110201001001

*Limnofregata* ?????0?0?1??0?10????110?0?010???0200?01?

*Fregata minor* 0010000001000110101011000001010202000110

*Fregata ariel* 0010000001000110101011000001010202000110

*Fregata magnificens* 0010000001000110101011000001010202000110

*Papasula abbotti* 0010001011??0011100120100000110101010011

*Sula sula* 0010002011000011100120100000110101010011

*Sula leucogaster* 001000201100001110012010000011010B010011

*Sula dactylatra* 0010002011000011100120100000110102010011

*Sula variegata* 0010002011000011100120100000110102010011

*Sula nebouxii* 0010002011000011100120100000110102010011

*Morus serrator* 0010001011000011100120100002110011010011

*Morus bassanus* 0010001011000011100120100002110011010011

*Morus_capensis* 0010001011000011100120100002110011010011_

_______________________________________________________________________

Character 1

2

## Taxa 0_

*Phocavis maritimus* ????????????????????????????????????????

*Plotopterum joaquinensis* ????????????????????????????????????????

*Copepteryx hexeris* ??????????????????????????1?????0B?2????

*Tonsala hildegardae* ??????????????????????????1?????????????

*Anhinga anhinga* 0110001101000001010120101100100000100110

*Anhinga rufa* 0110001101000001010120101100100000100110

*Borvocarbo stoeffelensis* ??????????1?0???????????????????????????

*Phalacrocorax africanus* 0010001101100111110120001100100000000011

*Phalacrocorax pygmaeus* 0010001101100111110120001100100000000011

*Phalacrocorax melanoleucos* 0010001101100111110120001100100000000011

*Phalacrocorax auritus* 0010001101100011110120001100100000000011

*Phalacrocorax brasilianus* 0010001101100011110120001100100000000011

*Phalacrocorax gaimardi* 0010001101100011110120001100100000000011

*Phalacrocorax carbo* 1010011101000011110120001100100000000011

*Phalacrocorax penicillatus* 1010111101000011110120001100100000000011

*Phalacrocorax pelagicus* 1010001101000011110120001100100000000011

*Phalacrocorax aristotelis* 0010111101000011110120001100100000000011

*Phalacrocorax bougainvilli* 0010101101000011110120001100100000000011

*Phalacrocorax magellanicus* 0010101101010011110120001100100000000011

*Phalacrocorax atriceps* 0010111101010011110120001100100000000011

## *Phalacrocorax albiventer* 0010101101010011110120001100100000000011_

_______________________________________________________________________

Character 1

6

## Taxa 0_

*Eudromia elegans* 00-0000000000000000100000000000000100000

*Gallus gallus* 00-010000000A000000000000000000000100000

*Gavia immer* 0100001000001100001010000010000000000000

*Podiceps auritus* 00-0001000000100000010000010000000000000

*Pygoscelis antarctica* 0100000010001100001110000010000000000010

*Eudyptula minor* 0100000010001100?00110000010000000000010

*Phoebastria nigripes* 0110001000010011010010000000011100000100

*Puffinus gravis* 1110001000100011010010000010011100000100

*Pelecanoides urinatrix* 1110000001001001010000000010011100000000

*Oceanodroma castro* 0110000001000000000000000010001000000000

*Oceanites oceanicus* 0100000000000000000000000010001000000000

*Lithoptila abdounensis* ????????????????????????????????????????

*Prophaethon shrubsolei* ????????????00??11????0?????????????????

*Phaethon rubricauda* 0101111000100100110000000001010100001100

*Phaethon lepturus* 0101111000100100110000000001010100001100

*Phaethon aethereus* 0101111000000100110000000001010100001100

*Phoenicopterus chilensis* 0110011000000000000110000010000000000000

*Ciconia abdimii* 0101011000002100010100001100000101001101

*Cochlearius cochlearius* 0101011000000000000000000000000000000000

*Ardea herodias* 0100011000000000000000000000000000000000

*Eudocimus albus* 0100011000102000000000000000000000000000

*Platalea ajaja* 0100011000102000000000000100000000001000

*Scopus umbretta* 0100001000002000000001101100000000000100

*Balaeniceps rex* 00-0002000010101020001100101000?01101100

*Pelecanus erythrorhnychos* 0101002000010111020101100101000101101101

*Limnofregata* 0?????1?001120?1010???1???0??101??00??0?

*Fregata minor* 0100001000012010020?01?10100111101000101

*Fregata ariel* 0100001000012010020?01?10100111101000101_

_______________________________________________________________________

Character 1

6

## Taxa 0_

*Fregata magnificens* 0100001000012010020?01?10100111101000101

*Papasula abbotti* 00-0001000011111010101100100110101101001

*Sula sula* 00-0001000011111010101100100110101101001

*Sula leucogaster* 00-0001000011111010101100100010101101001

*Sula dactylatra* 00-0001000011111010101100100010101101001

*Sula variegata* 00-0001000011111010101100100010101101001

*Sula nebouxii* 00-0001000011111010101100100010101101001

*Morus serrator* 0100001000011111010101100100010201101001

*Morus bassanus* 0100001000011111010101100100010201101001

*Morus_capensis* 0100001000011111010101100100010201101001

*Phocavis maritimus* ????????????????????????????????????????

*Plotopterum joaquinensis* ?????????????????1????1?????????????????

*Copepteryx hexeris* 0???????????11??01?10?10??000?01??11?01?

*Tonsala hildegardae* ?????????????????1????1???????????11001?

*Anhinga anhinga* 0100000100011111010101100100010111100001

*Anhinga rufa* 0100000100011111010101100100010111100001

*Borvocarbo stoeffelensis* ????????????????????????????????????????

*Phalacrocorax africanus* 0100001100011110010101100101010111100001

*Phalacrocorax pygmaeus* 0?00001100011110010101100101010111100001

*Phalacrocorax melanoleucos* 0?00001100011110010101100101010111100001

*Phalacrocorax auritus* 0100001100011110010101100101010111100001

*Phalacrocorax brasilianus* 0100001100011110010101100101010111100001

*Phalacrocorax gaimardi* 0100001100011110010101100101010111100001

*Phalacrocorax carbo* 0100001100011110010101100101010111100001

*Phalacrocorax penicillatus* 00-0001100011110010101100101010111100001

*Phalacrocorax pelagicus* 0100001100011110010101100101010111100001

*Phalacrocorax aristotelis* 0100001100011110010101100101010111100001

*Phalacrocorax bougainvilli* 00-0001100011110010101100101010111100001

*Phalacrocorax magellanicus* 0100001100011110010101100101010111100001

*Phalacrocorax atriceps* 0000001100011110010101100101010111100001

## *Phalacrocorax albiventer* 00-0001100011110010101100101010111100001_

_______________________________________________________________________

Character 2

0

## Taxa 0_

*Eudromia elegans* 0000000000000000-000000000000000-0000000

*Gallus gallus* 0000001000000000-000000000000000-0000000

*Gavia immer* 0000001000001010-000010010000000-0000001

*Podiceps auritus* 0000000000000000-000001010000000-0000001

*Pygoscelis antarctica* 0001102000001011-000011010000000-0000101

*Eudyptula minor* 00011020?0001011-000011010000000-000?001

*Phoebastria nigripes* 0010001001001010-000011110110000-0001000

*Puffinus gravis* 0000001001001010-000011101110000-0000001

*Pelecanoides urinatrix* 0000101000001010-000010101110000-0000011

*Oceanodroma castro* 0000001001001010-000010101010000-0000011

*Oceanites oceanicus* 0000001001001010-000010101010000-0000011

*Lithoptila abdounensis* ??????????0????0?00???????0100?0000???0?

*Prophaethon shrubsolei* ???0??????0????0?00??1?0??????0?????????

*Phaethon rubricauda* 0000100100001010-00001000001000000000001

*Phaethon lepturus* 0000100100001010-00001000001000000000001

*Phaethon aethereus* 0000100100001010-00001000001000000000001

*Phoenicopterus chilensis* 0000000000001010-000011010010000-0000001

*Ciconia abdimii* 0000000101011110-000000000100000-0000001_

_______________________________________________________________________

Character 2

0

## Taxa 0_

*Cochlearius cochlearius* 0100000000011110-000001000110000-0000001

*Ardea herodias* 0100000000011110-000000000100000-0000001

*Eudocimus albus* 0100000100011110-000010000000000-0000001

*Platalea ajaja* 0100000100011110-000010000000000-0000001

*Scopus umbretta* 01000001000010000000200000000100-0000001

*Balaeniceps rex* 0100000101001110000021000010000000000001

*Pelecanus erythrorhnychos* 0000000111011110000021000010010000000000

*Limnofregata* ???0??????0?0000?0???0?0??11?????????001

*Fregata minor* 0000000010001120-00020000011000000100001

*Fregata ariel* 0000000010001120-00020000011000000100001

*Fregata magnificens* 0000000010001120-00020000011000000100001

*Papasula abbotti* 0010000110011120000000000011010100010000

*Sula sula* 0010000110011120000100000011011100011001

*Sula leucogaster* 00100001100111200001A0000011011100011001

*Sula dactylatra* 0010000110011120000110000011010101010001

*Sula variegata* 0010000110011120000110000011010101010001

*Sula nebouxii* 0010000110011120000110000011010101010001

*Morus serrator* 0010000110011120100000000111000010010000

*Morus bassanus* 0010000110011120100000000111000010010000

*Morus_capensis* 0010000110011120100000000111000010010000

*Phocavis maritimus* ????????????????????????????????????????

*Plotopterum joaquinensis* ??????001000???101010???????????????????

*Copepteryx hexeris* ?001?0000110??B1010?00?000110?0???1??101

*Tonsala hildegardae* ???1100001101021010?0???0????????????101

*Anhinga anhinga* 1000010010010000001000000011100000001001

*Anhinga rufa* 1000010010010000001000000011100000001001

*Borvocarbo stoeffelensis* ??????????0?????????????????????????????

*Phalacrocorax africanus* 0000010010010000000000000011000000001001

*Phalacrocorax pygmaeus* 0000010010010000000000000011000000001001

*Phalacrocorax melanoleucos* 0000010010010000000000000011000000001001

*Phalacrocorax auritus* 0000010010010000000000000011000000001001

*Phalacrocorax brasilianus* 0000010010010000000000000011000000001001

*Phalacrocorax gaimardi* 0000010010010000000000000011000000001001

*Phalacrocorax carbo* 0000010010010000000000000011000000001001

*Phalacrocorax penicillatus* 0000010010010000000000000011000000000001

*Phalacrocorax pelagicus* 0000010010010000000000000011000000001001

*Phalacrocorax aristotelis* 0000010010010000000000000011000000001001

*Phalacrocorax bougainvilli* 0000010010010000000000000011000000001001

*Phalacrocorax magellanicus* 0000010010010000000000000011000000001001

*Phalacrocorax atriceps* 0000010010010000000000000011000000001001

## *Phalacrocorax albiventer* 0000010010010000000000000011000000001001_

_______________________________________________________________________

Character 2

4

## Taxa 0_

*Eudromia elegans* 0000000000000000000000000000000000000000

*Gallus gallus* 0000000000000000000000000000001000000000

*Gavia immer* 0000020000001000000002100000000000000000

*Podiceps auritus* 0000020010000000000002000000000000000000

*Pygoscelis antarctica* 00000200-2--200--001----000010100------0

*Eudyptula minor* 00000200-2-?200--101----0?0010100------0

*Phoebastria nigripes* 0001011011001000000000100011001100001010

*Puffinus gravis* 0000011011001010000000000011001100001010_

_______________________________________________________________________

Character 2

4

## Taxa 0_

*Pelecanoides urinatrix* 0000001011002000030000000011001000000010

*Oceanodroma castro* 000000101100201?00000?000011001000000010

*Oceanites oceanicus* 0000001011002000000000000011001000001010

*Lithoptila abdounensis* ???????????00?0??????020?0?0???1?0000010

*Prophaethon shrubsolei* ??????????????????0?????????????????????

*Phaethon rubricauda* 0010000010100100000000200100010000020000

*Phaethon lepturus* 0010000010100100000000200100010000020000

*Phaethon aethereus* 0010000010100100000000200100010000020000

*Phoenicopterus chilensis* 0000000010000100000000000000000000000000

*Ciconia abdimii* 0000000000000000000000000100010000000000

*Cochlearius cochlearius* 0000000000000000000000000000011000030000

*Ardea herodias* 0000000000000000000000000000011000030000

*Eudocimus albus* 0000000000000000000000000000010000000000

*Platalea ajaja* 0000000000000000000000000000010000000000

*Scopus umbretta* 0000000000000000000000000000010000000000

*Balaeniceps rex* 0000120000000100000000001000000000000000

*Pelecanus erythrorhnychos* 0000120010000100000000001100010010000000

*Limnofregata* 2?0001000A0???0?1??02020010000??0?00??00

*Fregata minor* 2200010000001101112020201100010012000000

*Fregata ariel* 2200010000001101112020201100010012000000

*Fregata magnificens* 2200010000001101112020201100010012000000

*Papasula abbotti* 000002011010110012000200000000?000000100

*Sula sula* 100002011010010012A011000000001010000000

*Sula leucogaster* 1000020110100100121011000000001010000000

*Sula dactylatra* 1000020110100100121011000000001010000000

*Sula variegata* 1000020110100100121011000000001010000000

*Sula nebouxii* 1000020110100100121011000000001010000000

*Morus serrator* 0000020110100100120000000000001010110000

*Morus bassanus* 0000020110100100120000000000001010110000

*Morus_capensis* 0000020110100100120000000000001010110000

*Phocavis maritimus* ????????????????????????????????????????

*Plotopterum joaquinensis* ????????????????????????????????????????

*Copepteryx hexeris* 00010200?2?-200--B01---0000?1??000??--?0

*Tonsala hildegardae* ??010200?20-?00--??1-??0000?1??00??????0

*Anhinga anhinga* 0111020110001100010000010000000001000000

*Anhinga rufa* 0111020110001100010000010000000001000000

*Borvocarbo stoeffelensis* ????????????????????????????????????????

*Phalacrocorax africanus* 0111020110011000110002010000000001000101

*Phalacrocorax pygmaeus* 0111020110011000110002010000000001000101

*Phalacrocorax melanoleucos* 0111020110011000110002010000000001000101

*Phalacrocorax auritus* 0111020110011000110002010000000001000101

*Phalacrocorax brasilianus* 0111020110011000110002010000000001000101

*Phalacrocorax gaimardi* 0111020110011000110002010000000001000101

*Phalacrocorax carbo* 0111020110011000110002010000000001000101

*Phalacrocorax penicillatus* 0111020110011000110002010000000001000101

*Phalacrocorax pelagicus* 0111020110011000110002010000000001000101

*Phalacrocorax aristotelis* 0111020110011000110002010000000001000101

*Phalacrocorax bougainvilli* 0111020110011000110002010000000001000101

*Phalacrocorax magellanicus* 0111020110011000110002010000000001000101

*Phalacrocorax atriceps* 0111020110011000110002010000000001000101

## *Phalacrocorax albiventer* 0111020110011000110002010000000001000101_

_______________________________________________________________________

Character 2

8

## Taxa 0_

*Eudromia elegans* 00000000000000000000010100000?000012000-

*Gallus gallus* 00000000000000000000010100000?0001110000

*Gavia immer* 1010100000100000010001000000011200021001

*Podiceps auritus* 1000100000100000000000000100011000020000

*Pygoscelis antarctica* 1000---1000-0001100010000000000110101000

*Eudyptula minor* 1000---1000-000110001000000000011010100?

*Phoebastria nigripes* 1110100200000000011001000100000000020000

*Puffinus gravis* 1110100200100000011001000100000000020000

*Pelecanoides urinatrix* 1110100200100000011000000100010000020000

*Oceanodroma castro* 1110100200100000011001000100010000020000

*Oceanites oceanicus* 1110100200100000011001000100010000020000

*Lithoptila abdounensis* 1??0100?00?0???00???????????????????????

*Prophaethon shrubsolei* ????????????????????????????????????????

*Phaethon rubricauda* 1000100200000000001002100100000001020011

*Phaethon lepturus* 1000100200000000001002100100000001020011

*Phaethon aethereus* 1000100200000000001002100100000001020011

*Phoenicopterus chilensis* 1000100000000000000001000100000000020000

*Ciconia abdimii* 1001101000000000020001000100000002120000

*Cochlearius cochlearius* 1000101000000000000001000100100001120000

*Ardea herodias* 1000101000000000000001000100100001120000

*Eudocimus albus* 1000101000000000000001010100100002110000

*Platalea ajaja* 1000101000000000000001010100100002110000

*Scopus umbretta* 1000001000000000000001000100000002120000

*Balaeniceps rex* 1000101000000000000001010100000002120000

*Pelecanus erythrorhnychos* 1000011000000000000001111101000001120011

*Limnofregata* 10????00??0????0?C0001?1?1??000001020001

*Fregata minor* 1001110000000000020001111111000001020011

*Fregata ariel* 1001110000000000020001111111000001020011

*Fregata magnificens* 1001110000000000020001111111000001020011

*Papasula abbotti* 100110001000000000000???0101?0?001020011

*Sula sula* 1001100010000000000001110101000001020011

*Sula leucogaster* 1001100010000000000001110101000001020011

*Sula dactylatra* 1001100010000000000001110101000001020011

*Sula variegata* 1001100010000000000001110101000001020011

*Sula nebouxii* 1001100010000000000001110101000001020011

*Morus serrator* 1001000010100000000001110101000001020011

*Morus bassanus* 1001000010100000000001110101000001020011

*Morus_capensis* 1001000010100000000001110101000001020011

*Phocavis maritimus* ????????????????????????????????????????

*Plotopterum joaquinensis* ????????????0???????????????????????????

*Copepteryx hexeris* 1000--01000-?00100001????1?????200??100?

*Tonsala hildegardae* 1000--?1000-0001??00????0?0????2000B????

*Anhinga anhinga* 1000000001010000031101011000100001020001

*Anhinga rufa* 1000000001010000031101011000100001020001

*Borvocarbo stoeffelensis* ??????????????????????????????????0?????

*Phalacrocorax africanus* 1000100001011010030101000000100001010101

*Phalacrocorax pygmaeus* 1000100001011010030101000000100001010101

*Phalacrocorax melanoleucos* 1000100001011010030101000000100001010101

*Phalacrocorax auritus* 1000100001011110030101000000100001010101

*Phalacrocorax brasilianus* 1000100001011110030101000000100001010101

*Phalacrocorax gaimardi* 1000100001011110030101000000100001010101

*Phalacrocorax carbo* 1000100001011110030101000000100002010101

*Phalacrocorax penicillatus* 1000100001011110030101000000100001010101

*Phalacrocorax pelagicus* 1000100001011110030101000000100001010101_

_______________________________________________________________________

Character 2

8

## Taxa 0_

*Phalacrocorax aristotelis* 1000100001011110030101000000100001010101

*Phalacrocorax bougainvilli* 1000100001011110030101000000100001010101

*Phalacrocorax magellanicus* 1000100001011110030101000000100001010101

*Phalacrocorax atriceps* 1000100001011110030101000000100001010101

## *Phalacrocorax albiventer* 1000100001011110030101000000100001010101_

_______________________________________________________________________

Character 3

2

## Taxa 0_

*Eudromia elegans* 000001000000000000??0000-000000000000000

*Gallus gallus* 0000000000000000000000001000000000010020

*Gavia immer* 000000000000000100121--00001010000030002

*Podiceps auritus* 000002000000010100121--00001010000030000

*Pygoscelis antarctica* 0000020110000100000001-00001110110010000

*Eudyptula minor* 000002011?000100000001-00001110110010000

*Phoebastria nigripes* 0000010000000100000000000001000010020001

*Puffinus gravis* 0000010000000100000000000001010010020001

*Pelecanoides urinatrix* 0000010000000100000101-00001100000010001

*Oceanodroma castro* 0000010000000100000001-00001100000010001

*Oceanites oceanicus* 0000010000000100000101-00001100000010001

*Lithoptila abdounensis* ????????????????????????????????????????

*Prophaethon shrubsolei* ????????????????????????????????????????

*Phaethon rubricauda* 0001000000100000100101-00001100100010001

*Phaethon lepturus* 0001000000100000100101-00001100100010001

*Phaethon aethereus* 0001000000100000100101-00001100100010001

*Phoenicopterus chilensis* 0001000000000000100000000011001000010000

*Ciconia abdimii* 0000000000000000100000010011000100010000

*Cochlearius cochlearius* 0000020000000100100001-01011000000010120

*Ardea herodias* 0000020000000100100001-01011000000010120

*Eudocimus albus* 0100000000000000100000001111000000010002

*Platalea ajaja* 0000000000000000000000001011000000010002

*Scopus umbretta* 0000000000000000000000001111000010010020

*Balaeniceps rex* 0000020000000100100000011011000010020000

*Pelecanus erythrorhnychos* 1010121000000110101100010001000110120010

*Limnofregata* ?000?21001001?00?????0A???1??????0?1????

*Fregata minor* 1010121001111100100101-10011101100111000

*Fregata ariel* 1010121001111100100101-10011101100111000

*Fregata magnificens* 1010121001111100100101-10011101100111000

*Papasula abbotti* 0010121001100110101100110011000100120010

*Sula sula* 1010121001100110101100110011000100120010

*Sula leucogaster* 1010121001100110101100100011000100120010

*Sula dactylatra* 1010121001100110101100100011000100120010

*Sula variegata* 1010121001100110001100100011000100120010

*Sula nebouxii* 1010121001100110001100100011000100120010

*Morus serrator* 0010121001100110101100110011000100120010

*Morus bassanus* 0010121001100110001100110011000100120010

*Morus_capensis* 0010121001100110001100110011000100120010

*Phocavis maritimus* ????????????????????????????????????????

*Plotopterum joaquinensis* ????????????????????????????????????????

*Copepteryx hexeris* ???0???????????????????????????101010???

*Tonsala hildegardae* ????0???????????????????????????????????

*Anhinga anhinga* 0100020000000100011100100001000101120010

*Anhinga rufa* 0100020000000100011100100001000101120010_

_______________________________________________________________________

Character 3

2

## Taxa 0_

*Borvocarbo stoeffelensis* ????????????????????????????????????????

*Phalacrocorax africanus* 0100020000000100001100100001000101130012

*Phalacrocorax pygmaeus* 0100020000000100001100100001000101130012

*Phalacrocorax melanoleucos* 0100020000000100001100100001000101130012

*Phalacrocorax auritus* 0100020000000100001100100001000101130012

*Phalacrocorax brasilianus* 0100020000000100001100100001000101130012

*Phalacrocorax gaimardi* 0100020000000100001100100001000101130012

*Phalacrocorax carbo* 0100020000000100001100100001000101130012

*Phalacrocorax penicillatus* 0100020000000100001100100001000101130012

*Phalacrocorax pelagicus* 0100020000000100001100100001000101130012

*Phalacrocorax aristotelis* 0100020000000100001100100001000101130012

*Phalacrocorax bougainvilli* 0100020000000100001100100001000101130012

*Phalacrocorax magellanicus* 0100020000000100001100100001000101130012

*Phalacrocorax atriceps* 0100020000000100001100100001000101130012

## *Phalacrocorax albiventer* 0100020000000100001100100001000101130012_

_______________________________________________________________________

Character 3

6

## Taxa 0_

*Eudromia elegans* 001000000000000000000000-000000000000-00

*Gallus gallus* 001001001000100000000000-000000000100000

*Gavia immer* 001101001010000100000010-000100100120001

*Podiceps auritus* 000101000000000100000000-000100100100001

*Pygoscelis antarctica* 000201000010001000000100-000010100100100

*Eudyptula minor* 000201000010001000000100-000010100100100

*Phoebastria nigripes* 000010100000001000100100-000000000120001

*Puffinus gravis* 000010110000001100100110-0000000000---01

*Pelecanoides urinatrix* 000010110000101100100110-0000000000---01

*Oceanodroma castro* 000010110000101100100100-000000000120001

*Oceanites oceanicus* 000010110000101100100110-000000000120001

*Lithoptila abdounensis* ???????????????000?0010???0????00???????

*Prophaethon shrubsolei* ???????????????????????????????????????0

*Phaethon rubricauda* 000001000000010000100100-001000000111000

*Phaethon lepturus* 000001000000010000100100-001000000111000

*Phaethon aethereus* 000001000000010000100100-001000000111000

*Phoenicopterus chilensis* 010001000000000010000000-000000011103000

*Ciconia abdimii* 110001000000000010000000-000000001100000

*Cochlearius cochlearius* 010001000000000000000000-000000011100000

*Ardea herodias* 010001000000000000000000-000000011100000

*Eudocimus albus* 110001000000000000000000-000000011100000

*Platalea ajaja* 110001000000000000000000-000000011100000

*Scopus umbretta* 110001000000000000000000-000000000100000

*Balaeniceps rex* 010001000000000000000000-000000000100000

*Pelecanus erythrorhnychos* 000001000000010000100100-000000000110000

*Limnofregata* ???0?10??00?00??1???100???0000?????????0

*Fregata minor* 010001000101001010001000-000000020110000

*Fregata ariel* 010001000101001010001000-000000020110000

*Fregata magnificens* 010001000101001010001000-000000020110000

*Papasula abbotti* 00000100010101001010110?-0100?0000110100

*Sula sula* 000001000101011010101100-100000000110100

*Sula leucogaster* 001001000101011010101100-110000000110100

*Sula dactylatra* 201001000101011010101100-110000000110100

*Sula variegata* 201001000101011010101100-110000000110100_

_______________________________________________________________________

Character 3

6

## Taxa 0_

*Sula nebouxii* 201001000101011010101100-110000000110100

*Morus serrator* 000001000101010010001100-000010000110100

*Morus bassanus* 000001000101010010001100-000000000110100

*Morus_capensis* 000001000101010010001100-000000000110100

*Phocavis maritimus* ????????????????????????????????????????

*Plotopterum joaquinensis* ????????????????????????????????????????

*Copepteryx hexeris* ?0?0010???0??1000000110???00000000????00

*Tonsala hildegardae* ??????????????????????????????????1?01??

*Anhinga anhinga* 001001000101010000101100-000000000110110

*Anhinga rufa* 001001000101010000101100-000000000110110

*Borvocarbo stoeffelensis* ????????????????????????????????????????

*Phalacrocorax africanus* 0000010001010000001001110000000000112100

*Phalacrocorax pygmaeus* 00?0010001010000001?01111000000000112100

*Phalacrocorax melanoleucos* 00?0010001010000001?01111000000000112100

*Phalacrocorax auritus* 0010010001010000001101111000000000112100

*Phalacrocorax brasilianus* 0010010001010000001101111000000000112100

*Phalacrocorax gaimardi* 001001000101000000110110-000000000112100

*Phalacrocorax carbo* 0010010001010000001101111000000000112100

*Phalacrocorax penicillatus* 0010010001010000001101111000000000112100

*Phalacrocorax pelagicus* 0010010001010000011101110000001000112100

*Phalacrocorax aristotelis* 0010010001010000001101110000000000112100

*Phalacrocorax bougainvilli* 0010010001010000001101110000000000112100

*Phalacrocorax magellanicus* 0010010001010000011101111000001000112100

*Phalacrocorax atriceps* 0010010001010000001101110000001000112100

## *Phalacrocorax albiventer* 0010010001010000001101110000001000112100_

_______________________________________________________________________

Character 4

0

## Taxa 0_

*Eudromia elegans* 0000001000000002020000001000000000000000

*Gallus gallus* 000000100000000000000000100010A000001000

*Gavia immer* 1000003000000011000111001000001000002101

*Podiceps auritus* 1000003000000010000101000100000000002100

*Pygoscelis antarctica* 0001003001000000000010001000001000002100

*Eudyptula minor* 0001003001000000000010001000001000002100

*Phoebastria nigripes* 0000012000000000001000000110101000002110

*Puffinus gravis* 0000010000000000001000000110101000002110

*Pelecanoides urinatrix* 0001013000000000001000000110100000002010

*Oceanodroma castro* 0000013000000000000000000110100000002010

*Oceanites oceanicus* 0000013000000000000000000110100000002010

*Lithoptila abdounensis* ????????????????????????????????????????

*Prophaethon shrubsolei* 000?01D000??????????????????????????????

*Phaethon rubricauda* 0101003000001200001000000000000000102110

*Phaethon lepturus* 0101003000001200001000000000000000102110

*Phaethon aethereus* 0101003000001200001000000000000000102110

*Phoenicopterus chilensis* 0001103000000000001000010000010010000000

*Ciconia abdimii* 0001003000000000001000010000010010000000

*Cochlearius cochlearius* 0000003100000000000000000000110000000000

*Ardea herodias* 0000003100000000000000000000110000000000

*Eudocimus albus* 0001003000000000001000010000110010000000

*Platalea ajaja* 0001003000000000001000010000110010000000

*Scopus umbretta* 0001003000000000000000000000010000000000

*Balaeniceps rex* 0001003000001000000000000000010000000000_

_______________________________________________________________________

Character 4

0

## Taxa 0_

*Pelecanus erythrorhnychos* 0001003000001101010010000000000000102100

*Limnofregata* 000?0030?0???10??A??1000???0?????110?1??

*Fregata minor* 0101003000001101000100000000100001102100

*Fregata ariel* 0101003000001101000100000000100001102100

*Fregata magnificens* 0101003000001101000100000000100001102100

*Papasula abbotti* 0001001000000101010110000000000000102100

*Sula sula* 0001001000000101010010000000000000102100

*Sula leucogaster* 00010010000001010100100000000000001A2100

*Sula dactylatra* 0001001000000101010010000000000000112100

*Sula variegata* 0001001000000101010010000000000000112100

*Sula nebouxii* 0001001000000101010010000000000000112100

*Morus serrator* 0011101000000101010010100000000000102100

*Morus bassanus* 0011101000000101010010100000000000102100

*Morus_capensis* 0011101000000101010010100000000000102100

*Phocavis maritimus* ????????????????????????????????????????

*Plotopterum joaquinensis* ????????????????????????????????????????

*Copepteryx hexeris* 000?00?000?00?0??1??10????0?10100010????

*Tonsala hildegardae* ????????????????????????????????????????

*Anhinga anhinga* 0001001010010101110110001001101000102101

*Anhinga rufa* 0001001010010101110110001001101000102101

*Borvocarbo stoeffelensis* ????????????????????????????????????????

*Phalacrocorax africanus* 0001001010110111010110000001101100102101

*Phalacrocorax pygmaeus* 0001001010110111010110000001101100102101

*Phalacrocorax melanoleucos* 0001001010110111010110000001101100102101

*Phalacrocorax auritus* 0001001010110111010120000001101100102101

*Phalacrocorax brasilianus* 0001001010110111010120000001101100102101

*Phalacrocorax gaimardi* 0001001010110111010120000001101100102101

*Phalacrocorax carbo* 0001001010110111010120000001101100102101

*Phalacrocorax penicillatus* 0001001010110111010120000001101100102101

*Phalacrocorax pelagicus* 0001001010110111010120000001101100102101

*Phalacrocorax aristotelis* 0001001010110111010120000001101100102101

*Phalacrocorax bougainvilli* 0001001010110111010120000001101100102101

*Phalacrocorax magellanicus* 0001001010110111010120000001101100102101

*Phalacrocorax atriceps* 0001001010110111010120000001101100102101

## *Phalacrocorax albiventer* 0001001010110111010120000001101100102101_

_______________________________________________________________________

Character 4

4

## Taxa 0_

*Eudromia elegans* 0000?00000000000000000-00-0000000--00111

*Gallus gallus* 0000000000010000000000100000000000000000

*Gavia immer* 1102100000110100000110000001010100000010

*Podiceps auritus* 0102100000110100000100100101010100000010

*Pygoscelis antarctica* 1001100201020001000000000010010000030110

*Eudyptula minor* 1001100200000001000000000010010000030100

*Phoebastria nigripes* 0002100000010000000100000000010000000010

*Puffinus gravis* 0002100000110000000100000100010000000010

*Pelecanoides urinatrix* 0002100000000000000100100100010000000010

*Oceanodroma castro* 0002100000000000000100100100010000000010

*Oceanites oceanicus* 0002100000110000000100100100010000000010

*Lithoptila abdounensis* ??????0??0?????????????????0?00?100?10?0

*Prophaethon shrubsolei* ????????????????????????????????????????

*Phaethon rubricauda* 0000001000020000000101000000000010001010_

_______________________________________________________________________

Character 4

4

## Taxa 0_

*Phaethon lepturus* 0000001000020000000101000000000010001010

*Phaethon aethereus* 0000000000020000000101000000000010001010

*Phoenicopterus chilensis* 0002000000000010010100000000000000000000

*Ciconia abdimii* 0002000000000010010100000000000001000000

*Cochlearius cochlearius* 0012000000000000000000100100100101000000

*Ardea herodias* 0012000000000000000000100100100101000000

*Eudocimus albus* 0002000000000000010100000000100001000000

*Platalea ajaja* 0002?00000000000010100000000000001000000

*Scopus umbretta* 0002000000000000000100100100000000000000

*Balaeniceps rex* 0002000000000010000000100100000001000000

*Pelecanus erythrorhnychos* 0000000000000001100000110100010011000001

*Limnofregata* ???10002000010000?10000??1000000?1000?0?

*Fregata minor* 1001000201001100001000100100000001000001

*Fregata ariel* 1001000201001100001000100100000001010001

*Fregata magnificens* 1001000201001100001000100100000001010001

*Papasula abbotti* 100000010000100000000010?100010011001001

*Sula sula* 1000000100001000000000110100010011000001

*Sula leucogaster* 1000000100001000100000111100010011000001

*Sula dactylatra* 1000000100001000100000111100010011000001

*Sula variegata* 1000000100001000100000111100010011000001

*Sula nebouxii* 1000000100001000100000111100010011000001

*Morus serrator* 1000000200001000000000100100000011000001

*Morus bassanus* 1000000200001000000000100100000011000001

*Morus_capensis* 1000000200001000000000100100000011000001

*Phocavis maritimus* ??????0111021100001000100110000001100?0?

*Plotopterum joaquinensis* ????????????????????????????????????????

*Copepteryx hexeris* ???1??0111021100001000000010000001120?01

*Tonsala hildegardae* ????????????????????????????????????????

*Anhinga anhinga* 1000010000021000001000100100110011000001

*Anhinga rufa* 1000010000021000001000100100010011000001

*Borvocarbo stoeffelensis* ???00?01??0???????1?0??0???001????0???0?

*Phalacrocorax africanus* 1000010000021000000010100100021111000001

*Phalacrocorax pygmaeus* 1000000000021000000010100100021111000001

*Phalacrocorax melanoleucos* 1000000000021000000010100100021111000001

*Phalacrocorax auritus* 1000010000021000000010100100011111000001

*Phalacrocorax brasilianus* 1000010000021000000010100100011111000001

*Phalacrocorax gaimardi* 1000010000021000000010100100011111000001

*Phalacrocorax carbo* 1000010000021000000010100100011111000001

*Phalacrocorax penicillatus* 1000010000021000000010100100011111000001

*Phalacrocorax pelagicus* 1000010000021000000010100100011111000001

*Phalacrocorax aristotelis* 1000010000021000000010100100011111000001

*Phalacrocorax bougainvilli* 1000010000021000000010100100011111000001

*Phalacrocorax magellanicus* 1000010000021000000010100100011111000001

*Phalacrocorax atriceps* 1000010000021000000010100100011111000001

## *Phalacrocorax albiventer* 1000010000021000000010100100011111000001_

_______________________________________________________________________

Character 4

6

## Taxa 4 __

*Eudromia elegans* 1000A0B00000000000000000

*Gallus gallus* 100010000000000000000000

*Gavia immer* 000011000000000000000000

*Podiceps auritus* 000011000000000002100100

*Pygoscelis antarctica* 000021000001000000100000

*Eudyptula minor* 000021000001000000100000

*Phoebastria nigripes* 000011B00001000000101010

*Puffinus gravis* 0000111000010000001010?0

*Pelecanoides urinatrix* 0000112-00010000001010?0

*Oceanodroma castro* 0000111000010000001010?0

*Oceanites oceanicus* 0000111000010000001010?0

*Lithoptila abdounensis* 1?000???????????????????

*Prophaethon shrubsolei* ????????????????????????

*Phaethon rubricauda* 110021000001001000001010

*Phaethon lepturus* 110021000001001000001010

*Phaethon aethereus* 110021000001001000001010

*Phoenicopterus chilensis* 0000?0000000000000100100

*Ciconia abdimii* 100020010000010000001000

*Cochlearius cochlearius* 000020010110010010010000

*Ardea herodias* 000020010110010010010000

*Eudocimus albus* 100020010000010000000000

*Platalea ajaja* 10002?010000010000000000

*Scopus umbretta* 000020000000000000000000

*Balaeniceps rex* 100000000000000000000000

*Pelecanus erythrorhnychos* 101021010000000000010000

*Limnofregata* ??003001000?000000000000

*Fregata minor* 100030010000000000000000

*Fregata ariel* 100030010000000000000000

*Fregata magnificens* 100030010000000000000000

*Papasula abbotti* 111131000000000001001000

*Sula sula* 111031000000000001001000

*Sula leucogaster* 111131000000000001001000

*Sula dactylatra* 111131000000100001001000

*Sula variegata* 111131000000?00001001000

*Sula nebouxii* 111131000000100001001000

*Morus serrator* 111031000000000001001000

*Morus bassanus* 111031000000000001001000

*Morus_capensis* 111031000000000001001000

*Phocavis maritimus* ?01?2???????????????????

*Plotopterum joaquinensis* ????????????????????????

*Copepteryx hexeris* 10112???????????????????

*Tonsala hildegardae* ????????????????????????

*Anhinga anhinga* 111031010000001001001000

*Anhinga rufa* 111131010000001001001000

*Borvocarbo stoeffelensis* ???13201000??0??010?1?01

*Phalacrocorax africanus* 111132010000001101001001

*Phalacrocorax pygmaeus* 111132010000001101001001

*Phalacrocorax melanoleucos* 111132010000001101001001

*Phalacrocorax auritus* 111122010000001101001001

*Phalacrocorax brasilianus* 111122010000001101001001

*Phalacrocorax gaimardi* 1111?201?000001101001001

*Phalacrocorax carbo* 111122010000001101001001

*Phalacrocorax penicillatus* 111122010000001101001001

*Phalacrocorax pelagicus* 111122010000001101001001_

_______________________________________________________________________

Character 4

6

## Taxa 4 __

*Phalacrocorax aristotelis* 111122010000001101001001

*Phalacrocorax bougainvilli* 111122011000001101001001

*Phalacrocorax magellanicus* 1111?2011000001101001001

*Phalacrocorax atriceps* 11112201?000001101001001

*Phalacrocorax albiventer* 111122011000001101001001
